# Quantum Systems at The Brink: Helium–Type Systems

**DOI:** 10.1007/s00205-026-02167-7

**Published:** 2026-07-13

**Authors:** Dirk Hundertmark, Michal Jex, Markus Lange

**Affiliations:** 1 Department of Mathematics, Institute for Analysis, Karlsruhe Institute of Technology, 76128 Karlsruhe, Germany; 2 Department of Mathematics, Altgeld Hall, University of Illinois at Urbana-Champaign, 1409 W. Green Street, Urbana, IL 61801 US; 3 Department of Physics, Faculty of Nuclear Sciences and Physical Engineering, Czech Technical University in Prague, Břehová 7, 11519 Prague, Czech Republic; 4 CEREMADE, Université Paris-Dauphine, PSL Research University, Place de Lattre de Tassigny, 75016 Paris, France; 5 German Aerospace Center (DLR), Institute for AI-Safety and Security, German Aerospace Center, Sankt Augustin & Ulm, Germany

**Keywords:** 81Q05, 35Q40, 81V45

## Abstract

In the present paper we study two challenging problems for helium–type systems: the existence of eigenvalues at thresholds and the asymptotic behavior of the corresponding eigenfunctions. Since the usual methods for addressing these problems need a safety distance to the essential spectrum, they cannot be applied in critical cases, when an eigenvalue enters the continuum. We develop a method to address both problems and derive *sharp upper and lower bounds* for the asymptotic behavior of the ground state of critical helium–type systems at the threshold of the essential spectrum. This is the first proof of the precise asymptotic behavior of the ground state for this benchmark problem in quantum chemistry. Moreover, our bounds describe precisely how the asymptotic decay of the ground state changes, when the system becomes critical.

## Introduction

From the beginning of quantum mechanics, many important questions about a quantum system are related to the existence and behavior of its bound states. These states are given by the square integrable eigenvectors of the operator describing the quantum system. In this paper we consider two particle operators of the form

1.1 $$\begin{aligned} H_U = P_1^2 + P_2^2 - \frac{1}{|x_1|} - \frac{1}{|x_2|} + \frac{U}{|x_1 - x_2|}\, . \end{aligned}$$

Here $x_j\in {\mathbb {R}}^3$ are the positions of the two particles, and $P_j^2$ their kinetic energy, where $P_j=-i\nabla _{x_j}$ is the momentum operator of the particle and j=1,2. The Hamiltonian $H_U$ is well-defined and self-adjoint on $\mathcal {D}(H_U)=H^2({\mathbb {R}}^6)$, where $H^2({\mathbb {R}}^6)$ is the usual Sobolev space of weakly differentiable functions in $L^2({\mathbb {R}}^6)$ with square integrable weak derivatives up to second order.

The operator $H_U$ arises from scaling two–particle helium–type systems described by the operator

$$\begin{aligned} H= \sum _{j=1}^2\left( \frac{1}{2m} P_j^2- \frac{Ze^2}{|x_j|} \right) + \frac{e^2}{|x_1-x_2|}, \end{aligned}$$

where *Z* is the nuclear charge and *e* the unit for the elementary electric charge. Here we use units, where other physical constants such as Planck’s constant ħ are set equal to one. Using scaling, i.e., a change of length–scale, given by $U_s\psi (x)= s^3 \psi (s x)$, $x\in {\mathbb {R}}^6$, which is unitary on $L^2({\mathbb {R}}^6)$, one gets, with $s=2mZe^2$ and U=1/Z, that

$$\begin{aligned} U_s^* H U_s = 2mZ^2e^4 H_U \, . \end{aligned}$$

Thus $H_U$ with U=1/Z is the operator describing helium-type systems such as $Li^+$ for Z=3, *He* for Z=2, and $H^-$ for Z=1.

We denote the ground state energy of $H_U$ by $E_U$. More precisely, $E_U=\inf _{\Vert \psi \Vert =1}\langle \psi , H_U\psi \rangle$, which is concave and increasing in U≥0, since $H_U$ is linear in *U* and the two particle Coulomb repulsion is positive. Hence the ground state energy $E_U$ is continuous in U≥0. Of course, one should also include the spin of the particles, e.g., electrons, in which case one should consider $H_U$ on $L^2(({\mathbb {R}}^3\times {\mathbb {C}}^2)^2)$, or the antisymmetric subspace of functions which are antisymmetric when permuting the particles. Since the potential does not couple different spins, all bound states of $H_U$ on $L^2(({\mathbb {R}}^3\times {\mathbb {C}}^2)^2)$ can be classified as two particle bound states with parallel or antiparallel spins. In the first case the wave functions is antisymmetric, in the second case it is symmetric under permutation of the particle *positions*. The ground state of $H_U$ on $L^2(({\mathbb {R}}^3\times {\mathbb {C}}^2)^2)$ lies in the latter, so it is enough to consider $H_U$ on the subspace of $L^2({\mathbb {R}}^3\times {\mathbb {R}}^3)$ which is symmetric under permutation of the particle positions, see the discussion in [44, Chapter 4.3].

The HVZ Theorem [46, 36, Theorem XIII.17], shows that the essential spectrum of $H_U$ is a half–line whose bottom is given by the ground state energy of the system with one less electron, i.e., hydrogen whose ground state energy is -1/4. Thus

$$\begin{aligned} {\sigma _{\text {ess}}}(H_U)=\left[ -\frac{1}{4},\infty \right) \end{aligned}$$

for any U≥0. Since usual perturbation theory applies when $E_U<\inf {\sigma _{\text {ess}}}(H_U)=-1/4$, the regular first order perturbation theory [36], also called the Feynman-Hellmann formula, gives

1.2 $$\begin{aligned} \frac{d}{dU} E_U =\langle \psi _U, (\partial _U H_U) \psi _U \rangle = \left\langle \psi _U, \frac{1}{|x_1-x_2|}\psi _U \right\rangle >0 \end{aligned}$$

for all U≥0 with $E_U<-1/4$.

Since the work of Stillinger [43], see also [42], these helium–type atoms at critical coupling are an intensely studied benchmark problem in quantum chemistry. For a review see, e.g., [18] and the references therein. In particular, there has been interest in the precise value of the critical $U_c$, for which the ground state energy of $H_U$ enters the edge of the essential spectrum, that is, $U_c$ is the smallest coupling for which $E_U=-1/4$. Numerically, one finds that, $U_c\simeq 1.1$ [43]. The calculation of $U_c$ and the bound state at critical coupling $U=U_c$ continues to serve as an important benchmark problem in quantum chemistry. E.g., going up to order 401 in perturbation theory, the critical value was calculated in the very nice paper [6] to be approximately $U_c\simeq 1.097 66$, which was further pushed in [29]. A variational calculation of $U_c$ was done in [37]. Recently [13] pushed the calculations to $U_c\simeq 1,097\, 660\, 833\, 738\, 56$. Without the Born–Oppenheimer approximation the critical coupling, which depends on the nuclear mass, was calculated numerically in [30].

It is easy to see that, there is a critical $1<U_c\le 2$ such that $E_{U_c}= \inf {\sigma _{\text {ess}}}(H_U)$. As discussed in Appendix D, $E_U<-1/4$ implies U<2 for this two-particle system. So clearly $U_c\le 2$. To see that $U_c>1$ we note that the classical result by Bethe [9], using a test function ansatz due to Hylleras [28], shows that hydrogen can bind two electrons, i.e., $H_U$ has a ground state with energy <-1/4 for U=1. Hill showed that this is also true without the Born–Oppenheimer approximation, as long as the mass of the nucleus is not too small compared to the mass of the lighter particles. So by continuity of $E_U$ in *U*, one knows that $E_U$ is below the ionization threshold even for some U>1, so $U_c>1$. Moreover, the work of Hill [21] shows that for $1\le U<U_c$ the operator $H_U$ has exactly one bound state with energy below the essential spectrum. This also holds for finite nuclear mass [22].

Although the Hamiltonian $H_U$ with $U=U_c$ has been intensely studied with asymptotic and numerical methods, little is known rigorously. The existence of a ground state $\psi _c$ of $H_{U_c}$ at critical coupling was proved in [23] with the help of PDE methods. An alternative existence proof was given in [15]. General features of the behavior of the ground state energy of quantum systems near coupling constant threshold had been discussed, for example, in [31, 32]. Unfortunately, still very little is known so far concerning *precise quantitative properties* of the ground state $\psi _c$ *at critical coupling*.

This is the main motivation for our work. We provide sharp upper and lower bounds on the asymptotic behavior of the ground state of helium-type atoms at critical coupling. Moreover, we prove a family of sharp upper and lower bounds which are uniform in the coupling $0\le U\le U_c$. Our results provide the first rigorous bounds on the asymptotic behavior of bounds states which correspond to eigenvalues *at the edge of the essential spectrum*. In addition, even in the subcritical case our upper and lower bounds improve on previously known results, since our upper and lower bounds have *the same leading order* and differ only in lower order terms. Our main results are

### Theorem 1.1

(Global upper bound at critical coupling) Given parameters $K_1,K_2>0$, $1/6<\kappa _1<1/2$, and $(3-2\kappa _1)/4<\kappa _2<1$ define

1.3 $$\begin{aligned} F_+(r_1,r_2)= 2(U_c-1)^{1/2}r_1^{1/2} - K_1 r_1^{\kappa _1} +\frac{1}{2} r_2- K_2 r_2^{\kappa _2} \end{aligned}$$

for $r_1,r_2\ge 0$. Then at critical coupling $U=U_c$ the helium atom has a ground state $\psi _c$ with energy -1/4 and we have the pointwise upper bound

1.4 $$\begin{aligned} \psi _c(x)\le C_u \exp \!\big (\! -F_+(|x|_\infty ,|x|_0) \big ) \end{aligned}$$

for the unique positive ground state where the constant $C_u$ depends only on $\kappa _1$, $\kappa _2$, $K_1$, $K_2$.

Here $|x|_0=\min (|x_1|,|x_2|)$, respectively $|x|_\infty =\max (|x_1|,|x_2|)$, is the distance of the particle closer to, respectively farther from, the nucleus.

The matching *lower bound* is provided by

### Theorem 1.2

(Global lower bound at critical coupling) Given parameters $1/6<\kappa _1<1/2<\kappa _2<1$, and $K_1,K_2>0$ define

1.5 $$\begin{aligned} F_-(r_1,r_2)= 2(U_c-1)^{1/2}r_1^{1/2} + K_1 r_1^{\kappa _1} +\frac{1}{2} r_2 + K_2 r_2^{\kappa _2}\, . \end{aligned}$$

Then we have the pointwise lower bound

1.6 $$\begin{aligned} \psi _c(x)\ge C_l \exp \!\big ( -F_-(|x|_\infty ,|x|_0) \big ) \end{aligned}$$

for the unique positive ground state $\psi _c$ of $H_{U_c}$ at critical coupling where the constant $C_l$ depends only on $\kappa _1$, $\kappa _2$, $K_1$, $K_2$. Here again $|x|_0=\min (|x_1|,|x_2|)$ and $|x|_\infty =\max (|x_1|,|x_2|)$.

### Remark 1.3

For $1/6<\kappa _1< 1/2$ we have $1/2< (3-2\kappa _1)/4<2/3$, so the lower bound in Theorem 1.2 holds for a slightly larger range of parameters than the upper bound from Theorem 1.1. Up to the sign of lower order terms $r_1^{\kappa _1}$ and $r_2^{\kappa _2}$, our upper and lower bounds from Theorem 1.1 and Theorem 1.2 perfectly match each other. Thus we get the *precise leading order coefficients* for the asymptotic decay of the ground state for the helium–type systems, unlike previous methods.

Strictly speaking, the constant $C_u$ in the upper bound (1.4) also depends on $\Vert \psi _c\Vert _\infty$. Since the Coulomb potential is in the Kato–class, for a definition see [4, 11, 39], an a–priori bound on $\Vert \psi _c\Vert _\infty$ is well–known, see [39], as soon as the ground state $\psi _c$ exists.

In a similar fashion, the constant $C_l$ in the lower bound (1.6) also depends on local lower bounds for the ground state $\psi _c$. Once the ground state exists it is unique and can be chosen to be strictly positive [17], up to a global phase. Since the Coulomb potential is in the Kato–class, every eigenfunction has a continuous version [39], so by positivity the ground state $\psi _c$ is locally bounded away from zero as soon as it exists.

The existence of a ground state at critical coupling was shown in [23] by PDE methods. The upper bound from Theorem 1.1, even a much simpler version, see Theorem 4.1, allows for a simple variational proof of *existence* of a ground state of helium–type atoms at critical coupling similar to [15]. We can even avoid the Born–Oppenheimer approximation, see Remark 1.7.

Of course, while being sharp, the above upper and lower bounds hold only at critical coupling. We also have upper and lower bounds which hold uniformly in the coupling. For ε and a≥0 we define

1.7 $$\begin{aligned} F_{\varepsilon ,a}(r) = \Big ( \varepsilon +\frac{a^2}{r}\Big )^{1/2}r + \frac{a^2}{\sqrt{\varepsilon }} \big (\ln ( \sqrt{\varepsilon r+a^2} +\sqrt{\varepsilon r} ) -\ln a \big ). \end{aligned}$$

For $0\le U \le U_c$ we define $a_U$ and $\varepsilon _U$ by

1.8 $$\begin{aligned} a_U{:}{=}(U-1)_+^{1/2}, \quad \varepsilon _U{:}{=}-\frac{1}{4} - E_U, \end{aligned}$$

so $\varepsilon _U$ is the ionization energy, the distance of the energy $E_U$ to the edge of the essential spectrum, which is at -1/4. By the HVZ theorem, this is the minimal energy needed to move one particle to infinity. Note that at critical coupling $U=U_c$ the ionization energy vanishes. Furthermore, we set

1.9 $$\begin{aligned} \begin{aligned} F_U(r){:}{=}F_{\varepsilon _U,a_U}(r) = \Big ( \varepsilon _U +\frac{a_U^2}{r}\Big )^{1/2}r + \frac{a_U^2}{\sqrt{\varepsilon _U}} \big (\ln ( \sqrt{\varepsilon _U r+a_U^2} +\sqrt{\varepsilon _U r} ) -\ln a_U \big ) \end{aligned} \end{aligned}$$

and

1.10 $$\begin{aligned} \begin{aligned} F^U_\pm (r_1,r_2){:}{=}&F_U(r_1) +\frac{1}{2}r_2 \mp \left( K_1r_1^{\kappa _1} + K_2 r_2^{\kappa _2} \right) \, . \end{aligned} \end{aligned}$$

Again, we suppress the explicit dependence of $F^U_\pm$ on the parameters $\kappa _1,\kappa _2$ and $K_1,K_2$, for notational simplicity. We also set $F^U_\pm (x)\!=\! F^U_\pm (x_1,x_2)\!=\! F^U_\pm (|x|_\infty ,|x|_0)$ for $x\!=\!(x_1,x_2)\!\in \! {\mathbb {R}}^3\times {\mathbb {R}}^3$, with a slight abuse of notation.

### Remark 1.4

The heuristic reason why $F_{\varepsilon _U,a_U}$ given in (1.9) captures the asymptotic decay of the ground state $\psi _U$ up to and including criticality will be explained shortly after Theorem 1.5. The precise form of $F_{\varepsilon ,a}$ is of no relevance, what is important is that

1.11 $$\begin{aligned} F_{\varepsilon ,a}'(r) = \left( \varepsilon _U +\frac{a_U^2}{r} \right) ^{1/2}\, . \end{aligned}$$

One can show that

1.12 $$\begin{aligned} \lim _{\varepsilon \rightarrow 0} F_{\varepsilon ,a}(r) = 2a r^{1/2}\, . \end{aligned}$$

In order to get the correct value of $F_{\varepsilon ,a}$ in the limit $\varepsilon \rightarrow 0$, one cannot ignore the logarithmic term in (1.7), which for fixed ε>0 is *always of much lower order* compared to the first term. In the limit of vanishing ionization energy the second term gives the *same contribution* as the first term and should not be discarded, see the discussion in Remark 2.7.

Thus one recovers $F_\pm$ from $F^U_\pm$ in the limit of critical coupling, when the ionization energy vanishes. Clearly, as long as the ionization energy $\varepsilon _U$ is positive, the leading order behavior of $F^U_\pm$ is asymptotic to $F_U(r_1)\sim (\varepsilon _U+ a_U^2/r_1)^{1/2}r_1\sim \sqrt{\varepsilon _U}r_1$, but it changes to $2 a_U\sqrt{r_1}$ *at critical coupling* $U=U_c$.

### Theorem 1.5

(The transition from subcritical to critical: Sharp upper and lower bounds) For any choice of parameters $K_1,K_2>0$, $1/6<\kappa _1<1/2$, and $(3-2\kappa _1)/4<\kappa _2<1$ there exist positive constants $C_\pm$ depending only on $\kappa _1,\kappa _2, K_1,$ $K_2$, and some local bounds on the ground state $\psi _U$ provided in Proposition D.1, such that for the unique positive ground state of the helium-type operator $H_U$ the two–sided pointwise bound

1.13 $$\begin{aligned} C_-\exp \left( -F^U_-(|x|_\infty ,|x|_0) \right) \le \psi _U(x) \le C_+\exp \left( -F^U_+(|x|_\infty ,|x|_0) \right) \end{aligned}$$

holds uniformly in $0\le U\le U_c$.

If one stays away from the critical parameter, i.e., if for fixed small μ>0 the repulsion parameter *U* is allowed to vary uniformly in $\mu \le U\le U_c-\mu$, the range of parameters is bigger. More precisely, assume that $0<\kappa _1<1$ and $1/2< \kappa _2<1$, and $K_1,K_2>0$. Then there exist positive constants $\widetilde{C}_\pm$, depending only on $\kappa _1,\kappa _2, K_1, K_2$, and also μ, such that the two–sided bound

1.14 $$\begin{aligned} \widetilde{C}_-\exp \left( -F^U_-(|x|_\infty ,|x|_0) \right) \le \psi _U(x) \le \widetilde{C}_+\exp \left( -F^U_+(|x|_\infty ,|x|_0) \right) \end{aligned}$$

holds for all $\mu \le U\le U_c -\mu$.

### Remark 1.6

We would like to stress the fact that the constant $C_+$ depends only on the parameters $\kappa _1,\kappa _2, K_1, K_2$ and an a–priori bound on $\Vert \psi _U\Vert _\infty$ for $0\le U\le U_c$, see part of Proposition D.1. Similarly, the constant $C_-$ in (1.13) depends on the parameters $\kappa _1,\kappa _2, K_1, K_2$ and local lower bounds on the ground state $\psi _U$ from part of Proposition D.1 which are uniform in $0\le U\le U_c$. That is, the constants $C_\pm$ in (6.12) are indeed uniform in $0\le U\le U_c$.

Similarly as in Theorem 1.2, the lower bound in (1.13) holds for the slightly larger range of parameters $1/6<\kappa _1<1/2<\kappa _2<1$.

**How to guess the function** $F_{\varepsilon ,a}$: To get an idea what would be the correct ansatz for the decay of the ground state in the regime $U\le U_c$, including criticality, one can argue as follows. By the HVZ theorem, the infimum of the essential spectrum is the system with one particle moved to infinity. In our case one is left with one particle, i.e., hydrogen, whose ground state energy is given by -1/4 in our units. If $U<U_c$, the ground state energy $E_U$ of the two particle system is less than -1/4, i.e., when trying to escape to infinity the farthest out particle experiences an energy penalty given by the ionization energy

1.15 $$\begin{aligned} \varepsilon _U= -1/4 - E_U>0\, , \end{aligned}$$

which is positive. That is, when trying to escape to infinity, the farthest out particle needs to tunnel into an *energetically forbidden region*. WKB–type arguments then predict that the ground state decays as

1.16 $$\begin{aligned} \psi (x)\le C\exp \big (-F(|x|_\infty )) \end{aligned}$$

as long as the inner electron stays close to the nucleus, i.e., $|x|_0$ stays bounded. The function *F* is determined by the energy penalty^1^ it costs to move one particle to infinity,

1.17 $$\begin{aligned} |F'(r)|^2 = \varepsilon _U = -1/4- E_U\, . \end{aligned}$$

Up to a constant term, *F* is clearly given by $F(r)= \sqrt{\varepsilon _U}r$, so the ground state ψ should decay as $\psi (x)\sim \exp (-\sqrt{\varepsilon _U} |x|_\infty )$ when one particle is far away.

Once one particle is removed from the system, a single hydrogen atom with ground state energy $-\frac{1}{4}$ remains and it is known that the ground state of hydrogen decays asymptotically as $\exp (-\frac{1}{2}|x|)$. Thus the decay of the ground state $\psi _U$ of the two particle system, when also the second particle is far away, should be of the form

1.18 $$\begin{aligned} \psi _U(x)\sim \exp \big (-\sqrt{\varepsilon _U}|x|_\infty -\frac{1}{2}|x|_0\big ) \end{aligned}$$

when the system is sub–critical and the ionization energy $\varepsilon _U>0$, i.e., $U<U_c$. Of course, this bound does not survive the limit $U\nearrow U_c$ since $\varepsilon _{U_c}=0$, the ionization energy vanishes at critical coupling!

However, if *U* is close to $U_c$ the farthest out particle sees an *additional energy penalty*! When U>1 and the inner particle is close to the nucleus, the charge of the nucleus, which is one, cannot completely screen the strong repulsion between the electrons. The outer particle, say it is at position $x_1$, sees the attraction to the nucleus but also repulsion from the second particle. Thus when the second particle at position $x_2$ is close to the nucleus the potential energy of the outer particle is

1.19 $$\begin{aligned} -\frac{1}{|x_1|} + \frac{U}{|x_1-x_2|} \approx \frac{U-1}{|x_1|} \end{aligned}$$

when $|x_1|$ is large and $|x_2|$ is close to zero. Hence the outer particle gets a *local boost in energy* of the form

1.20 $$\begin{aligned} \varepsilon _U +\frac{U-1}{|x|_\infty }\, . \end{aligned}$$

That is, tunneling deep into the energetically forbidden region becomes more difficult. The same heuristic arguments as before then predict that the asymptotic decay of the ground state in the position of the outermost particle should be captured by a function *F* satisfying

1.21 $$\begin{aligned} F'(r)= \sqrt{\text {local energy penalty}} = \sqrt{\varepsilon _U +\frac{U-1}{r}}\, . \end{aligned}$$

One can integrate this equation by a tedious calculation. A slightly less tedious calculation shows that for ε,a>0 the function

1.22 $$\begin{aligned} F_{\varepsilon ,a}(r) = \left( \varepsilon +\frac{a^2}{r}\right) ^{1/2}r + \frac{a^2}{\sqrt{\varepsilon }} \big (\ln ( \sqrt{\varepsilon r+a^2} +\sqrt{\varepsilon r} ) -\ln a \big ) \end{aligned}$$

satisfies $F'(r) = \sqrt{\varepsilon +a^2/r}$ for all r>0. Once one particle moved to infinity the remaining system is again a single hydrogen atom, so the same heuristic arguments as before predict that the full two particle ground state $\psi _U$ should decay, for all $0<U\le U_c$, as

1.23 $$\begin{aligned} \psi _U(x) \sim \exp \big (- F_{\varepsilon _U, a_U}(|x|_\infty ) -\frac{1}{2}|x|_0\big )\, \end{aligned}$$

with $\varepsilon _U= -1/4 -E_U$ the ionization energy and $a_U=(U-1)_+^{1/2}$.

In Remark 2.7 we will see that,

1.24 $$\begin{aligned} \lim _{\varepsilon \rightarrow 0+} F_{\varepsilon ,a}(r) = 2a\sqrt{r} \end{aligned}$$

Hence the asymptotic given in (1.23) has a chance of surviving the limit of the parameter *U* becoming critical: At critical coupling the asymptotic of the ground state $\psi _c=\psi _{U_c}$ should be given by

1.25 $$\begin{aligned} \psi _c(x) \sim \exp \left( - 2\sqrt{U_c-1}|x|_\infty ^{1/2} -\frac{1}{2}|x|_0\right) \, . \end{aligned}$$

Thus, at critical coupling, the ground state of helium type system should decay like a stretched exponential. Theorems 1.1, 1.2, and 1.5 show that up to some small lower order correction terms this prediction is indeed correct. Before we embark on the proof we will illustrate in Sect. 2 the main ideas for making the above heuristic rigorous in the one particle case.

To put our results into perspective, let us compare them with previously known results which only addressed the subcritical case $U< U_c$. The first precise bounds for the asymptotic behavior of eigenfunction of $H_U$ with energy strictly below the essential spectrum are due to the groundbreaking works of Slaggie and Wichmann for three–body systems [41], Ahlrichs for atoms [3], O’Connor [35], Combes and Thomas [10], Deift, Hunziker, Simon, and Vock [12] for multi–particle systems, culminating in the work of Agmon [1]. The last result provides bounds for asymptotic behavior of bound states for general multi-particle systems based on energy methods.

For two particle systems in the *subcritical* case, the ionization energy $\varepsilon _U$ is positive and Agmon’s method yields the upper bound

1.26 $$\begin{aligned} |\psi _U(x)| \le C_\delta \exp \left( -c_1|x|_\infty - c_2|x|_0 \right) \end{aligned}$$

for $c_1= \sqrt{\varepsilon _U}-\delta$ and $c_2=1/2-\delta$ for any small δ>0 and some constant $C_\delta$ which diverges for $\delta \rightarrow 0$. Here $|x|_\infty =\max (|x_1|,|x_2|)$ is the distance to the nucleus of the particle *farther from*, and $|x|_0=\min (|x_1|,|x_2|)$ is the distance to the nucleus of the particle *closer to*, the nucleus.

Recall that $\varepsilon _U$ is the ionization energy of the two–particle system. After one particle is removed one is left with hydrogen, whose ionization energy is 1/4 in the units we chose. So both constants $c_1$ and $c_2$ have a clear physical meaning in the upper bound (1.26). Thus, except for reducing the constants by an arbitrarily small amount, the upper bound (1.26) is exactly what is predicted by WKB–type physical heuristics.

Using subsolution estimates, it was shown in [25] that one can set δ=0 at the expense of having polynomial prefactors in the upper bound. A matching lower bound for the ground state ψ, where U=1/Z=1/2, of the two particle system describing helium has been derived by Thomas Hoffmann–Ostenhof in [24],

1.27 $$\begin{aligned} \psi (x) \ge c_\delta \exp \left( -(\sqrt{\varepsilon _1}+\delta )|x|_\infty - \frac{1}{2}|x|_0 \right) \end{aligned}$$

for arbitrary δ>0 and some constant $c_\delta >0$, which goes to zero as $\delta \rightarrow 0$.

Thus, except for decreasing/increasing the constants, which are the square roots of the successive ionization energies, in the upper/lower bounds by an arbitrary amount, these bounds settle the asymptotic behavior of the ground state wave functions and they can also be extended to subcritical $U<U_c$ where $E_U<-1/4$.

Unfortunately, these results are *useless at critical coupling*, since then first ionization energy $\varepsilon _U$ is zero i.e., there is *no energy cost* for removing the first particle. The only known decay property of the ground state $\psi _c=\psi _{U_c}$ of $H_{U_c}$ at critical coupling is the result in [23] where they show that a positive solution of the Schrödinger equation exists and fulfills the upper bound

1.28 $$\begin{aligned} |\psi _c(x)|\le C_m (1+|x|_\infty +|x|_0^2)^{-m} \end{aligned}$$

for some constants $C_m<\infty$ for any m>0 and all $x_1,x_2\in {\mathbb {R}}^3$. Of course, this implies that $\psi _c \in L^2({\mathbb {R}}^6)$, so a ground state for this critical two-particle system exists. Moreover, in the remark in Section 4 of [23], they note that one can use the “Schrödinger inequalities" method of Ahlrichs and M. and T. Hoffmann-Ostenhof [25] to derive sharp upper and lower bounds for the *one-particle density* $\rho _c$ of the ground state $\psi _c$,

1.29 $$\begin{aligned} \begin{aligned} \sqrt{\rho _c(y)}&\le C^+_\delta (1+|y|)^{-3/4+\delta } \exp \left( -2(U_c-1)^{1/2} |y|^{1/2} \right) \, ,\\ \sqrt{\rho _c(y)}&\ge C^-_\delta (1+|y|)^{-3/4-\delta } \exp \left( -2(U_c-1)^{1/2} |y|^{1/2} \right) \end{aligned} \end{aligned}$$

for some constants $0<C^\pm _\delta <\infty$, δ>0 and all $y\in {\mathbb {R}}^3$. Here $\rho _c$ is given by

$$\begin{aligned} \rho _c(y){:}{=}\int _{{\mathbb {R}}^3} |\psi _c(y,z)|^2\, dz; \end{aligned}$$

that is, it is the marginal of the ground state probability density $|\psi _c|^2$ on ${\mathbb {R}}^6$, which is symmetric under permutations of the particles.

However, all these results says nothing about the asymptotic decay of the *full ground state* $\psi _c$. We fill this gap by proving sharp anisotropic upper and lower bounds on the asymptotic behavior of the wave function $\psi _U$ of $H_{U}$ at or below critical coupling and also determine rigorously, how the decay changes in the whole range $0\le U\le U_c$.

### Remark 1.7

We can also treat the case of a finite nuclear mass, i.e., avoid the Born-Oppenheimer approximation. Consider the following three particle system

1.30 $$\begin{aligned} H_U = \frac{p_1^2}{m} + \frac{p_2^2}{m} +\frac{p_N^2}{M}- \frac{1}{|x_1-x_N|} - \frac{1}{|x_2-x_N|} + \frac{U}{|x_1 - x_2|}, \end{aligned}$$

where $p_N=-i\partial _N$ is the momentum of the nucleus, *M* is its mass and $x_N$ is its position. The other two particles have mass *m* and their position are given by $x_1$ respectively $x_2$. The domain of the operator (1.30) is $\mathcal {D}(H_{U})=H^2({\mathbb {R}}^6)\otimes H^2({\mathbb {R}}^3)$.

For finite nuclear mass, M<∞, and $U_c>1$, the helium–type three particle operator 1.30 has a ground state at critical coupling in the center of mass frame, see [19]. The energy of this state is embedded at the edge of the essential spectrum. As for general Schrödinger operators the ground state is unique, up to a global phase, and can be chosen to be positive. With the help of the methods developed in Sect. 4 one can derive isotropic upper bounds for the ground state of this system. We address the problem of sharp anisotropic upper and lower bounds for this system in a forthcoming work.

**Strategy of the proof:** The proof of the upper bound from Theorem 1.1 is via a combination of energy methods, pointwise subsolution type bounds, and a subharmonic comparison principle in the spirit of well–known comparison principles from elliptic PDEs.

By now, the derivation of upper bounds on the asymptotic decay of eigenfunctions with the help of energy methods is standard. However, we would like to stress that, following the general approach a la Agmon [1], one looses an epsilon in the constants of the leading order terms, which we have to avoid. Moreover, the methods of Agmon [1] or [12] and the works before them need a *safety distance* of the ground state energy to the bottom of the essential spectrum. We do not have such a safety distance, since the ground state energy at critical coupling is at the edge of the essential spectrum.

Thus we have to overcome several obstacles. We need a low regularity version of the subharmonic comparison principle which goes back to Agmon [2]. But even having such a low regularity version, we cannot apply it directly. The comparison principle works well for one or two body problems, but not, in general, for multi–particle problems. Even for a restricted three body–body problem such as helium–type atoms in the infinite nuclear mass approximation, the comparison principle is *not directly applicable* because in order to control the errors, *both particles* have to be *far from the nucleus*. Thus subsolution techniques *do not work* well in the case when only one particles tries to escape and the other stays close to the nucleus, see also Section 4 of [2], where it was argued that the subharmonic comparison method is not an effective tool to derive upper bounds on eigenfunctions of multi–particle Schrödinger operators with three or more particles or, as in our case, for a restricted two–body problem with an additional nucleus of infinite mass.

To overcome this we first prove an anisotropic $L^2$ upper bound with the help of energy methods. With standard methods, see [4, 39], this bound can be easily transformed into a pointwise upper bound. Unfortunately, this first upper bound *does not yet yield* the sharp anisotropic upper bound at critical coupling since it *does not have the correct asymptotic* in a *transition region*, see Remark 5.6. Except for this transition region our first anisotropic upper bound has *the correct* asymptotic when one particle is far away and the other one close to the nucleus, or both particles are close to each other and far away. In a second step, we use this a–priori information as an input for our proof of the sharp global anisotropic upper bound where we bridge the transition region with the help of supersolution bounds.

Similarly, the proof of the lower bound is done in two steps. First we derive a sharp lower bound on the ground state well within the region where only one particle tries to escape and the other stays relatively close to the nucleus. We call this the tricky region, the precise definition is given in (3.4). In this case the Coulomb repulsion between the particles yields a boost in energy of the form (1.19) but it does not blow up, since the particles cannot get close to each other in the tricky region, which makes working with subsolutions simpler. We then use this a–priori input to extend the lower bound to the whole region. However, when both particles are not restricted they can come close to each other and when the particles are close together their Coulomb repulsion blows up, which provides a serious obstacle for subsolution bounds.

**Organization of the paper:** As a warm-up, we explain our main new ideas, which prove why a *long-range repulsive* part of the potential can stabilize bound states at the ionization threshold for a one particle system in Sect. 2. We derive sharp upper and lower bounds on the asymptotic behavior of zero energy ground state of such quantum systems.

The necessary local energy bounds, needed for the proof of the anisotropic $L^2$ upper bound for helium–type systems, are derived in Sect. 3. A main new feature here is that we do not use conical regions to localize the particles, which is the usual approach in the study of many-particle systems. Instead, to be able to get a sharp anisotropic upper bound, it is important to use paraboloidal regions.

We derive an isotropic upper bound for the ground state in Sect. 4, which is considerably simpler than the proof our sharp anisotropic bounds. The proof of the anisotropic upper bound is done in Sects. 5 and 6, see, in particular, the proof of Theorem 6.5, which provides the upper bounds in Theorem 1.1 and 1.5.

The proof of the lower bounds is done with a subharmonic comparison principle. As a first step we derive in Sect. 7 a lower bound in the *tricky region*, where one particle tries to escape to infinity and the other one stays (essentially) close to the nucleus. The global anisotropic lower bound is proven in Sect. 8. The main difficulty there is to control the singular Coulomb repulsion between the two particles with terms which are only of lower order. See, in particular, the proof of Theorem 8.4, which provides the lower bounds of Theorem 1.2 and 1.5.

Certain technical tools are gathered in the appendix. The main reason for including them is that our exponential weights for the upper bounds and the comparison functions used in the proofs of the lower bounds lack the usually required high enough regularity. Their derivatives have jumps along codimension one Lipshitz surfaces, so standard results cannot, or at least not straightforwardly, be applied.

## One Particle Case

To explain the main ideas of our approach, we consider one particle moving in an external potential. This external potential consists of an attractive and a repulsive part with Hamiltonian given by

2.1 $$\begin{aligned} H = - \Delta - V + W, \end{aligned}$$

where we assume that V,W≥0 are infinitesimally (form) bounded with respect to -Δ. For simplicity, we also assume that $\textrm{supp}\, V(x)$, the support of *V*, is compact. However, our proof works even for cases where the support of *V* is unbounded provided that the repulsion *W* dominates the attractive part *V* outside some bounded region. We also assume that *W* goes to zero at infinity, so the essential spectrum of the system is given by $\sigma _\textrm{ess}(H) = [0,\infty )$, the discrete spectrum of *H* is below zero.

In the following we assume that the operator H=-Δ-V+W is defined in the sense of quadratic forms, that is,

$$\begin{aligned} \langle \varphi , H\psi \rangle = \langle \nabla \varphi , \nabla \psi \rangle + \langle \varphi , (-V+W)\psi \rangle \end{aligned}$$

for all $\varphi ,\psi$ in the standard Sobolev space $H^1({\mathbb {R}}^d)$. We consider (weak) eigenfunctions $\psi \in H^1({\mathbb {R}}^d)$ of *H*, by which we mean that

2.2 $$\begin{aligned} \langle \varphi , H\psi \rangle = E\langle \varphi ,\psi \rangle \end{aligned}$$

for all $\varphi \in H^1({\mathbb {R}}^d)$. By density of $\mathcal {C}^\infty _0({\mathbb {R}}^d)$ in $H^1({\mathbb {R}}^d)$ it is enough to require that (2.2) holds for all $\varphi \in \mathcal {C}^\infty _0({\mathbb {R}}^d)$. It is relatively straightforward to see that weak eigenfunctions are in the domain of the operator *H*, see Lemma 2.5 in [26], but we will not need this. We are interested in the question whether zero, the edge of the essential spectrum, can be an eigenvalue of the system and if so, how does the corresponding eigenfunction decay at infinity? A typical example is the situation where a parameter, say the repulsion *W* or the depth of the well *V* is tuned in such a way that the ground state eigenvalue hits zero. Does the bound state survive or does it dissipate? This is clearly a non-perturbative situation.

Using previous approaches, such as Agmon’s method [1], one can easily show that the eigenvectors corresponding to negative eigenvalues decay exponentially. However, these approaches need a safety distance to the essential spectrum and yield nothing for eigenfunctions at the edge of the essential spectrum!

We will first discuss how to get upper bounds on eigenfunctions without having a safety distance of the corresponding eigenvalue to the essential spectrum. Once one has such a type of bound, this also implies existence of eigenvalues at the edge of the essential spectrum, Details of this argument are given in Appendix C and D in a more complicated situation.

### Taking Advantage of Long-Range Repulsion

In the following we show how to derive upper bounds on the asymptotic behavior of zero energy eigenfunctions. These upper bounds illustrate how the type of asymptotic decay of ψ is directly related to the repulsive potential *W*.

#### Lemma 2.1

Let *H* be given as in (2.1) and assume that *V* has compact support. Let $\psi \in L^2({\mathbb {R}}^d)$ be a weak eigenfunction of *H* with energy E≤0. Furthermore, assume that F≥0 is a locally bounded and differentiable function such that for some R>0

2.3 $$\begin{aligned} |\nabla F(x)|^2 < W(x) \quad \text {for all } |x|\ge R\, . \end{aligned}$$

Then

2.4 $$\begin{aligned} \int _{|x|\ge R} e^{2F(x)+\ln \big ( W(x)-|\nabla F(x)|^2\big )} |\psi (x)|^2\, dx \le C_{F,R} \Vert \psi \Vert ^2 \end{aligned}$$

with

2.5 $$\begin{aligned} C_{F,R}=8\sup _{R/2\le |x|\le R} e^{2F(x)}\left( 2R^{-2}+ R^{-1}|\nabla F(x)| \right) \, . \end{aligned}$$

#### Remark 2.2

The point of the bound above is that the right hand side is *uniform* in the eigenvalue E≤0. It *does not need a safety distance* to the essential spectrum. Moreover, the constant $C_{F,R}$ depends on *local* bounds of *F*, hence it is finite even for unbounded functions *F*. Of course, as *W* tends to zero at infinity, so does $|\nabla F|^2$. Nevertheless *F* can still go to infinity under this condition, and the main question is which of the terms in $2F+ \ln \big ( W-|\nabla F|^2\big )$ will win this tug–of–war. It is easy to see that the borderline case is a decay of the form $W(x)\sim |x|^{-2}$ at infinity. Any slower decay of *W* will lead to a growth of *F* which out–paces the second term $\ln \big ( W-|\nabla F|^2\big )$.

#### Proof of Lemma 2.1:

Let ξ be any real-valued bounded and differentiable function and use $\varphi =\xi ^2\psi$ in the weak form of the eigenvalue equation. Then $E\Vert \xi \psi \Vert ^2 = E\langle \xi ^2\psi , \psi \rangle = \langle \xi ^2\psi , H\psi \rangle$ and since $E\Vert \xi \psi \Vert ^2$ is real we can use the IMS localization formula, see [11, 20] or Appendix A, to get

2.6 $$\begin{aligned} E\Vert \xi \psi \Vert ^2&= \textrm{Re}\langle \xi ^2\psi , H\psi \rangle = \langle \nabla (\xi \psi ), \nabla (\xi \psi ) \rangle + \langle \xi \psi , (-V+W)\xi \psi \rangle -\langle \psi , |\nabla \xi |^2\psi \rangle \end{aligned}$$

Rearranging and dropping the kinetic energy term, which is positive, gives

2.7 $$\begin{aligned} \langle \xi \psi , (-V+W-E)\xi \psi \rangle \le \langle \psi , |\nabla \xi |^2\psi \rangle \end{aligned}$$

Take $\chi \in \mathcal {C}^\infty ({\mathbb {R}}_+)$ with $0\le \chi \le 1$, χ(r)=0 if r≤1/2, χ(r)=1 if r≥1, and set

2.8 $$\begin{aligned} \chi _R(x){:}{=}\chi \left( |x|/R \right) \quad \text {for }x\in {\mathbb {R}}^d\, . \end{aligned}$$

It is easy to see that a function χ fulfilling the above constraints exists and for which one has $\Vert \chi '\Vert _\infty \le 4$. For any such choice we have $\chi _R\in \mathcal {C}^\infty ({\mathbb {R}}^d)$ and $\Vert \nabla \chi _R\Vert _\infty \le 4/R$.

Now assume that *F* is bounded and differentiable, for the moment, and use $\xi =\xi _R = \chi _R e^{F}$. Using

$$\begin{aligned} \nabla \xi = e^F\nabla \chi _R +e^F\chi _R\nabla F\, , \end{aligned}$$

in (2.7) and reshuffling the terms a bit we see that

2.9 $$\begin{aligned} \langle \xi _R\psi , (-V+W-E-|\nabla F|^2)\xi _R\psi \rangle \le \langle \psi , e^{2F}\left( |\nabla \chi _R|^2+ 2\chi _R \nabla \chi _R\nabla F\right) \psi \rangle \, . \end{aligned}$$

Moreover, we can use $0\le \chi _R\le 1$ on the right hand side and E≤0 to drop the term -E on the left hand side in (2.9) to get

2.10 $$\begin{aligned} \langle \xi _R\psi , (W-|\nabla F|^2)\xi _R\psi \rangle \le \langle \psi , e^{2F}\left( |\nabla \chi _R|^2+ 2|\nabla \chi _R||\nabla F|\right) \psi \rangle \, . \end{aligned}$$

where we also took *R* so large that $\chi _R$, hence also $\xi _R$, is zero on the support of *V*.

In the case that F≥0 is not bounded, we regularize it by considering

2.11 $$\begin{aligned} F_\delta = \frac{F}{1+\delta F}\, . \end{aligned}$$

Then $$F_\delta \le F$$ and

$$\begin{aligned} \nabla F_\delta = \frac{\nabla F}{(1+\delta F)^2} \end{aligned}$$

so $|\nabla F_\delta |\le |\nabla F|$. Thus (2.9) yields

2.12 $$\begin{aligned} \langle \chi _R e^{F_\delta }\psi , (W-|\nabla F|^2)\chi _Re^{F_\delta }\psi \rangle \le \langle \psi , e^{2F}\left( |\nabla \chi _R|^2+ 2|\nabla \chi _R||\nabla F|\right) \psi \rangle \le C_{F,R} \Vert \psi \Vert ^2 \end{aligned}$$

since the support of $\nabla \chi _R$ is contained in the annulus $R/2\le |x|\le R$. Since $0\le \chi _R\le 1$ and $\chi _R(x)=1$ for |x|≥R we get

2.13 $$\begin{aligned} \int _{|x|\ge R} e^{2F_\delta (x)+\ln \big ( W(x)-|\nabla F(x)|^2\big )} |\psi (x)|^2\, dx \le C_{F,R} \Vert \psi \Vert ^2 \end{aligned}$$

and using that $F_\delta$ converges pointwise monotonically to *F* in the limit $\delta \rightarrow 0$ we see that (2.4) holds. □

#### Remark 2.3

Of course, one should not always drop the term -E from the left hand side of (2.9). Keeping it we get the bound

2.14 $$\begin{aligned} \int _{|x|\ge R} e^{2F(x)+\ln \big ( W(x)-E-|\nabla F(x)|^2\big )} |\psi (x)|^2\, dx \le C_{F,R} \Vert \psi \Vert ^2 \end{aligned}$$

under the condition

2.15 $$\begin{aligned} |\nabla F(x)|^2 < W(x)-E \end{aligned}$$

which allows for a larger class of functions *F*, which can grow linearly in |*x*|. This leads to exponential $L^2$-type upper bounds when E<0, i.e., when one has a *safety distance* to the bottom of the essential spectrum. How one can easily choose exponential weights *F* which fulfill condition (2.3), respectively (2.15), is shown in Sects. 2.2 and 2.4.

### An Example with a Repulsive Coulomb Tail

Assume that *V* has compact support and $W(x)= a^2/|x|$ with a>0 outside some compact set. Use

2.16 $$\begin{aligned} F(r)= 2a r^{1/2} - Kr^\kappa /2 \end{aligned}$$

for some 0<κ<1/2 and K>0 and also set F(x)=F(|x|) by a slight abuse of notation. Then $\left|\nabla F\left(x\right)|=\right|F^{\prime }\left(|x|)\right|$ and for any eigenfunction ψ of *H* with energy E≤0, we have

2.17 $$\begin{aligned} W(x)- |\nabla F(x)|^2&= 2 a |x|^{\kappa -3/2} -(K\kappa )^2 |x|^{2(\kappa -1)}/4 \gtrsim |x|^{\kappa -3/2} \ge |x|^{-3/2} \end{aligned}$$

since $\kappa -3/2>2(\kappa -1)$ iff κ<1/2. In this case, Lemma 2.1 shows that

$$\begin{aligned} x\mapsto e^{2a |x|^{1/2} - K|x|^\kappa /2 - \frac{3}{2}\ln |x|}\psi (x) \end{aligned}$$

is in $L^2({\mathbb {R}}^d)$ outside of some large enough ball |x|≥R, uniformly in the energy E≤0 for normalized eigenfunctions ψ. Since any fractional power $r^\kappa$ bounds logarithmic terms lnr for large *r* and ψ is globally $L^2$ we see that

2.18 $$\begin{aligned} x\mapsto e^{2a |x|^{1/2}-K|x|^\kappa }\psi (x)\in L^2({\mathbb {R}}^d) \end{aligned}$$

for any K>0 and 0<κ<1/2.

Using subsolution bounds, or the Harnack inequality for ground states, one can get the pointwise upper bound

2.19 $$\begin{aligned} |\psi (x)| \lesssim \exp (-2a |x|^{1/2} +K|x|^\kappa ) \end{aligned}$$

from this, see the proof of Corollary 5.5.

Moreover, Lemma 2.1 can also be used to show that if the attractive part $V=V_\lambda$ is tuned in the parameter λ in such a way that $H_\lambda = -\Delta -V_\lambda +W$ converges to some limiting operator $H_{\lambda }$ and $H_\lambda$ has a ground state energy $E_\lambda <0$ which converges to zero as $\lambda \rightarrow \lambda _\text {cr}$, then the limiting operator $H_c=H_{\lambda _\text {cr}}$ has a zero energy eigenvalue embedded at the edge of its essential spectrum. This follows from tightness and weak convergence arguments similarly to the discussion in Appendix C and D.

### Lower Bound for Zero Energy Ground States of Systems With a Repulsive Coulomb Tail

Assume that H=-Δ-V+W is as above with $W(x)= a^2/|x|$ outside of a compact, *V* has compact support, and ψ is a positive zero energy ground state of *H*. Then the upper bound in (2.19) is sharp in the sense that we have

#### Lemma 2.4

In the above situation, we have the lower bound

2.20 $$\begin{aligned} \psi (x)\gtrsim \exp (-2a |x|^{1/2} - K|x|^\kappa ) \end{aligned}$$

for all $x\in {\mathbb {R}}^d$, where ψ is the unique positive zero energy ground state of *H*.

#### Proof

We will use the subharmonic comparison principle. Since ψ is only a weak eigenfunction of *H* in the sense of quadratic forms, we use the quadratic form version of the subharmonic comparison principle due to Agmon [2], see also Appendix Appendix B.

Define $g= e^{-F}$, which is $\mathcal {C}^2$ on |x|>R for any R>0 and assume that $g\in L^2$, or at least on |x|>R. Calculating

2.21 $$\begin{aligned} \nabla g = -g\nabla F\quad \text {and } -\Delta g = g\left( \Delta F -|\nabla F|^2 \right) \end{aligned}$$

we see that if

2.22 $$\begin{aligned} W(x)\le |\nabla F(x)|^2 - \Delta F(x) \end{aligned}$$

then Hg≤0 on |x|>R, or, as quadratic forms

2.23 $$\begin{aligned} \langle \varphi , Hg \rangle \le 0 \quad \text {for all } 0\le \varphi \in \mathcal {C}^\infty _0(\Omega _R) \end{aligned}$$

with $\Omega _R= \{|x|>R\}$. Since ψ>0 is continuous (here we assume that *V* and *W* are Kato–class potentials) it is bounded away from zero on $\{|x|\le R\}$, which is compact. Since *g* is bounded, there exist a constant C>0 such that

2.24 $$\begin{aligned} \psi (x)\ge C g(x) \quad \text {for all } |x|\le R+1\, . \end{aligned}$$

Hence on the boundary layer R<|x|<R+1, which is a boundary layer for the boundary $\partial \Omega _R$ in the sense of Definition B.2, we have

2.25 $$\begin{aligned} \psi (x)\ge C g(x) \quad \text {for all } R<|x|<R+1 \end{aligned}$$

and then subharmonic comparison principle implies

2.26 $$\begin{aligned} \psi (x)\ge C g(x) = C\exp (-F(x)) \quad \text {for all } |x|\ge R\, . \end{aligned}$$

By the choice of *C* this also holds for |x|≤R, hence globally.

We apply the above with the radial choice

2.27 $$\begin{aligned} F(r) = 2a r^{1/2} +K r^{\kappa },\quad r=|x| \end{aligned}$$

for which one calculates $\nabla F(x) = F'(r)\frac{x}{|x|}$ and $\Delta F(x)= F''(r)+ F'(r)\frac{d-1}{r}$, hence

2.28 $$\begin{aligned} \begin{aligned} |\nabla&F(x)|^2 - \Delta F(x) -W(x) \\&= 2a K\kappa r^{\kappa -3/2} +(K\kappa )^2 r^{2(\kappa -1)} - a (d-\frac{1}{2}) r^{-3/2} -K\kappa (\kappa +d-2)r^{\kappa -2}>0 \end{aligned} \end{aligned}$$

since the first term dominates the negative terms for all large enough $r_1$ when κ>0. This proves (2.20). □

#### Remark 2.5

A similar calculation shows that $f(x)= \exp (-2a |x|^{1/2}+ K|x|^\kappa )$ is a supersolution at energy zero near infinity. Since eigenfunctions are bounded when the potential is the Kato–class, the subharmonic comparison principle can also be used to see that the matching upper bound

2.29 $$\begin{aligned} \psi (x)\le C \exp (-2a |x|^{1/2}+ K|x|^\kappa ) \end{aligned}$$

holds for all K>0 and 0<κ<1/2. This is the same upper bound as in (2.19) and gives an alternative to the derivation of upper bounds with the help of energy methods for one particle systems. For our upper bounds for helium–type systems we eventually need to use a combination of energy methods and the subharmonic comparison principle.

The lower bound () together with the matching upper bound shows that any zero energy ground state of a one–particle Hamiltonian with a long range Coulomb–type repulsion $W(x)=a^2|x|^{-1}$ obeys for all $x\in {\mathbb {R}}^d$ the two sided bound

2.30 $$\begin{aligned} C_-\exp (-2a |x|^{1/2} - K|x|^\kappa ) \le \psi (x)\le C_+\exp (-2a |x|^{1/2} + K|x|^\kappa ) \end{aligned}$$

for any 0<κ<1/2 and K>0 and some $0<C_-\le C_+<\infty$. In particular, the leading order terms in these bounds are sharp.

Similar sharp lower and upper bounds can be proven for long range repulsive tails of the form $W(x)= a^2 |x|^{-2\rho }$ for any 0<ρ<1. In this case *F* needs to fulfill $F'(r)\sim r^{-\rho }$, hence $F(r)\sim r^{1-\rho }$ and thus any zero energy ground state will have an asymptotic decay of the form

2.31 $$\begin{aligned} \psi (x)\sim \exp \left( -\frac{a}{1-\rho } |x|^{1-\rho }\right) \quad \text {for } |x|\gg 1\, \end{aligned}$$

up to lower order correction terms. We leave the details to the interested reader. A detailed study of the one–particle case can be found in [26], where for short range repulsive tails a phase transition for the non-existence versus existence of zero energy ground states was proved, depending on the dimension d≤3,d=4, and d≥5.

### Repulsive Coulomb-Tail: Interpolating Between the Subcritical and Critical Cases

We can also consider a subcritical situation

2.32 $$\begin{aligned} H_\lambda = -\Delta - V_\lambda (x) + \frac{a^2}{|x|} \end{aligned}$$

with ground state eigenvalue $E_\lambda <0$ for $0\le \lambda <\lambda _\text {cr}$ and $E_\lambda =0$ for $\lambda = \lambda _\text {cr}$. The a–priori bounds above and the discussion in Appendix C and D show that then $H_c=H_{\lambda _\text {cr}}$ has a zero energy bound state. Again, let us assume, for simplicity, that $V_\lambda$ has a compact support. For $\lambda <\lambda _\text {cr}$ the ground states clearly have exponential decay and at critical coupling this exponential decay switches over to the stretched exponential decay with $\lambda =\lambda _\text {cr}$ as discussed above.

The question is how the decay rate changes precisely from exponential to subexponential as the gap between the essential spectrum and the ground state closes for $\lambda \nearrow \lambda _\text {cr}$. In such a case, one should also take advantage of the fact that we can allow $|\nabla F|^2< \eta /|x|-E_\lambda$, see Remark 2.3. With $\varepsilon =-E_\lambda$, the ionization energy, and choosing *F* to be radial, one sees that one needs to find a function $F=F_{\varepsilon ,\eta }$ such that

2.33 $$\begin{aligned} |F_{\varepsilon ,a}'(r)|^2 = \varepsilon +\frac{a^2}{r} \end{aligned}$$

for large enough *r*. A slightly tedious calculation shows that

2.34 $$\begin{aligned} F_{\varepsilon ,a}(r)= \int _0^r\sqrt{\varepsilon +\frac{a^2}{s}}\mathrm d s = r \left( \varepsilon +\frac{a^2}{r}\right) ^{1/2} +\frac{a^2}{\sqrt{\varepsilon }} \left( \ln \left( (a^2 +\varepsilon r)^{1/2} +\sqrt{\varepsilon r}\right) -\ln a \right) \, \end{aligned}$$

which is the reason for the definition (1.7). It is easy to see that the second term in $F_{\varepsilon ,a}(r)$ is positive and only logarithmically growing in *r* for fixed ε>0. Thus $F_{\varepsilon ,a}(r)$ is linearly growing in *r* and asymptotic to $\sqrt{\varepsilon }r$, which is the decay rate predicted by *WKB* methods when the ionization energy is positive.

Using Lemma 2.1, we again conclude that the ground state corresponding to a negative eigenvalue E<0 behaves as

2.35 $$\begin{aligned} \psi (x)\le C_+ \exp \left( -F_{\varepsilon ,a}(|x|)+K|x|^\kappa \right) \end{aligned}$$

for some constant $C_+<\infty$ and any 0<κ<1/2, K>0.

One first uses Lemma 2.1 to get an $L^2$ upper bound and then transfers this into a pointwise upper bound via subsolution bounds of Trudinger, see [39, Theorem C.1.3], or the Harnack inequality for the ground state, similar as in the proof of Corollary 5.5 below.

To derive a lower bound on the ground state ψ, we use the variant

$$\begin{aligned} g=\exp (-F_{\varepsilon ,a}(|x|)-K|x|^\kappa )\, \end{aligned}$$

of the function used in Sect. 2.3. A straightforward but slightly tedious calculation shows that *g* is also a classical subsolution of $H_\lambda = -\Delta -V_\lambda +\eta /|x|$ at energy $E_\lambda =-\varepsilon$ outside a large enough ball in ${\mathbb {R}}^d$, i.e., $(H_\lambda -E_\lambda ) g(x) \le 0$ for |*x*| large enough. We leave the details of these calculations to the interested reader.

Exactly as in the proof of Lemma 2.4 one then concludes that the lower bound

2.36 $$\begin{aligned} \psi (x)\ge C_- \exp \left( -F_{\varepsilon ,a}(|x|)- K|x|^\kappa \right) \end{aligned}$$

holds for some constant $C_->0$ and any 0<κ<1/2, K>0.

Collecting the upper and lower bounds one sees that they yield sharp upper and lower bounds for one–particle quantum systems with a long range Coulomb repulsion.

#### Theorem 2.6

Let $\psi _\lambda$ be a ground state of the one–particle Hamiltonian $H_\lambda$ corresponding to the energy $E_\lambda \le 0$. Then we have the two–sided bound

2.37 $$\begin{aligned} C_1\exp \left( F_{|E_\lambda |,\eta }(|x|)-K|x|^\kappa \right) \le \psi _\lambda \le C_2\exp \left( F_{|E_\lambda |,\eta }(|x|)+K|x|^\kappa \right) \end{aligned}$$

for some constants $0<C_1\le C_2<\infty$ and all 0<κ<1/2, K>1.

The constants $C_1,C_2$ may be dependent on the parameter λ, in general, but will be independent of it for most physically relevant cases; see the end of Remark 2.7 below.

#### Remark 2.7

The logarithmic term in (2.34) is positive and, for fixed ionization energy ε>0 only logarithmically growing in *r*. In this case, the leading order behavior of $F_{\varepsilon ,a}$ is dominated by the first term and is given by

2.38 $$\begin{aligned} F_{\varepsilon ,a}(r)\sim \left( \varepsilon +\frac{a^2}{r}\right) ^{1/2}r\sim \varepsilon ^{1/2}r \end{aligned}$$

for large *r*.

Clearly, $(\varepsilon +a^2/r)^{1/2}r \rightarrow a r^{1/2}$ in the limit of vanishing ionization energy, $\varepsilon =-E\rightarrow 0$, which is exactly half of the leading order term in the estimate (2.30) for the ground state. It is easy to see directly from the definition of $F_{\varepsilon ,a}$ as an integral in (2.34), that

2.39 $$\begin{aligned} F_{0,a}(r) = \lim _{\varepsilon \rightarrow 0}F_{\varepsilon ,a}(r) = 2a r^{1/2} \end{aligned}$$

which is *twice* of the limit of the *leading order term* in the expression of $F_{\varepsilon ,\eta }$ on the right hand side of (2.34). Alternatively, we note that the second term in the right–hand–side of (2.34) can be written as

$$\begin{aligned} \frac{a^2}{\sqrt{\varepsilon }}\ln \left( \left( 1+\frac{\varepsilon r}{a^2}\right) ^{1/2}+\sqrt{\frac{\varepsilon r}{a^2}}\right) = \frac{a\sqrt{ r}}{t} \ln \left( \left( 1+t^2\right) ^{1/2}+t\right) \end{aligned}$$

with $$t= \sqrt{\varepsilon r}/a$$. Since

$$\begin{aligned} \frac{1}{t}\ln \left( \left( 1+t^2\right) ^{1/2}+t\right) = \ln \left( \Big ((1+t+O(t^2)\Big )^{1/t}\right) \rightarrow \ln e =1 \end{aligned}$$

in the limit $t=\sqrt{\varepsilon r}/a \rightarrow 0$, we see that the logarithmic term in (2.34), which for fixed ε>0 is *always of much lower order* than the first term, gives the *same contribution as the first term* in the limit of vanishing ionization energy $\varepsilon \rightarrow 0$ and should not be discarded.

Because of (2.39) the upper and lower bounds (2.35) and (2.36) also hold in the limit of vanishing ionization energy and one recovers our previous subexponential upper and lower bounds for zero energy ground states. Thus the function $F_{\varepsilon ,\eta }$ describes precisely the phase transition between exponential and subexponential decay of the ground states of $H_\lambda$ in the critical limit of vanishing ionization energy.

If the potentials *W* and $V_\lambda$ are in the so-called Kato–class, for definitions see [4, 11, 39], and $V_\lambda$ is continuous in the Kato–norm, one can show that the constants $C_1, C_2$ in (2.37) can be chosen to be *independent of* λ *up to the critical coupling* by an argument which parallels the one we give for helium–type systems, using a one–particle version of Proposition D.1 in the Appendix.

### The Fate of Zero-Energy Solutions for Short Range Potentials

Without a long–range repulsive tail of the potential one can still do a similar analysis as for long–range repulsive potentials, using now an additional boost coming from the kinetic energy. We say that a potential *V* is *short range* if

2.40 $$\begin{aligned} |V(x)|\lesssim \frac{1}{|x|^2 \ln ^q(|x|)} \quad \text {for } |x|\gtrsim 1 \text { and some } q>2\, , \end{aligned}$$

or if

2.41 $$\begin{aligned} |V(x)|\le \frac{c}{|x|^2 \ln ^2(|x|)} \quad \text {for } |x|\gtrsim 1 \text { and some } c<\frac{1}{4} \end{aligned}$$

Let H=-Δ+V be defined via quadratic forms. A function $\psi \in H^1_{\text {loc}}({\mathbb {R}}^d)$ is a (weak) zero energy solution if $\langle \varphi , H\psi \rangle = \langle \nabla \varphi ,\nabla \psi \rangle +\langle \varphi , V\psi \rangle = 0$ for all $\varphi \in \mathcal {C}^\infty _0({\mathbb {R}}^d)$. Clearly, this extends to all $\varphi \in H^1({\mathbb {R}}^d)$ with compact support.

#### Theorem 2.8

Assume that $\psi \in H^1_\text {loc}({\mathbb {R}}^d)$ is a zero energy bound state of a one–particle Schrödinger operator H=-Δ+V with a short–range potential *V* in dimension d≥3 and

2.42 $$\begin{aligned} \liminf _{L\rightarrow \infty } \frac{1}{L^2} \int _{L\le |x|\le \alpha L} |\psi (x)|^2\, dx =0 \end{aligned}$$

for some α>1. Then

2.43 $$\begin{aligned} x\mapsto \frac{(1+|x|)^{(d-4)/2}}{\ln (2+|x|)}\psi (x) \in L^2({\mathbb {R}}^d) \end{aligned}$$

#### Remark 2.9

The condition (2.42) poses a weak condition on the decay of ψ at infinity. In particular, ψ does not need to be in $L^2$. The bound (2.43) shows that if $\textrm{d}\ge 5$, then $\psi \in L^2({\mathbb {R}}^d)$, i.e., ψ is a zero energy eigenfunction of *H*. If d=4, then $x\mapsto (\ln (2+|x|))^{-1}\psi (x)\in L^2({\mathbb {R}}^4)$, i.e., ψ barely misses to be $L^2$ and in three dimensions one sees that $x\mapsto |x|^{-1/2}(\ln (2+|x|))^{-1}\psi (x)\in L^2({\mathbb {R}}^3)$. This is consistent with the known behavior of zero-energy resonances of Schrödinger operators. A series of sharp criteria for the existence/absence of zero energy ground states for one–particle Schrödinger operators in any dimension can be found in [26].

Theorem 2.8 is an extension of results on $L^2$ bounds for zero energy eigenfunctions and resonances of one–particle Schrödinger operators in [8, Theorem 2.1]. As already shown in [8], the above bound has the following consequence: Assume that the potential *V* is infinitesimally form bounded with respect to -Δ and that the one–particle Schrödinger operators H=-Δ+V, defined via quadratic forms, has a virtual level at zero, that is, H≥0 and, with $H_\delta = -(1-\delta )+V$,

$$\begin{aligned} \inf \sigma (H_\delta )<0 \end{aligned}$$

for all 0<δ<1. Then in dimensions d≥5, zero is a simple eigenvalue of *H*. The proof uses Theorem 2.8, or better a slight extension of it to $H_\delta$, and tightness and weak convergence arguments to show that the ground states of $H_\delta$ with δ=1/(2n), $n\in {\mathbb {N}}$, yield a sequence which converges strongly to the ground of *H*. Similar arguments are given in Appendix C and D, for the existence of a ground state for helium–type atoms with a critical repulsion with finite or infinite nuclear mass.

#### Proof of Theorem 2.8

Let *F* be a function which is bounded and differentiable outside some compact set and whose gradient is bounded by $|\nabla F(x)|\lesssim |x|^{-1}$ for large |*x*|. Let $\widetilde{\chi }\in \mathcal {C}^\infty _0({\mathbb {R}}^d)$ with $0\le \widetilde{\chi }\le 1$, $\widetilde{\chi }(x)=1$ if |x|≤1, and $\widetilde{\chi }(x)=0$ if |x|≥2. We use the scaled version $\widetilde{\chi }_L(x)= \widetilde{\chi }(x/L)$, which is a smoothed out projection inside a centered ball of radius ∼L.

Use again $\xi =\chi _R e^F$ with $\chi _R$ from (2.8) and now also $\zeta _L=\widetilde{\chi }_L\xi$. Then $\zeta _L^2\psi$ is in $H^1$ and has compact support. The weak form of the eigenvalue equation and the IMS localization formula again show

2.44 $$\begin{aligned} 0= \langle \zeta _L^2\psi , H\psi \rangle = \langle \zeta _L\psi , H\zeta _L\psi \rangle -\langle \psi , |\nabla \zeta _L|^2\psi \rangle \, . \end{aligned}$$

One calculates

$$\begin{aligned} |\nabla \zeta _L|^2&= |\nabla F|^2\widetilde{\chi }_L^2\chi _R^2e^{2F} +\widetilde{\chi }_L^2 e^{2F} \left( |\nabla \chi _R|^2 +2 \chi _R\nabla \chi _R\nabla F \right) + \chi _R^2 e^{2F} |\nabla \widetilde{\chi }_L|^2 \\&\quad +2 \chi _R\widetilde{\chi }_L \nabla \widetilde{\chi }_L \left( \nabla \chi _R +\chi _R\nabla F \right) e^{2F}\\&= |\nabla F|^2\zeta _L^2 + e^{2F} \left( |\nabla \chi _R|^2 +2 \chi _R\nabla \chi _R\nabla F \right) + \xi ^2 \left( |\nabla \widetilde{\chi }_L|^2 +2 \widetilde{\chi }_L \nabla \widetilde{\chi }_L \nabla F \right) \ \end{aligned}$$

where the last equality holds for L>R, since then $\nabla \widetilde{\chi }_L$ and $\nabla \chi _R$ have disjoint supports and $\widetilde{\chi }_L=1$ on the support of $\nabla \chi _R$. Reshuffling terms in (2.44) we get

2.45 $$\begin{aligned} \begin{aligned} \langle \zeta _L\psi , \left( H-|\nabla F|^2\right) \zeta _L\psi \rangle&= \langle \psi , e^{2F} \left( |\nabla \chi _R|^2 +2 \chi _R\nabla \chi _R\nabla F \right) \psi \rangle \\&\quad + \langle \psi , \xi ^2 \left( |\nabla \widetilde{\chi }_L|^2 +2 \widetilde{\chi }_L \nabla \widetilde{\chi }_L \nabla F \right) \psi \rangle \, . \end{aligned} \end{aligned}$$

An improvement of Hardy’s inequality outside large balls given in [34] shows

2.46 $$\begin{aligned} \langle \nabla \varphi , \nabla \varphi \rangle \ge \langle \varphi , \left( \left( \frac{d-2}{2}\right) ^2 \frac{1}{|x|^2} +\frac{1}{4|x|^2\ln ^2(|x|)} \right) \varphi \rangle \end{aligned}$$

for any $\varphi \in H^1_0(B_t(0)^c)$ and *t* large enough. Thus

2.47 $$\begin{aligned} \langle \zeta _L\psi , \left( H-|\nabla F|^2\right) \zeta _L\psi \rangle \ge \left\langle \zeta _L\psi , \frac{c}{|x|^2\ln ^2(|x|)} \zeta _L\psi \right\rangle \end{aligned}$$

for some c>0, if the potential *V* is short range and if

2.48 $$\begin{aligned} |\nabla F(x)| \le \frac{d-2}{2|x|} \end{aligned}$$

for all large |*x*|. Note that if *F* is bounded then $\xi =\chi _R e^F$ is bounded and if (2.48) holds then

2.49 $$\begin{aligned} |\langle \psi , \xi ^2 \left( |\nabla \widetilde{\chi }_L|^2 +2 \widetilde{\chi }_L \nabla \widetilde{\chi }_L \nabla F \right) \psi \rangle | \lesssim \Vert \xi \Vert _\infty ^2 \frac{1}{L^2} \int _{L\le |x|\le \alpha L } |\psi (x)|^2\, dx \end{aligned}$$

since $$|\nabla \widetilde{\chi }_L|^2$$ and $$|\nabla \widetilde{\chi }_L\nabla F|\lesssim L^{-2}\textbf{1}_{\{L\le |x|\le \alpha L\}}$$.

Using (2.47) and (2.49) together with Fatou’s lemma in (2.45) one arrives at

2.50 $$\begin{aligned} \begin{aligned} \langle \xi \psi , \frac{c}{|x|^2\ln ^2(|x|)} \xi \psi \rangle&\le \liminf _{L\rightarrow \infty } \langle \zeta _L\psi , \frac{c}{|x|^2\ln ^2(|x|)} \zeta _L\psi \rangle \\&\le \left\langle \psi , e^{2F} \left( |\nabla \chi _R|^2 +2 \chi _R\nabla \chi _R\nabla F \right) \psi \right\rangle \\&\le C_{F,R} \int _{R/2\le |x|\le R}|\psi (x)|^2\, dx \end{aligned} \end{aligned}$$

with the same constant $C_{F,R}$ as in (2.5), since $|\nabla \chi _R|\le \tfrac{4}{R}\textbf{1}_{\{R/2\le |x|\le R\}}$.

Now let $F(x)= \tfrac{d-2}{2}\ln (|x|)$, so that (2.48) holds. In the definition of ξ, replace *F* by $F_\delta$ given in (2.11) for δ>0, i.e., use $\xi =\chi _Re^{F_\delta }$ in the above argument. Then, since $|\nabla F_\delta |\le |\nabla F|$ we see that

$$\begin{aligned} \left\langle \chi _R e^{F_\delta }\psi , \frac{c}{|x|^2\ln ^2(|x|)} \chi _R e^{F_\delta }\psi \right\rangle \le C_{F,R} \int _{R/2\le |x|\le R}|\psi (x)|^2\, dx \, . \end{aligned}$$

In addition, $F_\delta$ converges monotonically to *F* and $e^{F(x)}= |x|^{(d-2)/2}$, so monotone convergence gives

2.51 $$\begin{aligned} \begin{aligned} c\int _{|x|\ge R} \frac{|x|^{d-4}}{\ln ^2(|x|)}|\psi (x)|^2 \, dx&= \lim _{\delta \rightarrow 0} \left\langle \chi _R e^{F_\delta }\psi , \frac{c}{|x|^2\ln ^2(|x|)} \chi _R e^{F_\delta }\psi \right\rangle \\&\le C_R \int _{R/2\le |x|\le R}|\psi (x)|^2\, dx \, . \end{aligned} \end{aligned}$$

with constant $C_R$ given by

$$\begin{aligned} C_R=8\sup _{R/2\le |x|\le R} e^{2F(x)}\left( 2R^{-2}+ R^{-1}|\nabla F(x)| \right) \le 8d R^{d-4} \end{aligned}$$

Since ψ is locally $L^2$, this proves (2.43). □

#### Remark 2.10

In dimensions $2\le d\le 4$ a repulsive tail of the potential *V* of the form

2.52 $$\begin{aligned} V(x)\ge \frac{\omega }{|x|^2} \quad \text {for } |x|\gg 1 \end{aligned}$$

stabilizes zero energy bound states. In this case we can use (2.46) to see that

$$\begin{aligned} \langle \nabla \varphi , \nabla \varphi \rangle +\langle \varphi , V\varphi \rangle \ge \left\langle \varphi , \left( \Big (\Big (\frac{d-2}{2}\Big )^2+\omega \Big ) \frac{1}{|x|^2} +\frac{1}{4|x|^2\ln ^2(|x|)} \right) \varphi \right\rangle \end{aligned}$$

for all $\varphi \in H^1$ with support outside a large enough centered ball in ${\mathbb {R}}^d$. Then the same analysis which yields (2.51), but now with the weight $F(x)= \big (((d-2)/2)^2+\omega \big )^{1/2}\ln |x|$, leads to

2.53 $$\begin{aligned} c\int _{|x|\ge R} \frac{|x|^{\sigma }}{\ln ^2(|x|)}|\psi (x)|^2 \, dx \le C \int _{R/2\le |x|\le R}|\psi (x)|^2\, dx \end{aligned}$$

with $\sigma = 2\big (((d-2)/2)^2+\omega \big )^{1/2}-2$ and some constant *C* depending on *R* and ω. Thus $\psi \in L^2({\mathbb {R}}^d)$ as soon as σ>0 and the last condition is equivalent to

2.54 $$\begin{aligned} \omega > \frac{d(4-d)}{4} \, . \end{aligned}$$

Clearly d(4-d) is positive if d≤3, zero if d=4, and negative if d≥5. So a repulsive part stabilizes zero energy bound state in dimensions d≤4 and in dimensions d≥5 the potential *V* can be purely attractive and still have zero energy eigenstates. The results of [26] show that a repulsive part is needed in dimensions d≤4. Dimension d=4 is critical in the sense that the repulsive part can be weaker in dimension four than for d≤3.

## Local Energy Bound For Helium–Type System in Paraboloidal Regions

**Fig. 1:**
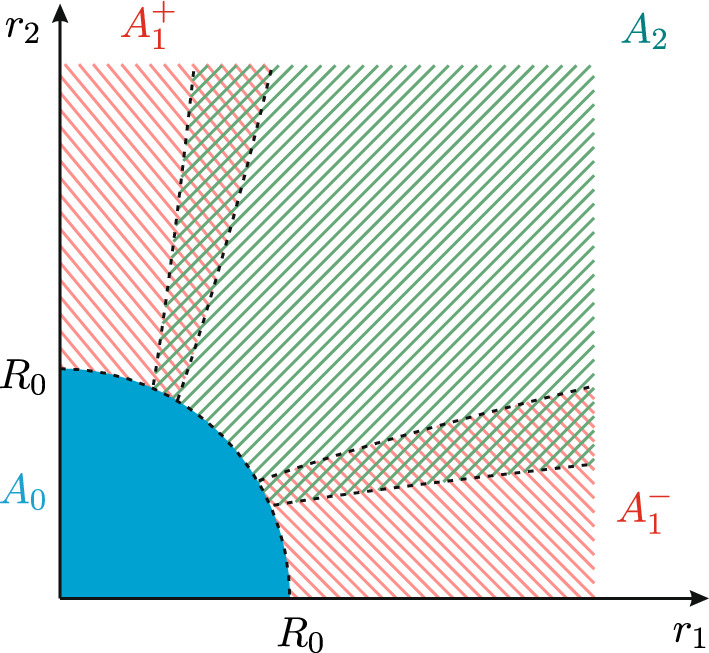
Sketch of the regions $A_0,A_1,A_2$. In $A_0$ both particles are close to nucleus and in $A_2$ both particles are far away. The paraboloidal region $A_1$, which splits into two disjoint components $A_1^-,A_1^+$, corresponds to the situation where one particle is far away while the other stays relatively close to the nucleus. In region $A_1$ we get a local boost in energy, which is important when the system becomes critical

In the following sections, we consider a helium–type atom consisting of an infinitely heavy nucleus at the origin and two indistinguishable electrons. In this section, we prove a local energy bound for these systems, which is the main tool for the proof of the sharp anisotropic upper bounds on the asymptotic behavior of the ground state in later sections. To obtain suitable energy bounds we need appropriate lower bounds on the potential derived in Lemma 3.1. The crucial part of the proof is to split the configuration space into several regions sketched in Fig. 1 where the behavior of the system differs. The bounds on the potential from Lemma 3.1 are then used to derive local energy bounds for the full quantum system in Proposition 3.5

Our local energy bound for the helium–type operator $H_U$ on $L^2({\mathbb {R}}^6)$ is independent of the statistics of the particles and one can easily include the spin. The importance of such a local energy bound was already emphazised by Agmon in his work on non-isotropic upper bounds on the decay of eigenfunctions of multiparticle Schrödinger operators in [1].

We denote by $x_j$ the position of the $j^{\text {th}}$ particle, by $P_j^2$ its kinetic energy, where $P_j=-i\nabla _{x_j}$ is its momentum operator, and j=1,2. The Hamiltonian of this system is given by

3.1 $$\begin{aligned} H_U = P_1^2 + P_2^2 - \frac{1}{|x_1|} - \frac{1}{|x_2|} + \frac{U}{|x_1 - x_2|}\, . \end{aligned}$$

It is well-defined and self-adjoint on the Sobolev space $\mathcal {D}(H_U) = H^2({\mathbb {R}}^6)\subset L^2({\mathbb {R}}^6)$. In the following, it will be very convenient to consider the quadratic form induced by $H_U$ on the Sobolev space $H^1({\mathbb {R}}^6)$. With a slight abuse of notation, we denote this quadratic form by

3.2 $$\begin{aligned} \langle \varphi , H_U\varphi \rangle {:}{=}\langle P_1\varphi , P_1\varphi \rangle + \langle P_2\varphi , P_2\varphi \rangle + \langle \varphi , V_U\varphi \rangle , \end{aligned}$$

where

3.3 $$\begin{aligned} V_U(x)=V_U(x_1,x_2)= - \frac{1}{|x_1|} -\frac{1}{|x_2|}+\frac{U}{|x_1-x_2|}\, . \end{aligned}$$

The proofs of our lower bounds in Sects. 7 and 8 use the sesquilinear version of this quadratic form. Our local energy bound for helium follows from a suitable localization in position space and the IMS localization formula. However, our localization is quite different from the usual approach in many-particle physics, where one localizes into conical regions, see for example [15]. For our derivation of the sharp upper and lower bounds, it turns out to be crucial to use paraboloidal regions.

Recall that for $x=(x_1,x_2)\in {\mathbb {R}}^3\times {\mathbb {R}}^3$ we set $|x|=|(x_1,x_2)|= (|x_1|^2+|x_2|^2)^{1/2}$, $|x|_0 = \min (|x_1|,|x_2|)$, and $|x|_\infty =\max (|x_1|,|x_2|)$. For $R_0 >0$ and $0<\gamma \le 1$ we define the following paraboloidal regions

3.4 $$\begin{aligned} \begin{aligned} \text {Region 0:~}&A_0{:}{=}\{ x\in {\mathbb {R}}^6:\, |x|< 2 R_0 \} \\ \text {Region 1:~}&A_1{:}{=}\{ x\in {\mathbb {R}}^6:\, |x|> R_0 \text { and } |x|_0 < 2 |x|_\infty ^\gamma \} \\ \text {Region 2:~}&A_2{:}{=}\{ x\in {\mathbb {R}}^6:\, |x|> R_0 \text { and } |x|_0 > |x|_\infty ^\gamma \}. \end{aligned} \end{aligned}$$

We do not write explicitly the dependence of these regions on the parameters $R_0$ and γ. The inner region $A_0$ cuts out a ball centered around the nucleus. It is only necessary in order to have a bounded localization error in the IMS localization formula near the nucleus and will be irrelevant for our bounds on eigenstates. All regions above are invariant under permutation of the particles. In order to control the localization error, we will eventually choose the parameter 1/2<γ<1.

In the region $A_0$ both particles are close to the nucleus and in $A_2$ both particles will be far from the nucleus. In these two cases, the local energy bound will be easy. The *tricky region* is $A_1$. At critical coupling $U_c$ this region is critical in the sense that inside this region one particle stays much closer to the nucleus compared to the other particle, which can escape to infinity with *no apparent energy cost*, since the *ionization energy is zero*.

For large $R_0$ the tricky region $A_1$ is the union of two disjoint paraboloid regions

$$\begin{aligned} A_{1}^-&= \{ x\in {\mathbb {R}}^6:\, |x|> R_0 \text { and } |x_2|< 2|x_1|^\gamma \}\, , \\ A_{1}^+&= \{ x\in {\mathbb {R}}^6:\, |x|> R_0 \text { and } |x_1| < 2|x_2|^\gamma \}. \end{aligned}$$

which are “above" and “below" the diagonal $|x_1|=|x_2|$, respectively.

The reason why we introduce these paraboloidal regions is that in these regions, especially in the tricky region $A_1$, the full potential has nice lower bounds which exploit the interaction term but do not involve it explicitly. This is made precise in the following lemma:

### Lemma 3.1

Let $V_U$ be the Coulomb potential

3.5 $$\begin{aligned} V_U(x) = -\frac{1}{|x_1|} - \frac{1}{|x_2|} +\frac{U}{|x_1-x_2|} \end{aligned}$$

on ${\mathbb {R}}^6$. Then for 0<γ<1 we have

3.6 $$\begin{aligned} V_U(x)&\ge -\frac{1}{|x|_0} + \frac{U-1}{|x|_\infty } -\frac{2U }{ |x|_\infty ^{2-\gamma }} \quad \text {for all } x\in A_1\, , \end{aligned}$$

3.7 $$\begin{aligned} V_U(x)&\ge -\frac{1}{|x|_0}- \frac{1}{|x|_\infty } \quad \text {for all } x\in A_2\, . \end{aligned}$$

### Remark 3.2

In the *tricky region* $A_1$, where one particle stays close to the nucleus, and the other one can escape, the bound (3.6) shows that this will have a *local energy cost* of order $(U-1)/|x|_\infty$, which makes the classically forbidden region *stickier*. This is a key input for our a–priori bounds on the decay of ground states at critical coupling. A similar boost was already noted in [15, 23], however, due to the use of conical regions in [15], which is quite common in many–body quantum mechanics, their bound for the Coulomb interaction in the tricky region is worse than our bound in Lemma 3.1: It is *essential to use paraboloidal regions* in order to get the additional positive term $\frac{U-1}{|x|_\infty }$ with the *sharp constant* U-1 in the lower bound for two–particle Coulomb potential in the tricky region. The use of conical regions would not allow us to get sharp upper and lower bound on the anisotropic decay of the ground state, one always incurs an epsilon loss in the leading order terms when using conical regions.

### Proof

The bound on $A_2=\{ |x|> R_0,\, |x|_0 > |x|_\infty ^\gamma \}$ follows by dropping the positive Coulomb repulsion. On $A_1$ we have $|x|_0 < 2|x|_\infty ^\gamma$, and thus

$$\begin{aligned} |x_1-x_2| \le |x_1|+|x_2| \le |x|_\infty +2 |x|_\infty ^\gamma \end{aligned}$$

and

$$\begin{aligned} \frac{U}{|x_1-x_2|} \ge \frac{U}{|x|_\infty + 2|x|_\infty ^\gamma } = \frac{U}{|x|_\infty } - \frac{2U}{|x|_\infty ^{2-\gamma }(1+ 2|x|_\infty ^{\gamma -1})} \, . \end{aligned}$$

This implies (3.6). □

In order to use the bounds from Lemma 3.1, we need to localize the kinetic energy into the different regions $A_0, A_1$, and $A_2$. To construct suitable cut–off functions which localize into these regions, take any two functions $0\le \tilde{u}, \tilde{v}\in \mathcal {C}^\infty ([0,\infty ))$ with $\tilde{u}=1$ and $\tilde{v}=0$ on [0, 1], $\tilde{u}=0$ and $\tilde{v}=1$ on [2,∞), $\tilde{u}>0$ on [0, 7/4], and $\tilde{v}>0$ on [5/4,∞) and set

3.8 $$\begin{aligned} u{:}{=}\frac{\tilde{u}}{(\tilde{u}^2 + \tilde{v}^2)^{1/2}}\, ,\, \quad v{:}{=}\frac{\tilde{v}}{(\tilde{u}^2 + \tilde{v}^2)^{1/2}}\, . \end{aligned}$$

Since $\tilde{u}^2 + \tilde{v}^2\ge c$ for some constant c>0, the functions *u*, *v* are infinitely differentiable. Moreover, by construction, $0\le u,v\le 1$, u=1 and v=0 on [0, 1], u=0 and v=1 on [2,∞), and

3.9 $$\begin{aligned} u^2+v^2 =1 \end{aligned}$$

Note that one can always find *u*, *v* with $\Vert u'\Vert _\infty \le 2$ and $\Vert v'\Vert _\infty \le 2$. Given *u* and *v* we set

3.10 $$\begin{aligned} \begin{aligned} \chi _0(x)&{:}{=}u\Big (|x|/R_0 \Big )\, , \\ \chi _1(x)&{:}{=}v\Big (|x|/R_0 \Big ) u\Big (|x|_0/|x|_\infty ^\gamma \Big )\, , \\ \chi _2(x)&{:}{=}v\Big (|x|/R_0 \Big ) v\Big (|x|_0/|x|_\infty ^\gamma \Big )\, . \end{aligned} \end{aligned}$$

By construction,

3.11 $$\begin{aligned} \sum _{j=0}^2 \chi _j^2 = 1. \end{aligned}$$

In addition, we clearly have $\chi _0\in \mathcal {C}^{\infty }_0({\mathbb {R}}^6)$. This is less obvious for $\chi _1$ and $\chi _2$. For large enough $R_0$ the support of $\chi _1$, which is contained in the tricky region, separates into two disjoint sets (contained in $A_1^\pm$) and on each of these sets $\chi _1$ is infinitely differentiable. So we have $\chi _1\in \mathcal {C}^\infty ({\mathbb {R}}^6)$ for large $R_0$ and a moment of reflection shows that also $\chi _2\in \mathcal {C}^\infty ({\mathbb {R}}^6)$ for all large enough $R_0$. The gradients in ${\mathbb {R}}^6$ of $\chi _0$ and $\chi _1$ are given by

3.12 $$\begin{aligned} \nabla \chi _0(x)&= R_0^{-1} u'\big (|x|/R_0 \big ) \frac{x}{|x|}\, ,\end{aligned}$$

3.13 $$\begin{aligned} \nabla \chi _1(x)&= R_0^{-1} v'\big (|x|/R_0 \big ) u\big (|x|_0/|x|_\infty ^\gamma \big ) \frac{x}{|x|} \nonumber \\&\quad + v\big (|x|/R_0 \big ) u'\big (|x|_0/|x|_\infty ^\gamma \big ) \begin{pmatrix} \big ( \frac{1}{|x_2|^\gamma } \textbf{1}_{\{|x_2|>|x_1|\}} -\gamma \frac{|x_2|}{|x_1|^{\gamma +1}} \textbf{1}_{\{|x_2|<|x_1|\}} \big )\frac{x_1}{|x_1|} \\ \big ( \frac{1}{|x_1|^\gamma } \textbf{1}_{\{|x_1|>|x_2|\}} -\gamma \frac{|x_1|}{|x_2|^{\gamma +1}} \textbf{1}_{\{|x_1|<|x_2|\}} \big )\frac{x_2}{|x_2|} \end{pmatrix}\, . \end{aligned}$$

Thus, abbreviating $s=|x|/R_0$ and $t=|x|_0/|x|_\infty ^\gamma$,

3.14 $$\begin{aligned} |\nabla \chi _0(x)|^2\le R_0^{-2} \big ( u'(s) \big )^2 \le R_0^{-2} \Vert u'\Vert _\infty ^2 \textbf{1}_{\{R_0\le |x|\le 2R_0\}} \end{aligned}$$

and

3.15 $$\begin{aligned} \begin{aligned} |\nabla \chi _1(x)|^2&= R_0^{-2} v'(s)^2 u(t)^2 + 2 R_0^{-1} v'(s) v(s) u'(t) u(t) |x|^{-1} ( t-\gamma t) \\&\quad + v(s)^2 u'(t)^2 |x|_\infty ^{-2\gamma } \Big (1 + \gamma ^2 t^2\Big ) \, . \end{aligned} \end{aligned}$$

For $\nabla \chi _2$ we note that a similar formula as for $\nabla \chi _1$ holds, just with *u* replaced by *v*. Collecting terms we get

$$\begin{aligned} |\nabla&\chi _1(x)|^2 +|\nabla \chi _2(x)|^2 \\&= R_0^{-2} v'(s)^2 \big ( u(t)^2 +v(t)^2\big ) + 2 R_0^{-1} v'(s) v(s) \big ( u'(t) u(t) + v'(t) v(t)\big ) |x|^{-1}(1-\gamma )t \\&\quad + v(s)^2 \big ( u'(t)^2 + v'(t)^2 \big ) |x|_\infty ^{-2\gamma } \Big (1 + \gamma ^2 t^2\Big )\\&= R_0^{-2} v'(s)^2 + v(s)^2 \Big ( u'(t)^2 + v'(t)^2 \Big ) |x|_\infty ^{-2\gamma } \Big (1 + \gamma ^2 t^2\Big ) \end{aligned}$$

where we also used $u^2+v^2=1$ and $2(u'u + v'v) = (u^2+v^2)'=0$. Hence

3.16 $$\begin{aligned} \begin{aligned}&\sum _{j=0}^2 |\nabla \chi _j(x)|^2 = R_0^{-2} \left( v'(|x|/R_0)^2 + u'(|x|/R_0)^2 \right) \\&\quad + v(|x|/R_0)^2 \left( u'\big (|x|_0/|x|_\infty ^\gamma \big )^2 + v'\left( |x|_0/|x|_\infty ^\gamma \right) ^2 \right) \left( \frac{1}{|x|_\infty ^{2\gamma }} + \frac{\gamma ^2|x|_0^2}{|x|_\infty ^{2\gamma +2}}\right) \, . \end{aligned} \end{aligned}$$

This yields the following bound on the localization error for localizing into paraboloidal regions.

### Lemma 3.3

The localization error $\sum _{j=0}^2|\nabla \chi _j|^2$ for the localizing functions $\chi _j$ constructed above, is bounded from above by

3.17 $$\begin{aligned} \begin{aligned} \sum _{j=0}^2 |\nabla \chi _j(x)|^2&\le \Vert (u')^2+ (v')^2\Vert _\infty \left( R_0^{-2} \textbf{1}_{\{R_0\le |x|\le 2R_0\}} + \frac{1+\gamma ^2}{|x|_\infty ^{2\gamma }} \textbf{1}_{\{|x|\ge R_0\}} \right) \end{aligned} \end{aligned}$$

for $$0<\gamma \le 1$$ and $$R_0>0$$.

### Proof

This follows immediately from (3.16) because $0\le u,v\le 1$, $R_0\le |x|\le 2 R_0$ on the support of $v'(|x|/R_0)$, $|x|\ge R_0$ on the support of $v(|x|/R_0)$, and $|x|_0\le |x|_\infty$. □

### Remark 3.4

In order to make $\Vert (u')^2+ (v')^2\Vert _\infty$ small, a convenient choice is to pick

$$\begin{aligned} u(s) = \left\{ \begin{array}{lr} 1, &  \text {for } 0\le s\le 1\\ \cos (\pi (s-1)/2), & \text {for } 1<s\le 2\\ 0, & \text {for } s>2 \end{array} \right. \end{aligned}$$

and $v=\sqrt{1-u^2}$, in which case $\Vert (u')^2 + (v')^2\Vert _\infty = \pi ^2/4$. Making this choice for *u* and *v* does not yield smooth cut-off function, however. Nevertheless, the set on which $\chi _j$ is not differentiable, is a Lebesgue measure zero Lipshitz hypersurface in ${\mathbb {R}}^6$, so Lemma A.1 shows that $\chi _j\in W^{1,\infty }({\mathbb {R}}^6)$ and this is all one needs to use a suitable quadratic form version of the IMS localization formula, see the discussion in Appendix A.

Given a wave function φ in the quadratic form domain of $H_U$, which is the Sobolev space $H^1({\mathbb {R}}^6)$, we can use (3.11) and the fact that $\langle \varphi , H_U\varphi \rangle$ is real together with the IMS localization formula (A.8) to get

3.18 $$\begin{aligned} \begin{aligned} \langle \varphi , H_U\varphi \rangle&= \sum _{j=0}^2 \textrm{Re}\langle \chi _j^2\varphi , H_U\varphi \rangle = \sum _{j=0}^2 \langle \chi _j\varphi , H_U\chi _j\varphi \rangle -\sum _{j=0}^2 \langle \varphi , |\nabla \chi _j|^2\varphi \rangle \, . \end{aligned} \end{aligned}$$

Lemma 3.3 allows us to control the localization error. To bound the local energies $\langle \chi _j\varphi , H_U\chi _j\varphi \rangle$, where each $\chi _j\varphi$ is supported on $A_j$, the following bounds will be useful.

In the following, we take any two functions *u* and *v* as above. Then Lemma 3.3 shows that there exists a constant $C_l= C_l(u,v)<\infty$ such that for any $0<\gamma \le 1$ the localization error is bounded from above by

3.19 $$\begin{aligned} \sum _{j=0}^2|\nabla \chi _j(x)|^2 \le \frac{C_l}{\max (|x|_\infty ^{2\gamma },R_0^{2\gamma })} \text { for all } x\in {\mathbb {R}}^6\, . \end{aligned}$$

For this constant $C_l$ we set

3.20 $$\begin{aligned} W_0(x)&{:}{=}-\frac{1}{2} - \frac{C_l}{R_0^{2\gamma }}\, ,\end{aligned}$$

3.21 $$\begin{aligned} W_1(x)&{:}{=}-\frac{1}{4} + \frac{U-1}{|x|_\infty } -\frac{2U\, }{|x|_\infty ^{2-\gamma }} - \frac{C_l}{|x|_\infty ^{2\gamma }} \, , \end{aligned}$$

and

3.22 $$\begin{aligned} W_2(x)&{:}{=}-\frac{1}{|x|_\infty }- \frac{1}{|x|_0} - \frac{C_l}{|x|_\infty ^{2\gamma }} \, . \end{aligned}$$

Lemma 3.1 and the IMS formula lead to

### Proposition 3.5

(Local energy bound) For large enough $R_0$ and all φ in the quadratic form domain of $H_U$

3.23 $$\begin{aligned} \langle \varphi , H_U\varphi \rangle \ge \sum _{j=0}^2 \langle \chi _j\varphi , W_j \chi _j\varphi \rangle \, . \end{aligned}$$

### Remark 3.6

The localized functions $\chi _j\varphi$, j=0,1,2, have the same permutation symmetry as φ, since the $\chi _j$ are symmetric under permurtation of the particles.

### Proof

Set ${L_{\text {err}}}(x)= \frac{C_l}{\max (|x|_\infty ^{2\gamma },R_0^{2\gamma })}$. From (3.18), Lemma 3.3, and (3.19) we get

$$\begin{aligned} \langle \varphi , H_U\varphi \rangle \ge \sum _{j=0}^2 \langle \chi _j\varphi , H_U\chi _j\varphi \rangle - \langle \varphi , {L_{\text {err}}}\varphi \rangle = \sum _{j=0}^2 \langle \chi _j\varphi , H_U\chi _j\varphi \rangle -\sum _{j=0}^2 \langle \chi _j\varphi , {L_{\text {err}}}\chi _j\varphi \rangle , \end{aligned}$$

since ${L_{\text {err}}}$ is multiplication by a function and $\sum _{j=0}^2\chi _j^2=1$. Thus it is enough to show that

3.24 $$\begin{aligned} \langle \chi _j\varphi , H_U\chi _j\varphi \rangle \ge \langle \chi _j\varphi , \widetilde{W}_j\chi _j\varphi \rangle \end{aligned}$$

for each j=0,1,2, where $\widetilde{W}_j= W_j +{L_{\text {err}}}$. Using that $\chi _2\varphi$ is supported in the region $A_2$, we can use Lemma 3.1 and immediately get (3.24) for j=2 by dropping the kinetic energy term $P_1^2+ P_2^2$ in $\langle \chi _2\varphi , H_U\chi _2\varphi \rangle$.

For j=0 we again drop the Coulomb repulsion term and use

$$\begin{aligned} H_U \ge P_1^2-\frac{1}{|x_1|} +P_2^2- \frac{1}{|x_2|}\ge -1/2 \end{aligned}$$

to get (3.24), since the ground state energy of hydrogen is -1/4 in the atomic units we use.

When j=1, the particles are localized in the tricky region. For large enough $R_0$ the localization function $\chi _1$ is a sum $\chi _1=\chi _{1}^-+\chi _{1}^+$, where $\chi _{1}^\pm$ are smooth and have supports in $A_1^\pm$. In particular, their supports are disjoint for all large $R_0$. Using Lemma 3.1 and dropping $P_1^2$ we get

$$\begin{aligned} \langle \chi _1^-\varphi , H_U\chi _1^-\varphi \rangle&\ge \langle \chi _1^-\varphi , \left( P_2^2-\frac{1}{|x_2| } + \frac{U-1}{|x_1|} -\frac{2U}{|x_1|^{2-\gamma }} \right) \chi _1^-\varphi \rangle \\&\ge \langle \chi _1^-\varphi , \left( -\frac{1}{4} + \frac{U-1}{|x_1|} -\frac{2U}{|x_1|^{2-\gamma }} \right) \chi _1^-\varphi \rangle \\&= \langle \chi _1^-\varphi , \widetilde{W}_1\chi _1^-\varphi \rangle \end{aligned}$$

since $P_2^2- \frac{1}{|x_2|}\ge -1/4$. Similarly, one sees that

$$\begin{aligned} \langle \chi _1^+\varphi , H_U\chi _1^+\varphi \rangle&\ge \langle \chi _1^+\varphi , \widetilde{W}_1\chi _1^+\varphi \rangle \, . \end{aligned}$$

Since the supports of $\chi _1^-$ and $\chi _1^+$ do not overlap when $R_0$ is large enough, we get

$$\begin{aligned} \langle \chi _1\varphi , H_U\chi _1\varphi \rangle&= \langle \chi _1^-\varphi , H_U\chi _1^-\varphi \rangle + \langle \chi _1^+\varphi , H_U\chi _1^+\varphi \rangle \\&\ge \langle \chi _1^-\varphi , \widetilde{W}_1\chi _1^-\varphi \rangle + \langle \chi _1^+\varphi , \widetilde{W}_1\chi _1^+\varphi \rangle = \langle \chi _1\varphi , \widetilde{W}_1\chi _1\varphi \rangle \end{aligned}$$

which is (3.24) for j=1. □

## Isotropic Upper Bounds on the Asymptotic Decay of Bound States

Before we start the proof of the sharp anisotropic upper bound in the next section, we give a proof of a simple isotropic bound. Such an upper bound is already quite useful, since it is *uniform* in the energy E≤-1/4, i.e. up to the bottom of the essential spectrum. It allows for an easy proof of the *existence of a bound state* at critical coupling: The infimum of the spectrum of $H_{U_c}$ is a simple eigenvalue, see Appendix D, even though it is embedded at the edge of the essential spectrum of $H_{U_c}$.

Our main tools are the local energy bounds from Sect. 3 and the quadratic form version of the IMS localization formula from Appendix A. For the simple isotropic upper bound on the asymptotic decay of eigenfunctions of $H_U$ we use the weight function

4.1 $$\begin{aligned} F_1(r)= 2(U-1)^{1/2}r^{1/2} -K r^\kappa \end{aligned}$$

for $1<U\le U_c$, K>0, and 1/6<κ<1/2. The reason why we have to take κ>1/6 in the isotropic upper bound will follow from the proof, in particular, (4.9). Note that the leading order term in $F_1$ is given by $2(U-1)^{1/2}r^{1/2}$, the other term is a *lower order correction* for any K>0 and 1/6<κ<1/2.

When $x=(x_1,x_2)\in {\mathbb {R}}^3\times {\mathbb {R}}^3$, $|x|_\infty =\max (|x_1|,|x_2|)$, we will identify $F_1(x)= F_1(|x|_\infty )$, by a slight abuse of notation. For simplicity of notation, we do not explicitly write the dependence of $F_1$ on its parameters.

Recall that $\psi \in L^2({\mathbb {R}}^6)$ is a bound state of $H_U$ with energy *E*, if it is a weak solution of the eigenvalue equation $H_U \psi =E\psi$. That is, $\psi \in H^1({\mathbb {R}}^6)$ and

4.2 $$\begin{aligned} \langle \varphi , H_U\psi \rangle = E\langle \varphi ,\psi \rangle \end{aligned}$$

as quadratic forms for all $\varphi \in H^1$.

### Theorem 4.1

(Isotropic $L^2$ upper bound near critical coupling) If $\psi _U$ is a bound state of $H_U$ with energy $E_U\le -\frac{1}{4}$ then for any K>0 and 1/6<κ<1/2

4.3 $$\begin{aligned} e^{F_1}\psi _U\in L^2({\mathbb {R}}^6) \end{aligned}$$

Moreover, for normalized $\psi _U$, the function $e^{F_1}\psi _U$ is $L^2$ uniformly in the parameter range $1+\mu \le U\le U_c$ for any small fixed $0<\mu <U_c-1$.

Before we give the proof, we want to explain where the usual strategy fails. Let ψ be a bound state of a Schrödinger operator $H_U$ with energy *E*. Take any bounded, real valued function $\xi \in H^1$. Since *E* is real we can use $\varphi =\xi ^2\psi$ in (4.2) together with the IMS localization formula (A.6) and the local energy bound from Proposition 3.5 to see that

$$\begin{aligned} E\Vert \xi \psi \Vert ^2&= \textrm{Re}(E\langle \xi ^2\psi ,\psi \rangle ) = \textrm{Re}\langle \xi ^2\psi , H_U\psi \rangle = \langle \xi \psi , H_U\xi \psi \rangle -\langle \psi ,|\nabla \xi |^2\psi \rangle \\&\ge \sum _{j=0}^2 \langle \chi _j\xi \psi , W_j \chi _j\xi \psi \rangle -\langle \psi ,|\nabla \xi |^2\psi \rangle \, , \end{aligned}$$

where $\chi _j$, defined in (3.10), are the functions localizing in the regions $A_j$, j=0,1,2.

Now choose $\chi =\chi _R$ to be another smooth cutoff function outside of a centered ball of radius $R> 2R_0$ and make the ansatz $\xi =\chi e^F$ for some bounded function $F\in H^1$. Then

$$\begin{aligned} |\nabla \xi |^2 = \xi ^2|\nabla F|^2 +2 \chi e^{2F}\nabla \chi \cdot \nabla F + e^{2F}|\nabla \chi |^2\, , \end{aligned}$$

and hence

4.4 $$\begin{aligned} \sum _{j=1}^2 \langle \chi _j\chi e^F\psi , (W_j-E-|\nabla F|^2) \chi _j\chi e^F \psi \rangle \le \langle \psi , e^{2F}(2\chi \nabla \chi \nabla F+|\nabla \chi |^2)\psi \rangle \, , \end{aligned}$$

since $\chi _0$ and χ have disjoint supports, the missing j=0 term in the sum on the left hand side above is zero.

The usual strategy for using (4.4) is to assume $W_j-E\ge c>0$. That is, one needs a spectral gap for the operator $H_U-E$ *near infinity* (outside large enough balls), to have a safety distance to the essential spectrum. Under such an assumption, one gets from (4.4)

$$\begin{aligned} \delta c\Vert \chi e^F\psi \Vert ^2 \le \langle \psi , e^{2F}(2\chi \nabla \chi \nabla F+|\nabla \chi |^2)\psi \rangle \le C_{F,\chi }\Vert \psi \Vert ^2\, , \end{aligned}$$

for the exponentially weighted bound state $e^F\psi$ on the support of χ, as long as $|\nabla F|^2\le (1-\delta )c$ for some small δ>0. This condition allows *F* to grow like $\sqrt{(1-\delta )c}|x|$. Of course, one has to remove the requirement that *F* is bounded. This is easy, since the constant $C_{F,\chi }$ depends on *F* and χ only on the support of $\nabla \chi$, which is compact. See the argument in the proof of Theorem 4.1 below, in particular (4.6).

However, the local energy bound $W_1-E_{U_c}= W_1+1/4$ goes to zero at infinity in the *tricky region* $A_1$. Thus c=0, i.e., there is no safety distance to the essential spectrum anymore, when $E_U=-1/4$ or when $E_U$ approaches -1/4 from below as $U\nearrow U_c$, and the above argument does not allow to control $e^F \psi$ anymore. So we have to be more careful.

### Proof of Theorem 4.1

Let $a=(U-1)_+^{1/2}$, 1/6<κ<1/2, K>0, and set

4.5 $$\begin{aligned} G(r){:}{=}2ar^{1/2} - Kr^\kappa /2 \, . \end{aligned}$$

We use *K*/2 instead of *K* in the definition of *G* to have some wiggle room which allows us to absorb error terms later, see (4.12) and (4.13) below. Note that *G*(*r*) is positive when *r* is large enough, so its regularized version

4.6 $$\begin{aligned} G_\delta (r) = \frac{G(r)}{1+\delta G(r) } \end{aligned}$$

is well defined and bounded for all large enough *r* and all δ>0. We also denote $G(x)= G(|x|_\infty )$ and the same for $G_\delta$, with a slight abuse of notation. Note that *G* and $G_\delta$ are continuous on ${\mathbb {R}}^6$ and continuously differentiable on $\{|x_1|\not =|x_2|, |x|>R\}$ for large enough *R*.

Furthermore, let $0\le \chi \le 1$ be a smooth function on [0,∞) with χ(r)=0 for $0\le r\le 1$, χ(r)=1 for r≥2, $|\chi '|\le 2$, and put $\chi _R(x)=\chi (x/R)$. Lemma A.1 shows that $\xi =\chi _R e^{G_\delta }$ is in the Sobolev space $W^{1,\infty }({\mathbb {R}}^6)$ and multiplication with ξ and $\xi ^2$ leaves the quadratic form domain of $H_U$ invariant. Setting $r=|x|_\infty$ one calculates that

4.7 $$\begin{aligned} \nabla G(x) = (ar^{-1/2} -K\kappa r^{\kappa -1}/2) \begin{pmatrix} \frac{x_1}{|x_1|} \textbf{1}_{\{|x_1|>|x_2|\}}\\ \frac{x_2}{|x_2|} \textbf{1}_{\{|x_2|>|x_1|\}} \end{pmatrix}\, , \end{aligned}$$

$$\begin{aligned} \nabla G_\delta =\frac{\nabla G}{(1+\delta G)^{2}} \, . \end{aligned}$$

Thus

$$\begin{aligned} |\nabla G_\delta |^2 \le |\nabla G|^2 = \big (a|x|_\infty ^{-1/2} - K\kappa |x|_\infty ^{\kappa -1}/2 \big )^2\, , \end{aligned}$$

for large enough $|x|_\infty$. Using (4.4) with *F* replaced by $G_\delta$ and taking *R* such that G(x)>0 for all *x* in the support of $\chi _R$ one gets

4.8 $$\begin{aligned} \begin{aligned} \sum _{j=1}^2&\langle \chi _j\chi _R e^{G_\delta }\psi , (W_j-E-|\nabla G|^2) \chi _j\chi _R e^{G_\delta } \psi \rangle \\&\le \langle \psi , e^{2G}(2|\nabla \chi _R||\nabla G|+|\nabla \chi _R|^2)\psi \rangle \le C_R \Vert \psi \Vert ^2 \end{aligned} \end{aligned}$$

where we also used that $G_\delta (x)\le G(x)$ and $|\nabla G_\delta (x)|\le |\nabla G(x)|$ once G(x)>0. The constant $C_R$ is given by

$$\begin{aligned} C_R=4\sup _{R\le s\le 2R} e^{2G(s)}\big (|G'(s)|/R+1/R^2 \big )<\infty , \end{aligned}$$

since $\nabla \chi _R$ is supported on the annulus $R\le |x|\le 2R$ and $|\nabla \chi _R|\le \Vert \chi '\Vert _\infty /R\le 2/R$.

In the following, we will use *C* for a generic constant, which may change from line to line. Recall that $0\le \varepsilon _U=-1/4 -E_U$ is the ionization energy. On $A_2$ we have

$$\begin{aligned} W_2(x)-E_U-|\nabla G(x)|^2 \ge \frac{1}{4} +\varepsilon _U - \frac{1}{|x|_\infty } - \frac{1}{|x|_\infty ^{\gamma }} -\frac{C}{|x|_\infty ^{2\gamma }} - |\nabla G(x)|^2 \ge \frac{1}{8} \end{aligned}$$

for large enough $|x|_\infty$. On $A_1$ we get

4.9 $$\begin{aligned} \begin{aligned} W_1(x)&-E_U-|\nabla G(x)|^2 \ge \varepsilon _U+ \frac{U-1}{|x|_\infty } - \frac{2U}{|x|_\infty ^{2-\gamma }} - \frac{C}{|x|_\infty ^{2\gamma }} -|\nabla G(x)|^2 \\&\ge \varepsilon _U+ aK\kappa |x|_\infty ^{\kappa -3/2} - (K\kappa /2)^2 |x|_\infty ^{2(\kappa -1)} -2U|x|_\infty ^{\gamma -2} - C|x|_\infty ^{-2\gamma } \\&\ge \varepsilon _U+ \frac{3}{4}aK\kappa |x|_\infty ^{\kappa -3/2} -2U|x|_\infty ^{\gamma -2} - C|x|_\infty ^{-2\gamma } \end{aligned} \end{aligned}$$

for all large enough $|x|_\infty$, since κ<1/2, which implies $\kappa -3/2>2(\kappa -1)$. The minimum of $\max (\gamma -2,-2\gamma )$ is attained at γ=2/3. The choice γ=2/3 gives $\gamma -2=-2\gamma = -4/3$, which leads to the lower bound

$$\begin{aligned} W_1(x)-E_U-|\nabla G(x)|^2 \ge \varepsilon _U+ \frac{3}{4}aK\kappa |x|_\infty ^{\kappa -3/2} -(2U+C) |x|_\infty ^{-4/3} \ge \varepsilon _U+ \frac{aK\kappa }{2} |x|_\infty ^{\kappa -3/2} \end{aligned}$$

uniformly in $1+\mu \le U\le U_c\le 2$ for large enough $|x|_\infty$, since κ>1/6. Putting everything together, we see that

4.10 $$\begin{aligned} \sum _{j=1}^2 \chi _R\chi _j (W_j -E_U-|\nabla G|^2 ) \chi _j \chi _R \ge \big (\varepsilon _U+ \frac{aK\kappa }{2} |x|_\infty ^{\kappa -3/2}\big ) \chi _R^2 \ge \frac{\varepsilon _U+ aK}{12} |x|_\infty ^{-4/3} \chi _R^2 \end{aligned}$$

for large enough *R* and all bound state energies $E_U\le -1/4$, i.e., $\varepsilon _U\ge 0$. Using (4.10) in (4.8) we get

4.11 $$\begin{aligned} \langle \chi _Re^{G_\delta }\psi , |x|_\infty ^{-4/3}\chi _Re^{G_\delta }\psi \rangle \le \frac{12 C_R}{\varepsilon _U+ (U-1)_+^{1/2}K} \Vert \psi \Vert ^2 \, \end{aligned}$$

for all δ>0. The right hand side of (4.11) is independent of δ>0, so

$$\begin{aligned} \langle \chi _R e^{G}\psi , |x|_\infty ^{-4/3}\chi _R e^{G}\psi \rangle = \lim _{\varepsilon \rightarrow 0}\langle \chi _Re^{G_\delta }\psi , |x|_\infty ^{-4/3}\chi _Re^{G_\delta }\psi \rangle \le \frac{12 C_R}{\varepsilon _U+(U-1)_+^{1/2}K} \Vert \psi \Vert ^2 \end{aligned}$$

by monotone convergence. Since arbitrary positive multiples of $r^{\kappa }$ control any logarithmic term lnr for large *r*, we have

4.12 $$\begin{aligned} F_1(r)= 2ar^{1/2} - Kr^\kappa \le 2ar^{1/2} - Kr^\kappa /2 - \frac{2}{3}\ln r = G(r) - \frac{2}{3}\ln r \end{aligned}$$

for *r* large. This shows

4.13 $$\begin{aligned} \Vert \chi _R e^{F_1}\psi \Vert ^2\le \langle \chi _R e^{G}\psi , |x|_\infty ^{-4/3}\chi _R e^{G}\psi \rangle \le \tfrac{12 C_R}{\varepsilon _U+(U-1)_+^{1/2}K} \Vert \psi \Vert ^2 \end{aligned}$$

for any $0\le U\le U_c$. It is easy to see that $U\mapsto \varepsilon _U$ is decreasing and the discussion in the introduction shows that $\varepsilon _1>0$. So $\varepsilon _U+(U-1)_+^{1/2}K\gtrsim 1$ uniformly in $0\le U\le U_c$, hence (4.13) proves the theorem. □

Our isotropic upper bound also allows for a simple proof for the existence of a ground state at critical coupling, see Proposition D.1 in the appendix. In the infinite mass approximation, the existence of a bound state at critical coupling had been proven first in [23] with PDE methods and in [15] with variational methods.

### Corollary 4.2

At critical coupling the operator $H_{U_c}$ has a simple eigenvalue at the edge of its essential spectrum. That is, there exists a bound state $\psi _c\in L^2({\mathbb {R}}^6)$ with energy -1/4 of $H_{U_c}$ which is unique up to a phase.

The proof is given in the appendix, where we also gather more information about the ground states $\psi _U$ for $0\le U\le U_c$, see Proposition D.1.

## First Anisotropic Upper Bound

In this section we prove a preliminary version of the anisotropic upper bound from Theorem 1.1. This will be done in two steps: First we prove a $L^2$ version with the help of energy methods and convert this into a pointwise bound in a second step.

Unfortunately, our first anisotropic upper bound given in Proposition 5.3 is *suboptimal in a transition region* where $|x|_\infty ^\gamma \le |x|_0 \le 2|x|_\infty ^\gamma$. So it does not give the sharp anisotropic upper bound we are aiming for. However, it has the *correct asymptotics* well within the *tricky region*, more precisely, when $|x|_0\le |x|_\infty ^\alpha$ for α<1/2, and in the region *near the diagonal*, i.e., where $|x|_0\sim |x|_\infty$ are large. This provides essential a-priori information for the decay of the ground state well within the tricky region and near the diagonal, which is a crucial for the proof of the sharp global anisotropic upper bound in the next section.

Recall that we set $a=a_U{:}{=}(U-1)_+^{1/2}$ and $\varepsilon = \varepsilon _U{:}{=}-\frac{1}{4} - E_U\ge 0$, where $E_U$ is the ground state energy of $H_U$. Moreover recall that

5.1 $$\begin{aligned} \begin{aligned} F_{\varepsilon ,a} = \Big ( \varepsilon +\frac{a^2}{r}\Big )^{1/2}r + \frac{a^2}{\sqrt{\varepsilon }} \big (\ln ( \sqrt{\varepsilon r+a^2} +\sqrt{\varepsilon r} ) -\ln a \big )\, . \end{aligned} \end{aligned}$$

For convenience, we abbreviate

5.2 $$\begin{aligned} F_U=F_{\varepsilon _U,a_U} \end{aligned}$$

and define

5.3 $$\begin{aligned} F_{2,U}(r_1,r_2)= F_U(r_1) - K_1 r_1^{\kappa _1} +\frac{1}{2}\big ( r_2 -K _2 r_2^{\kappa _2} - 2r_1^{\gamma } \big )_+\, . \end{aligned}$$

With a slight abuse of notation, we also use $F_{2,U}(x)=F_{2,U}(|x|_\infty ,|x|_0)$ for $(x_1,x_2)=x\in {\mathbb {R}}^6$.

### Remark 5.1

For $U=U_c$ we have

$$\begin{aligned} F_{2,U_c}(r_1,r_2)= 2(U_c-1)^{1/2}r_1^{1/2} - K_1 r_1^{\kappa _1} +\frac{1}{2}\big ( r_2 -K _2 r_2^{\kappa _2} - 2r_1^{\gamma } \big )_+\, , \end{aligned}$$

see Remark 2.7.

### Remark 5.2

Let us motive the slightly odd looking dependence of $F_{2,U}(r_1,r_2)$ on $r_2$: At first sight, a more natural choice would be

5.4 $$\begin{aligned} F_U(r_1,r_2) = F_U(r_1) + \frac{1}{2} r_2 - K_1 r_1^{\kappa _1} - K_2 r_2^{\kappa _2} \end{aligned}$$

and to use $F_U(x)= F_U(|x|_\infty ,|x|_0)$ as an exponential weight in the energy estimates. For simplicity, let us look at the critical case where $U=U_c$. In this case we have $F_{U_c}(r_1)= 2(U_c-1)^{1/2} r^{1/2}$ and one calculates

$$\begin{aligned} |\nabla F_{U_c}(x)|^2&= \big ( (U_c-1)^{1/2}r_1^{-1/2} - \kappa _1 K_1 r_1^{\kappa _1-1} \big )^2 + \big ( 1/2 - \kappa _2 K_2 r_2^{\kappa _2-1} \big )^2\\&= \frac{(U_c-1)_+}{r_1} +\frac{1}{4} - 2(U_c-1)_+^{1/2}K_1 \kappa _1 r_1^{\kappa _1-3/2} - K_2 \kappa _1 r_2^{\kappa _2-1} \\&\quad + \kappa _1^2K_1^2 r_1^{2(\kappa _1-1)} + \kappa _2^2 K_2^2 r_2^{2(\kappa _2-1)}\, . \end{aligned}$$

The cross terms will allow to control errors, in particular, the localization error and all terms involving $r_1^{2(\kappa _1-1)}$ and $r_2^{2(\kappa _2-1)}$. However, leading order term in $|\nabla F_{U_c}(x)|^2$ now includes the additional constant term 1/4, which needs to be compensated by the ionization energies. In the tricky region, where one particles escapes to infinity, the ionization energy is zero and we only have an additional local boost of the form $\frac{U_c-1}{r_1}$ in the energy estimates from Lemma 3.1 and Proposition 3.5 and *no additional term coming from the second ionization energy*, which is the energy to remove the second particle. The additional constant term 1/4 in $|\nabla F_{U_c}(x)|^2$ cannot be compensated by the *local energy boost in the tricky region*. Thus for the seemingly natural and simpler ansatz (5.4) the gradient of $F_U$ is too big. We need to modify the ansatz to ensure that *F*, hence also its gradient, only depends on $r_1$ inside the tricky region. This is the reason for choosing $\frac{1}{2}\big ( r_2 -K _2 r_2^{\kappa _2} - 2r_1^{\gamma } \big )_+$ as the additional term in (5.3) capturing the decay of the ground state in $r_2=|x|_0$, since it vanished by construction in the tricky region $A_1$ and only becomes positive inside the green shaded region in Fig. 1, where both particles are far from the nucleus and the additional ionization energy of the second particle can compensate the constant term 1/4 in the gradient of the exponential weight.

Eventually we have to choose 1/2<γ<1 in order to be able to control the localization error. The somewhat surprising fact is that the additional term $-2r_1^{\gamma }$ does not mess up the asymptotic behavior of $F_{2,U}(r_1,r_2)$ for large $r_1$ even at criticality, where the leading order term in $F_{2,U}$ only grows proportional to $\sqrt{r_1}$.

### Proposition 5.3

(First global anisotropic $L^2$ upper bound at critical coupling) Choose parameters $K_1,K_2>0$, $1/6<\kappa _1<1/2$ and $1/2-\kappa _1<\kappa _2<1$, as well as $(3-2\kappa _1)/4<\gamma < \min ((\kappa _2+1)/2,\kappa _1+1/2)$. Then the ground state $\psi _U$ of helium-type atoms at coupling *U* has the $L^2$ upper bound

5.5 $$\begin{aligned} e^{F_{2,U}}\psi _U \in L^2({\mathbb {R}}^6) \end{aligned}$$

for all $0\le U\le U_c$ and with $F_{2,U}$ defined in (5.3). Moreover, in the range $0\le U\le U_c-\mu$ for some small μ>0, the bound (5.5) holds with any choice of parameters $0<\kappa _1, \kappa _2<1$ and $1/2<\gamma <(\kappa _2+1)/2$.

### Remark 5.4

The most important part of Proposition 5.3 is of course the statement which holds uniformly in $0\le U\le U_c$. By this we mean that for any choice of ground state $\psi _U$, which is normalized, i.e., $\Vert \psi _U\Vert =1$, we have

$$\begin{aligned} \sup _{0\le U\le U_c} \Vert e^{F_{2,U}}\psi _U\Vert <\infty \, . \end{aligned}$$

A simple calculation shows that $(3-2\kappa _1)/4 < \min ((\kappa _2+1)/2,\kappa _1+1/2)$ is equivalent to $\kappa _1>1/6$ and $\kappa _2>1/2-\kappa _1$. So the range of allowed values for γ in Proposition 5.3 is not empty. Moreover, for any such choice we have $\gamma> (3-2\kappa _1)/4> 1/2$, since $\kappa _1<1/2$. The bound is uniform in $0\le U\le U_c$ and the implicit constant depends only on the parameters $K_1,K_2>0$, $1/6<\kappa _1<1/2$ and $1/2-\kappa _1<\kappa _2<1$.

### Proof

As in the proof of the isotropic bound, we use a modified version of $F_{2,U}$,

5.6 $$\begin{aligned} G_2(r_1,r_2){:}{=}F_U(r_1) - K_1 r_1^{\kappa _1}/2 +\frac{1}{2}\big ( r_2 -K _2 r_2^{\kappa _2} - 2r_1^{\gamma } \big )_+ \end{aligned}$$

replacing $K_1$ by $K_1/2$. In the remainder, we will use $\partial _1=\partial _{r_1}$, respectively, $\partial _2=\partial _{r_2}$ and also freely abbreviate $a=a_U$.

We again use the smooth cut–off functions $\chi _R$, which projects outside of large balls of radius *R* centered at zero and whose gradient $\nabla \chi _R$ is supported on the annulus $R\le |x|\le 2R$ and bounded by $|\nabla \chi _R|\lesssim R^{-1}$.

We also put $G_2(x)=G_2(|x|_\infty ,|x|_0)$ as a function on ${\mathbb {R}}^6$, with a slight abuse of notation. Note that $G_2(x)\ge 0$ for all *x* in the support of $\chi _R$ as long as *R* is large enough, so we can regularize $G_2$ by using

5.7 $$\begin{aligned} G_{2,\delta } = \frac{G_2}{1+\delta G_2} \end{aligned}$$

which is then well defined on the support of $\chi _R$ and bounded for all ε>0. Clearly $G_{2,\delta }$ is continuous on and differentiable on $\{|x|_0 -K _2 |x|_0^{\kappa _2} - 2|x|_\infty ^{\gamma }> 0\}\cap \{ |x|>R_0\}$ and $\{|x|_0 -K _2 |x|_0^{\kappa _2} - 2|x|_\infty ^{\gamma } < 0\}\cap \{ |x|>R_0\}$, for all large enough $R_0$. Up to the smooth zero Lebesgue measure surface $\{|x|_0 -K _2 |x|_0^{\kappa _2} - 2|x|_\infty ^{\gamma } = 0, |x|>R_0\}$ these two sets cover $\{|x|>R_0\}$. Hence Lemma A.1 shows that the exponential weight $\xi =\chi _R e^{G_{2,\delta }}$ has, for all large enough *R*, a bounded weak derivative on ${\mathbb {R}}^6$, which is almost everywhere given by its classical gradient and we can use ξ in the IMS localization formula.

As in the proof of the isotropic upper bound, see (4.4), one deduces from the IMS localization formula and the local energy bound from Proposition 3.5 that

5.8 $$\begin{aligned}&\sum _{j=1}^2 \langle \chi _j\chi _R e^{G_{2,\delta }}\psi , (W_j - E_U-|\nabla G_2|^2) \chi _j\chi _R e^{G_{2,\delta }} \psi \rangle \nonumber \\&\quad \le \langle \psi , e^{2G_2}(2|\nabla \chi _R||\nabla G_2|+|\nabla \chi _R|^2)\psi \rangle \, , \end{aligned}$$

by choosing $\varphi = (\chi _R e^{G_{2,\delta }})^2\psi$ as a test function in the quadratic form version of the eigenvalue equation. $W_1$ and $W_2$ are given in (3.21) and (3.22). Similar to the derivation of (4.8), we used $|\nabla G_{2,\delta }(x)|\le |\nabla G_2(x)|$ and $G_{2,\delta }(x)\le G_2(x)$ when $|x|_\infty$ is large and there is no j=0 term in (5.8) since $\chi _0\chi _R=0$ for all $R>2R_0$.

Recall that $\chi _1$ localizes into the tricky region $A_1=\{|x|_0< 2|x|_\infty ^\gamma , |x|>R_0\}$. Clearly $G_2(x)= 2a |x|_\infty ^{1/2} - K_1 |x|_\infty ^{\kappa _1}/2$ for $x\in A_1$. Thus, setting $r_1=|x|_\infty$,

5.9 $$\begin{aligned} \nabla G_2(x) = \begin{pmatrix} \partial _1 G_2 \frac{x_1}{|x_1|}\textbf{1}_{\{ |x_1|>|x_2|\}}\\ \partial _1 G_2 \frac{x_2}{|x_2|}\textbf{1}_{\{ |x_2|>|x_1|\}} \end{pmatrix} = ((\varepsilon +a^2/r_1)^{1/2} -K_1\kappa _1 r_1^{\kappa _1-1}/2 ) \begin{pmatrix} \frac{x_1}{|x_1|}\textbf{1}_{\{ |x_1|>|x_2|\}}\\ \frac{x_2}{|x_2|}\textbf{1}_{\{ |x_2|>|x_1|\}} \end{pmatrix} \end{aligned}$$

for all $x\in A_1$. Using $-E_U= \frac{1}{4}+\varepsilon$ we have on the support of $\chi _1$

$$\begin{aligned} W_1(x)-&E_U-|\nabla G_2|^2 \\&\ge \varepsilon + a^2 r_1^{-1} - 2U r_1^{\gamma -2} - ((\varepsilon +a^2/r_1)^{1/2}- K_1\kappa _1r_1^{\kappa _1-1}/2)^2 - C r_1^{-2\gamma } \\&= K_1\kappa _1 r_1^{\kappa _1-1}\big (2(\varepsilon +a^2/r_1)^{1/2}- K_1\kappa _1r_1^{\kappa _1-1}/2)\big )/2 - 2U r_1^{\gamma -2} - C r_1^{-2\gamma } \, . \end{aligned}$$

If $0\le U\le U_c-\mu$ then $\varepsilon =\varepsilon _U\ge c>0$ for some constant depending on μ>0. In this case we get

$$\begin{aligned} W_1(x)-&E_U-|\nabla G_2|^2 \\&\ge K_1\kappa _1 r_1^{\kappa _1-1}\big (2 c^{1/2}- K_1\kappa _1r_1^{\kappa _1-1}/2)\big )/2 - 2U r_1^{\gamma -2} - C r_1^{-2\gamma } \\&\gtrsim r_1^{\kappa _1-1} - r_1^{\gamma -2} - r_1^{-2\gamma } \gtrsim r_1^{\kappa _1-1} \ge r_1^{-1} \end{aligned}$$

for large enough $r_1$, i.e., large enough $R_0$, as long as $\kappa _1-1 > \max (\gamma -2,-2\gamma )$, which is equivalent to $(1-\kappa _1)/2<\gamma <1+\kappa _1$.

In the range $0\le U\le U_c$, we note that when $r_1\ge 1$ we have $\varepsilon + a^2/r_1= \varepsilon _U+ a_U^2/r_1\ge (\varepsilon _U+(U-1)_+)/r_1\ge c/r_1$, with $c=\inf _{0\le U\le U_c} \big ( \varepsilon _U+ (U-1)_+\big )>0$. So uniformly in $0\le U\le U_c$ we have

$$\begin{aligned} W_1(x)- E_U-|\nabla G_2|^2&\ge K_1\kappa _1 r_1^{\kappa _1-1}\big (2(c/r_1)^{1/2}- K_1\kappa _1r_1^{\kappa _1-1}/2)\big )/2 - 2U r_1^{\gamma -2} - C r_1^{-2\gamma } \\&\gtrsim r_1^{\kappa _1-3/2} - r_1^{\gamma -2} - r_1^{-2\gamma } \gtrsim r_1^{\kappa _1-3/2} \ge r_1^{-4/3} \end{aligned}$$

for large enough $r_1$, i.e., large enough $R_0$, as long as $\kappa _1-3/2 > \max (\gamma -2,-2\gamma )$, which is equivalent to $(3-2\kappa _1)/4<\gamma <\kappa _1+1/2$. We also used that $\kappa _1>1/6$ in the last bound.

For the energy bound on the support of $\chi _2\subset A_2$, we split $A_2$ into the two regions $A_2^- =\{|x_1|^\gamma<|x_2|< |x_1|, |x_1|>R_0 \}$ and $A_2^+= \{|x_2|^\gamma<|x_1|< |x_2|, |x_2|>R_0 \}$ which cover $A_2$ up to a null set within the diagonal $\{ |x_1|=|x_2| \}$. It is enough to provide a lower bound for $W_2-E_U- |\nabla G_2|^2$ on $A_2^-$, the same bound will then also hold on $A_2^+$, by symmetry, Since null sets are irrelevant such a bound will then hold on the support of $\chi _2$.

For the same reason, we can also disregard the null set $\{ |x_2|-K_2 |x_2|^{\kappa _2}- 2|x_1|^\gamma = 0\}\cap A_2^-$, on which the classical gradient of $G_{2}$ does not exist.

On $\{ |x_2|-K_2 |x_2|^{\kappa _2}- 2|x_1|^\gamma < 0\}\cap A_2^-$ we again have $G_2(x)= 2a|x_1|^{1/2}-K_1|x_1|^{\kappa _1}$. Thus

$$\begin{aligned} W_2(x) -&E_U - |\nabla G_2(x)|^2\\&\ge \frac{1}{4} +\varepsilon - r_1^{-1} -r_1^{-\gamma } - ((\varepsilon +a^2/r_1)^{1/2}- K_1\kappa _1r_1^{\kappa _1-1}/2)^2 - C r_1^{-2\gamma } \\&\ge \frac{1}{4} - (1+a^2)r_1^{-1} - r_1^{-\gamma } -(K_1\kappa _1 r_1^{\kappa _1-1}/2)^2 \ge \frac{1}{8} \end{aligned}$$

on this set and all large enough R>0.

For $x\in \{ |x_2|-K_2 |x_2|^{\kappa _2}- 2|x_1|^\gamma > 0\} \cap A_2^-$, we have

5.10 $$\begin{aligned} G_2(x) = F_U(r_1) - K_1 r_1^{\kappa _1}/2 +\frac{1}{2} \big ( r_2 -K _2 r_2^{\kappa _2} - 2r_1^{\gamma } \big ) \end{aligned}$$

with $r_1=|x_1|$ and $r_2=|x_2|$. Hence

5.11 $$\begin{aligned} \nabla G_2(x) = \begin{pmatrix} \partial _1 G_2 \frac{x_1}{|x_1|}\\ \partial _2 G_2 \frac{x_2}{|x_2|} \end{pmatrix} = \begin{pmatrix} \big ((\varepsilon _U+a^2/r_1)^{1/2} -K_1\kappa _1 r_1^{\kappa _1-1}/2 - \gamma r_1^{\gamma -1}\big ) \frac{x_1}{|x_1|}\\ \frac{1}{2}\big (1 -K_2\kappa _2 r_2^{\kappa _2-1}\big ) \frac{x_2}{|x_2|} \end{pmatrix} \end{aligned}$$

which implies

$$\begin{aligned} W_2&(x) -E_U - |\nabla G_2(x)|^2 \ge W_2(x) +\frac{1}{4}+ \varepsilon - |\nabla G_2(x)|^2 \\&= \frac{1}{4} -\frac{1}{4}\left( 1-K_2\kappa _2r_2^{\kappa _2-1}\right) ^2- r_1^{-1} -r_2^{-1} \\&~~ +\varepsilon - \left( (\varepsilon +a^2/r_1)^{1/2}- K_1\kappa _1r_1^{\kappa _1-1}/2 -\gamma r_1^{\gamma -1}\right) ^2 - C r_1^{-2\gamma } \\&\ge \frac{1}{4}K_2\kappa _2 r_2^{\kappa _2-1}\left( 2-K_2\kappa _2 r_2^{\kappa _2-1} \right) - r_1^{-1} - r_2^{-1} - \frac{a^2}{r_1} \\&+ \left( \frac{\varepsilon +a^2}{r_1} \right) ^{1/2} \left( K_1\kappa _1 r_1^{\kappa _1-1} +2\gamma r_1^{\gamma -1}\right) - \left( K_1\kappa _1 r_1^{\kappa _1-1}/2 +\gamma r_1^{\gamma -1}\right) ^2 - C r_1^{-2\gamma } \, . \end{aligned}$$

Using again $\varepsilon +a^2= \varepsilon _U+(U-1)_+\gtrsim 1$ uniformly in $0\le U\le U_c$ and also that $\gamma>1/2> \kappa _1$, so terms such as $r_1^{\gamma -1}$ control $r_1^{\kappa _1-1}$ for large $r_1$, we arrive at the lower bound

$$\begin{aligned} W_2&(x) -E_U - |\nabla G_2(x)|^2 \\&\ge \frac{1}{4}K_2\kappa _2 r_2^{\kappa _2-1}\left( 2-K_2\kappa _2 r_2^{\kappa _2-1} \right) - r_1^{-1} - r_2^{-1} - \frac{a^2}{r_1} \\&+ \left( \frac{\varepsilon +a^2}{r_1} \right) ^{1/2} \left( K_1\kappa _1 r_1^{\kappa _1-1} +2\gamma r_1^{\gamma -1}\right) - \left( K_1\kappa _1 r_1^{\kappa _1-1/2} +\gamma r_1^{\gamma -1}\right) ^2 - C r_1^{-2\gamma } \\&\gtrsim r_2^{\kappa _2-1} - r_2^{-1} + r_1^{\gamma -3/2} -r_1^{2(\gamma -1)}- r_1^{-2\gamma } \ge r_2^{\kappa _2-1} - r_2^{-1} + r_1^{\gamma -3/2} -r_2^{2(\gamma -1)}- r_1^{-2\gamma } \\&\ge r_2^{\kappa _2-1} \ge r_1^{\kappa _2-1}\ge r_1^{-1} \end{aligned}$$

for all $r_1^\gamma < r_2\le r_1$, and large enough $r_1$, as long as $0<\kappa _2<1$, $\kappa _2-1 > 2(\gamma -1)$, and $\gamma -3/2>-2\gamma$, which is equivalent to $1/2<\gamma <(\kappa _2+1)/2$.

It is straightforward to check that $1/2<\gamma <(\kappa _2+1)/2$ and $(3-2\kappa _1)/4<\gamma <\kappa _1+1/2$ is equivalent to $(3-2\kappa _1)/4<\gamma < \min ((\kappa _2+1)/2,\kappa _1+1/2)$, since $(3-2\kappa _1)/4>1/2$ when $\kappa _1<1/2$. This is the condition on the parameter γ for the range $0\le U\le U_c$.

One also easily checks that $(1-\kappa _1)/2<\gamma <1+\kappa _1$ and $1/2<\gamma <(\kappa _2+1)/2$ is equivalent to $1/2<\gamma <(\kappa _2+1)/2$ for $0<\kappa _1,\kappa _2<1$. This is the condition on the parameter γ when *U* stays away from the critical coupling.

Collecting the lower bounds on $\frac{1}{4}+ W_j- |\nabla G_2|^2$ on the supports of $\chi _1$ and $\chi _2$ and plugging this into (5.8), one arrives at

$$\begin{aligned} \langle \chi _R e^{G_{2,\delta }}\psi , |x|_\infty ^{-4/3} \chi _R e^{G_{2,\delta }}\psi \rangle \le C\Vert \psi \Vert ^2 \, . \end{aligned}$$

The constant *C* is uniform in ε>0, since $G_2$ is bounded on the support of $\nabla \chi _R$ for any fixed R>0. Hence we can again use the monotone convergence theorem to conclude

$$\begin{aligned} \langle \chi _R e^{G_2}\psi , |x|_\infty ^{-4/3 } \chi _R e^{G_2}\psi \rangle =\lim _{\varepsilon \rightarrow 0} \langle \chi _R e^{G_{2,\delta }}\psi , |x|_\infty ^{-4/3} \chi _R e^{G_{2,\delta }}\psi \rangle \le C\Vert \psi \Vert ^2 \, \end{aligned}$$

which proves the theorem since $G_2(x)- \tfrac{2}{3}\ln |x|_\infty \ge F_2(x)$ for all large $|x|_\infty$. □

### Corollary 5.5

(Pointwise version of Proposition 5.3) Given parameters $K_1,K_2>0$, $1/6<\kappa _1<1/2$, and $1/2-\kappa _1<\kappa _2<1$, as well as $(3-2\kappa _1)/4<\gamma < \min ((\kappa _2+1)/2,\kappa _1+1/2)$, there exists a constant C>0, depending only on the above parameters such that for any normalized ground state $\psi _U$ of the helium–type system Hamiltonian $H_U$ we have

5.12 $$\begin{aligned} \psi _U(x) \le C\exp [-F_{2,U}(|x|_\infty ,|x|_0)] \quad \text {for all } x\in {\mathbb {R}}^6\, , \end{aligned}$$

uniformly in $0\le U\le U_c$, with $F_2$ defined in (5.3).

Moreover, in the subcritical range $0\le U\le U_c-\mu$ for some small μ>0, the bound (5.12) holds with any choice of parameters $0<\kappa _1, \kappa _2<1$ and $1/2<\gamma <(\kappa _2+1)/2$.

### Remark 5.6

As discussed in the introduction, we expect the ground state to have an asymptotic anisotropic decay given by

5.13 $$\begin{aligned} \psi _c(x) \le C \exp \big (-2(U_c-1)|x|_\infty ^{1/2} - \frac{1}{2}|x|_0\big )\, . \end{aligned}$$

up to some lower order corrections. The bound provided by Corollary 5.5 does not quite achieve to prove this. If $|x|_\infty ^\gamma \le |x|_0 \le 2 |x|_\infty ^\gamma$ we expect the bound state to decay at least as fast as

5.14 $$\begin{aligned} \psi _c(x) \le C \exp \big (-2(U_c-1)|x|_\infty ^{1/2} - \frac{1}{2}|x|_\infty ^\gamma \big )\, . \end{aligned}$$

and since 1/2<γ<1, the second term now dominates. However, the upper bound from Corollary 5.5 only shows that

5.15 $$\begin{aligned} \psi (x)\le C\exp \big (- 2(U_c-1)_+^{1/2}|x|_\infty ^{1/2} +K|x|_\infty ^{\kappa _1}\big ) \end{aligned}$$

in the region $|x|_0\le 2|x|_\infty ^\gamma$. This is the reason why we have to refine the upper bound from Corollary 5.5 in the next section. Nevertheless, the bound from Corollary 5.5 provides essential a–priori information for this refinement, see the proof of Lemma 6.2.

### Proof

Proposition D.1 shows that $H_{U}$ has a unique ground state $\psi _U$ for any $0\le U\le U_c$, which up to a phase can be chosen positive. The subsolution estimate of Trudinger [45] in the version of Aizenman and Simon [4], see also [39, Theorem C.1.3] shows that for any r>0 and $x\in {\mathbb {R}}^6$

5.16 $$\begin{aligned} |\psi _U(x)|\le C_1 \int _{|x-y|\le r} |\psi _U(y)|\, d y \end{aligned}$$

since the Coulomb potential is in the Kato class. The constant $C_1$ depends only on *r* and the Kato norm of the potential, see, e.g., [39, Section C.1]. Thus, with $|B^d_1|$ the volume of the unit ball in ${\mathbb {R}}^d$,

5.17 $$\begin{aligned} \begin{aligned} e^{F_{2,U}(x)} |\psi _U(x)|&\le C_1|B^d_1|^{1/2}\left( \int _{|x-y|\le 1} e^{2F_{2,U}(x)} |\psi _U(y)|^2\, d y \right) ^{1/2} \\&\le C_1|B^d_1|^{1/2} \sup _{|x-y|\le 1} e^{F_{2,U}(x)- F_{2,U}(y)} \Vert e^{F_{2,U}}\psi _U\Vert \end{aligned} \end{aligned}$$

for all $x\in {\mathbb {R}}^6$. One easily checks that for all $|r_1|\gg 1\,\text { and }\,|r_1-s_1|, |r_2-s_2|\le 1$

$$\begin{aligned}&F_{2,U}(r_1,r_2) - F_{2,U}(s_1,s_2) \\  &\lesssim |r_1^{1/2}-s_1^{1/2}| + |r_1^{\kappa _1}-s_1^{\kappa _1}| + |r_1-s_1| + |r_2^{\kappa _2}-s_2^{\kappa _2}| + |r_1^\gamma - s_1^\gamma | \\  &\lesssim t^{-1/2} + t^{\kappa _1-1} +1 +\min \{2, u^{\kappa _2-1}\} + t^{\gamma -1} \end{aligned}$$

with $$t=\max (r_1,s_1)$$, $$u=\max (r_2,s_2)$$. Hence

5.18 $$\begin{aligned} C_2{:}{=}\sup _{x\in {\mathbb {R}}^6} \sup _{|x-y|\le 1}|F_{2,U}(x) -F_{2,U}(y)| <\infty \, . \end{aligned}$$

So (5.17) gives the pointwise exponential upper bound

5.19 $$\begin{aligned} |\psi _U(x)| \le C C_1 C_2 |B^d_1|^{1/2}e^{-F_{2,U}(x)} \quad \text {for all } x\in {\mathbb {R}}^6 \end{aligned}$$

for the ground state $\psi _U$, where *C* is the constant from the $L^2$ upper bound of Proposition 5.3. This proves Corollary 5.5. □

## Global Anisotropic Upper Bound

In this section we give the proof of Theorem 1.1 and the proof of the upper bound from Theorem 1.5. Recall that the function $F_+$ is given by

6.1 $$\begin{aligned} F_+(r_1,r_2)= 2(U_c-1)^{1/2}r_1^{1/2} - K_1 r_1^{\kappa _1} +\frac{1}{2} r_2- K_2 r_2^{\kappa _2} \end{aligned}$$

and the function $F^U_+$ is given by

6.2 $$\begin{aligned} F^U_+(r_1,r_2)= F_U(r_1) - K_1 r_1^{\kappa _1} +\frac{1}{2} r_2- K_2 r_2^{\kappa _2} \end{aligned}$$

for $r_1,r_2\ge 0$ with $F_U$ defined in (1.9). We also set $F_+(x)=F_+(|x|_\infty ,|x|_0)$ and the same for $F^U_+$. We choose $\psi _U$ to be the unique positive ground state of $H_U$. We want to use the comparison principle from Theorem B.3 to show that there exist a constant 0<C<∞, depending only on the parameters $\kappa _1,\kappa _2, K_1, K_2$ in the definition of $F^U_+$ such that

6.3 $$\begin{aligned} \psi _U(x) \le C\exp (-F^U_+(|x|_\infty ,|x|_0)) \end{aligned}$$

for all $x\in {\mathbb {R}}^6$ and all $0\le U\le U_c$. Since $\psi _U$ is bounded by Proposition D.1 and $\exp (-F^U_+)$ is bounded away from zero on compact sets uniformly in *U*, it is enough to assume that $|x|_\infty > R$ for some large R>0. In this section, we abbreviate

6.4 $$\begin{aligned} f_U{:}{=}\exp (-F^U_+) \end{aligned}$$

and note that, similarly as for $F^U_+$, we will not explicitly write the dependence of $f_U$ on the other parameters except *U*, for simplicity of notation.

### Remark 6.1

Note that $F^U_+$, hence also *f*, is twice continuously differentiable for all $0<|x|_0<|x|_\infty$. However, it is not twice differentiable on any neighborhood of the diagonal $|x_2|=|x_1|$, since the gradient of $F^U_+$ has a jump discontinuity across the diagonal. Nevertheless, we could use Agmon’s quadratic form version of the comparison principle, see Theorem B.3, as long as one could control certain boundary terms on the diagonal $|x_1|=|x_2|$, which appear from integration by parts. However, as the proof of the *global lower bound* in Sect. 8, in particular, the proof of Lemma 8.6, will show, the boundary terms have the *wrong sign and cannot be discarded*. This is why we cannot apply the comparison theorem directly. Instead, one has to apply the comparison theorem separately on $\{R^\alpha<|x_2|<|x_1|\}$ and $\{R^\alpha<|x_1|<|x_2|\}$, for large enough *R*. This forces us to have *very precise a-priori information* about the asymptotic behavior of $\psi _U$ at infinity near the diagonal $|x|_0=|x|_\infty$, since we need the a–priori information that $\psi _U(x)\lesssim f_U(x)$ near the diagonal $|x_1|=|x_2|$. Fortunately this is exactly what Corollary 5.5 provides. We summarize the necessary a-priori information about the asymptotic decay of $\psi _c$ near the diagonal and within the tricky region in the following

### Lemma 6.2

Given any $K_1,K_2>0$, $1/6<\kappa _1<1/2$, $0<\alpha <\kappa _1$, and $3/4-\kappa _1/2< \kappa _2<1$, there exist a constant 0<C<∞ such that

6.5 $$\begin{aligned} \psi _U(x)&\le C f_U(x) \end{aligned}$$

on the sets $\{|x|_0\le |x|_\infty ^\alpha , |x|_\infty \ge R\}$ and $\{|x|_\infty -1\le |x|_0\le |x|_\infty , |x|_\infty \ge R\}$ for all large enough R>0 and all $0\le U\le U_c$.

Moreover, in the subcritical range $0\le U\le U_c-\mu$ for some small μ>0, for any choice of parameters $0<\kappa _1, \kappa _2<1$, $\max (\kappa _1,\kappa _2)>1/2$, $0<\alpha <\min (\kappa _1,1/2)$, and $K_1,K_2>0$, there exist R>0 such that the bound (6.5) holds on the above sets and the constant *C* is again independent of $0\le U\le U_c-\mu$.

### Remark 6.3

**Fig. 2:**
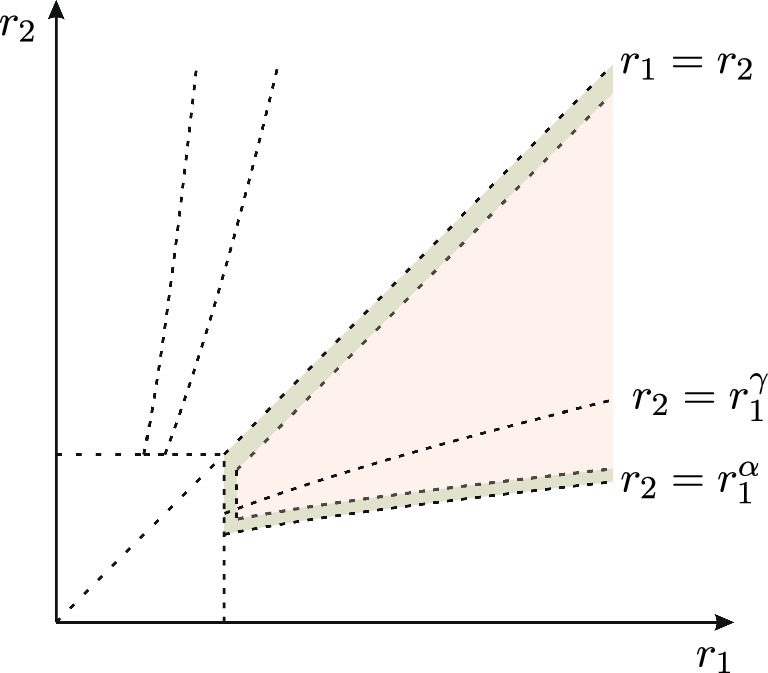
The intermediate region $B_R^-$ and its boundary layer (colored in green). Lemma 6.2, which is heavily based on Corollary 5.5, provides sharp a–priori upper bounds on the ground state in the part of the boundary layer of $B_R^-$ near the diagonal $r_2=r_1$ and deep within the tricky region when $r_2=r_1^\alpha$

The first part of Lemma 6.2 provides an upper bound on the ground state $\psi _U$ in the green shaded region near the diagonal $r_1=r_2$ and deep within the tricky region near $r_2= r_1^\alpha$ in Fig. 2 up to the critical case when $U=U_c$.

The second part allows for a wider range of parameters $\kappa _1,\kappa _2$ but works only for subcritical couplings *U* which stay away from $U_c$.

### Proof

The conditions $1/6<\kappa _1$ and $3/4-\kappa _1/2 < \kappa _2$ are equivalent to

$$\begin{aligned} (3-2\kappa _1)/4<\min (\kappa _2,\kappa _1+1/2)\, . \end{aligned}$$

Since $\kappa _2<1$, we also have $\kappa _2<(\kappa _2+1)/2$, so any γ with $(3-2\kappa _1)/4<\gamma <\min (\kappa _2,\kappa _1+1/2)$ fulfills the condition of Corollary 5.5 in the whole range $0\le U\le U_c$.

In the subcritical range where *U* stays away from $U_c$, we choose $1/2<\gamma <\max (\kappa _1,\kappa _2)$. Note that for this choice the conditions of Corollary 5.5 for the subcritical case are also satisfied since $\kappa _2<(\kappa _2+1)/2$.

Given the parameters $\kappa _1,\kappa _2,\gamma$ and $K_1,K_2>0$ we use the exponential weight $F_{2,U}$ from Corollary 5.5, but with $K_1$ replaced by $K_1/2$, that is, with a slight abuse of notation, we use

$$\begin{aligned} F_{2,U}(r_1,r_2) = F_U(r_1) -K_1r_1^{\kappa _1}/2 +\frac{1}{2} \left( r_2-K_2r_2^{\kappa _2} - 2r_1^\gamma \right) _+ \, . \end{aligned}$$

Due to $\alpha<1/2<\gamma$, we have $F_{2,U}(r_1,r_2) = F_U(r_1) - \frac{K_1}{2} r_1^{\kappa _1}$ when $r_2\le r_1^\alpha$ and $r_1$ is large. In particular,

$$\begin{aligned} F_{2,U}(r_1,r_2)&= F_U(r_1) - \frac{K_1}{2} r_1^{\kappa _1} \ge F_U(r_1) - K_1 r_1^{\kappa _1} +\frac{1}{2}r_2 - K_2 r_2^{\kappa _2} + \frac{K_1}{2} r_1^{\kappa _1} -\frac{1}{2} r_2 \\&\ge F^U_+(r_1,r_2) + \frac{K_1}{2} r_1^{\kappa _1} -\frac{1}{2} r_1^\alpha >F^U_+(r_1,r_2) \end{aligned}$$

if $r_2\le r_1^\alpha$ and $r_1$ is large enough since $\alpha <\kappa _1$. Similarly,

$$\begin{aligned} F_{2,U}(r_1,r_2)&= F_U(r_1) - \frac{K_1}{2} r_1^{\kappa _1} + \frac{1}{2}r_2 -\frac{K_2}{2}r_2^{\kappa _2} -r_1^\gamma \\&\ge F^U_+(r_1,r_2) + \frac{K_1}{2}r_1^{\kappa _1} + \frac{K_2}{2}(r_1-1)^{\kappa _2} -r_1^\gamma > F^U_+(r_1,r_2), \end{aligned}$$

when $r_1-1<r_2\le r_1$ and $r_1$ is large, since $\gamma <\kappa _2$ in the critical case and $\gamma <\max (\kappa _1,\kappa _2)$ in the subcritical case. From Corollary 5.5 we get, the pointwise upper bound

6.6 $$\begin{aligned} \psi _U(x) \le C \exp [-F_{2,U}(x)] \le C \exp [-F^U_+(x)] = Cf_U(x) \end{aligned}$$

in the regions $|x|_0\le |x|_\infty ^\alpha$, respectively, $|x|_\infty -1<|x|_0\le |x|_\infty$, when $|x|_\infty$ is large enough. □

The next Lemma shows that $f_U$ is a supersolution on a set “sandwiched between” the sets $\{|x|_0\le |x|_\infty ^\alpha , |x|_\infty \ge R\}$ and $\{|x|_\infty -1\le |x|_0\le |x|_\infty , |x|_\infty \ge R\}$.

### Lemma 6.4

Let $0<\kappa _1<1/2<\kappa _2<1$, $K_1,K_2>0$, and 0<α<1. Then the function $f_U= \exp (-F^U_+)$, with $F^U_+$ given in (6.1), is a classical supersolution of $H_U$ at energy $E_U$ on the set

6.7 $$\begin{aligned} B_R=\{ |x|_\infty ^\alpha -1<|x|_0<|x|_\infty , |x|_\infty >R \} \end{aligned}$$

for all large enough R>0; that is,

6.8 $$\begin{aligned} (H_U-E_U)f_U \ge 0 \text { pointwise in } B_R\, . \end{aligned}$$

In particular, $f_U$ is a supersolution in the quadratic form sense a la Agmon: for large enough *R* and all $0\le \varphi \in \mathcal {C}^\infty _0(B_R)$ we have the quadratic form inequality

6.9 $$\begin{aligned} \langle \varphi , (H_U-E_U) f_U \rangle \ge 0 \, . \end{aligned}$$

Moreover, in the subcritical range, where $0\le U\le U_c-\mu$ for some small μ>0, for any choice of parameters $0<\kappa _1, \kappa _2<1$, and 0<α<1, there exists R>0 such that the function $f_U$ is a classical supersolution in the set $B_R$.

### Proof

In order to be able to control the errors, it will be important that both particles are far from the nucleus in $B_R$. For large enough R>0, the set $B_R$ is the disjoint union of $B_R^-{:}{=}\{ |x_1|^\alpha -1<|x_2|<|x_1|, |x_1|>R \}$, the part of $B_R$ “below the diagonal” $|x_2|=|x_1|$, and the part $B_R^+$ above the diagonal, defined similarly as $B_R^-$ but with $x_1$ and $x_2$ interchanged. By symmetry of $f_U$, which is clear from the symmetry of $F^U_+$, it is enough to show that $f_U$ is a classical supersolution of $H_U$ at energy $E_U$ on $B_R^-$.

Let ∇ be the gradient and Δ the Laplacian on ${\mathbb {R}}^6$. We abbreviate $F=F^U_+$ and $f=f_U$. Since they are twice differentiable on $B_R^-$ we clearly have

$$\begin{aligned} -\Delta f= f\big [-|\nabla F|^2 +\Delta F\big ] \end{aligned}$$

on $B_R^-$. Also recall that $\varepsilon = -\frac{1}{4}-E_U\ge 0$ is the ionization energy (which is zero when $U=U_c$) and we also abbreviate $a=(U-1)_+^{1/2}$, so $F_U'(r_1) = (\varepsilon +a^2/r_1)^{1/2}$. Moreover, we have $f(x)= \exp (-F(r_1,r_2))$ with $r_1=|x_1|$ and $r_2=|x_2|$ on $B_R^-$, so one easily calculates

6.10 $$\begin{aligned} \nabla F(x)&= \begin{pmatrix} \partial _1 F\frac{x_1}{|x_1|} \\ \partial _2 F\frac{x_2}{|x_2|} \end{pmatrix} = \begin{pmatrix} \big ( F_U'(r_1) - K_1\kappa _1 r_1^{\kappa _1-1}\big )\frac{x_1}{|x_1|} \\ \big (\frac{1}{2} -K_2\kappa _2r_2^{\kappa _2-1}\big )\frac{x_2}{|x_2|} \end{pmatrix} \quad \text {on } B_R^- \end{aligned}$$

and

6.11 $$\begin{aligned} \Delta F(x)&= \partial _1^2F +\partial _1 F \frac{2}{|x_1|} + \partial _2^2F +\partial _2 F \frac{2}{|x_2|} \quad \text {on } B_R^- \, . \end{aligned}$$

Thus

$$\begin{aligned} -\Delta f = f\left[ -|\partial _1 F|^2 - |\partial _2F|^2 + \partial _1^2F +\partial _1 F \frac{2}{|x_1|} + \partial _2^2F +\partial _2 F \frac{2}{|x_2|} \right] \, \end{aligned}$$

on $B_R^-$, and hence we also have that

$$\begin{aligned} (H_U-E_U)f&= f\Big [ \varepsilon -|\partial _1 F|^2 +\frac{1}{4} - |\partial _2F|^2 + \partial _1^2F +\partial _1 F \frac{2}{|x_1|} + \partial _2^2F +\partial _2 F \frac{2}{|x_2|} \\&\quad - \frac{1}{|x_1|} - \frac{1}{|x_2|}+ \frac{U}{|x_1-x_2|} \Big ]\\&\ge f\Big [ \varepsilon -|\partial _1 F|^2 +\frac{1}{4}- |\partial _2F|^2 + \partial _1^2F +\partial _2 F \frac{2}{r_2} - \frac{1}{r_1} - \frac{1}{r_2} \Big ]\, , \end{aligned}$$

where we dropped the positive terms $\partial _2^2F$, $U/|x_1-x_2|$, and also $\partial _1F\frac{2}{r_1}$, which is positive for large enough $r_1$. Furthermore, using $F_U'(r_1)= (\varepsilon +a^2/r_1)^{1/2}\ge (\varepsilon +a^2)^{1/2}r_1^{-1/2} \ge c r_1^{-1/2}$ with the constant $c^2= \inf _{0\le U\le U_c}(\varepsilon _U+a_U^2)>0$ we have

$$\begin{aligned} \varepsilon -|\partial _1F|^2&= \varepsilon -\left( F_U'(r_1)-K_1\kappa _1 r_1^{\kappa _1-1}\right) ^2 \\&= -a^2 r_1^{-1} +2K_1\kappa _1 F_U'(r_1) r_1^{\kappa _1-1} -(K_1\kappa _1 r_1^{\kappa _1-1})^2 \\&\gtrsim - r_1^{-1} + r_1^{\kappa _1-3/2} - r_1^{2(\kappa _1-1)} \gtrsim -r_1^{-1} + r_1^{\kappa _1-3/2} \, , \\ \end{aligned}$$

since $$\kappa _1-3/2>2(\kappa _1-1)$$. Moreover,

$$\begin{aligned} \frac{1}{4}- |\partial _2F|^2&= K_2\kappa _2r_2^{\kappa _2-1}(1 -K_2\kappa _2r_2^{\kappa _2-1}) \sim r_2^{\kappa _2-1}\, ,\\ \partial _1^2 F&= F_U''(r_1) + K_1\kappa _1(1-\kappa _1) r_1^{\kappa _1-2} = -\frac{a^2}{r_1^2}\left( \varepsilon +a^2/r_1 \right) ^{-1/2} + K_1\kappa _1(1-\kappa _1) r_1^{\kappa _1-2} \\&\ge -\frac{a^2}{r_1^2}\frac{r_1^{1/2}}{c} + K_1\kappa _1(1-\kappa _1) r_1^{\kappa _1-2} \gtrsim -r_1^{-3/2} \, ,\\ \partial _2F\frac{2}{r_2} - r_2^{-1}&= ( \frac{1}{2} - K_2\kappa _2 r_2^{\kappa _2-1})\frac{2}{r_2} -r_2^{-1} \sim - r_2^{\kappa _2-2} \end{aligned}$$

for all large enough $r_1,r_2$. So we get

$$\begin{aligned} (H_U-E_U)f&\gtrsim f\Big [ -r_1^{-1} + r_1^{\kappa _1-3/2} +r_2^{\kappa _2-1} -r_1^{-3/2} -r_2^{\kappa _2-2} \Big ] \gtrsim f\Big [ -r_1^{-1} +r_2^{\kappa _2-1} \Big ]\\&\ge f\Big [ -r_1^{-1} +r_1^{\kappa _2-1} \Big ] \gtrsim f \, r_1^{\kappa _2-1} > 0 \, , \end{aligned}$$

for all $x\in B_R^-$ and large enough *R*. The second inequality holds since $r_1^{\kappa _1-3/2}>r_1^{-3/2}$ for large $r_1$, the third because $r_2^{\kappa _2-1}\ge r_1^{\kappa _2-1}$ and the fourth, because $r_1^{\kappa _2-1} - r_1^{-1}= r_1^{\kappa _2-1}(1 -r_1^{-\kappa _2})\gtrsim r_1^{\kappa _2-1}$. This proves Lemma 6.4 uniformly in $0\le U\le U_c$.

In the subcritical case, when $0\le U\le U_c-\mu$ for some fixed small μ>0, we have the bound

$$\begin{aligned} \varepsilon -|\partial _1F|^2&\gtrsim - r_1^{-1} + r_1^{\kappa _1-1} - r_1^{2(\kappa _1-1)} \gtrsim r_1^{\kappa _1-1}\, , \end{aligned}$$

uniformly in $0\le U\le U_c-\mu$ and therefore

$$\begin{aligned} (H_U-E_U)f&\gtrsim f\Big [ r_1^{\kappa _1-1} +r_2^{\kappa _2-1} -r_1^{-3/2} -r_2^{\kappa _2-2} \Big ] > 0 \, , \end{aligned}$$

since $0<\kappa _1, \kappa _2<1$, $r_1\ge R$, $r_2\ge R^\alpha -1$ and *R* is large enough, which proves the second claim. □

Now we come to the proof of the global upper bound.

### Theorem 6.5

(Sharp upper bound, arbitrary coupling) For any choice of parameters $K_1,K_2>0$, $1/6<\kappa _1<1/2$, and $(3-2\kappa _1)/4<\kappa _2<1$ there exist constants $C_+$ depending only on $\kappa _1,\kappa _2, K_1,$ and $K_2$, such that for the unique positive choice of the ground state of the helium-type operator $H_U$ the pointwise bound

6.12 $$\begin{aligned} \psi _U(x)\le C_+ \exp \left( -F^U_+(|x|_\infty ,|x|_0) \right) \end{aligned}$$

holds uniformly in $0\le U\le U_c$.

For the subcritical case, where for fixed small μ>0 the repulsion parameter *U* is allowed to vary uniformly in $0\le U\le U_c-\mu$ assume that $0<\kappa _1,\kappa _2<1$, $\max (\kappa _1,\kappa _2)>1/2$, and $K_1,K_2>0$. Then there exist a constant $\widetilde{C}_+$, depending only on $\kappa _1,\kappa _2, K_1, K_2$, and also μ, such that the upper bound

6.13 $$\begin{aligned} \psi _U(x)\le \widetilde{C}_+ \exp \left( -F^U_+(|x|_\infty ,|x|_0) \right) \end{aligned}$$

holds for all $0\le U\le U_c -\mu$.

### Remark 6.6

Note that Theorem 1.1 is a special case of Theorem 6.5 for $U=U_c$.

### Proof of Theorem 6.5:

Since $\psi _U$ is bounded uniformly in $0\le U\le U_c$, see Proposition D.1, and $f_U=\exp (-F^U_+)$ is bounded away from zero on compact sets uniformly in $0\le U\le U_c$, we clearly have $\psi _U(x)\le Cf_U(x)=C\exp (-F^U_+(x))$ for all $|x|_\infty \le R$ for some constant *C*, which might depend on *R* and the parameters but not on *U*.

So it is enough to show there exists some constant *C* such that $\psi _U(x)\le C f_U(x)$ on $\{|x|_\infty >R\}$ for some R>0. By symmetry, it is enough to prove that there exists a constant *C* such that

6.14 $$\begin{aligned} \psi _U(x) \le Cf_U(x)= C\exp (-F^U_+(x)) \end{aligned}$$

for all $\{ |x_2|\le |x_1|, |x_1|>R \}$ and large enough R>0.

Fix any $0<\alpha <\min (\kappa _1,1/2)$. Due to the assumptions on the parameters $\kappa _1,\kappa _2,K_1,K_2$ in Theorem 6.5 the assumptions of Lemma 6.2 are satisfied with this choice of α. Hence the bound (6.14) holds for some constant *C* on the sets $B_{1,R}{:}{=}\{ |x_2|\le |x_1|^\alpha , |x_1|>R \}$ and $B_{2,R}{:}{=}\{ |x_1|-1\le |x_2|\le |x_1|, |x_1|>R \}$, both in the critical and subcritical case.

So we only have to prove the same bound on the intermediate region $B_R^-=\{ |x_1|^\alpha -1<|x_2|<|x_1|, |x_1|>R \}$. Consider

$$\begin{aligned} \widetilde{\partial } B_R^1&{:}{=}\{ |x_1|-1\le |x_2|< |x_1|, |x_1|>R+1\} \\ \widetilde{\partial } B_R^2&{:}{=}\{ |x_1|^\alpha -1 < |x_2| \le |x_1|^\alpha , |x_1|>R+1\} \\ \end{aligned}$$

and

$$\begin{aligned} \widetilde{\partial } B_R^0&{:}{=}\{ |x_1|^\alpha -1< |x_2|< |x_1|, R< |x_1|\le R+1\} \, . \end{aligned}$$

Then $\widetilde{\partial } B_R= \cup _{j=0}^2 \widetilde{\partial } B_R^j$ is a boundary layer of $B_R^-$ in the sense of Definition B.2.

Moreover, as already mentioned above, we know from Lemma 6.2 that (6.14) holds on the parts $\widetilde{\partial } B_R^1$ and $\widetilde{\partial } B_R^2$ when *R* is large enough. Since $f_U= \exp (-F^U_+)$ is bounded away from zero on any compact set and the closure of $\widetilde{\partial } B_R^0$ is bounded, we see that $f_U$ is bounded away from zero on $\widetilde{\partial } B_R^0$. Using that $\psi _U$ is bounded, shows that the bound (6.14) also holds on $\widetilde{\partial } B_R^0$ for some constant *C*. Thus, enlarging the constant if necessary, we see that the upper bound (6.14) holds on the boundary layer $\widetilde{\partial } B_R$ for some constant *C*. This constant only depends on the constant from Lemma 6.2, on uniform bounds on $\psi _U$ from Proposition D.1, and lower bounds on $f_U$ on compact sets, which are independent of *U*.

To finish the argument, we note that $f_U$ is a classical supersolution, hence also in the quadratic form sense, of $H_U$ at energy $E_U$ on $B_R^-$ by Lemma 6.4 (for the precise notion see the discussion in Appendix Appendix B). Moreover, $\psi _U\in H^1({\mathbb {R}}^6)$ is a solution, hence also a subsolution, in the quadratic form sense a la Agmon on $B_R^-$. The subharmonic comparison principle given in Theorem B.3 then yields the upper bound (6.14) on all of $B_R^-$. This proves the upper bound from Theorem 1.5 in the critical and subcritical case. □

### Remark 6.7

It is very convenient to have a quadratic form version of the subharmonic comparison principle, since it allows us to directly work with weak eigenfunctions, which are only in $H^1({\mathbb {R}}^6)$ and not in $H^2({\mathbb {R}}^6)$. To the best of our knowledge, the quadratic form version of the subharmonic comparison principle goes back to a beautiful paper by Agmon [2]. Theorem B.3 in the appendix is a slight extension of Agmon’s original result. Agmon works on open sets which are neighborhoods of infinity, i.e., complements of compacts sets, while we have to work on unbounded sets, which are not necessarily neighborhoods of infinity.

## Lower Bound in the Tricky Region

### Theorem 7.1

(Lower pointwise bound in the tricky region) Let $H_U$ be the helium–type Schrödinger operator given in (1.1) and let $\psi _U \in L^2({\mathbb {R}}^6)$ be the positive ground state of $H_{U}$ for $0\le U\le U_c$. Then for any 1/6<κ<1/2 and any $0<\gamma <\kappa +1/2$ and any K>0

7.1 $$\begin{aligned} \psi _U (x)\gtrsim \exp \Big (-F_U(|x|_\infty )-K|x|_\infty ^\kappa -\frac{1}{2}|x|_0\Big ) \quad \text {for all } |x|_0\le |x|_\infty ^\gamma \end{aligned}$$

with $F_U$ defined in (1.9). Again we use $|x|_0=\min \{|x_1|,|x_2|\}$, $|x|_\infty =\max \{|x_1|,|x_2|\}$. The implicit constant in the above bound depends on the parameters $\kappa , \gamma$, and *K*, but not on *U* in the range $0\le U\le U_c$.

Moreover, in the subcritical case, i.e., when *U* stays away from $U_c$, the lower bound (7.1) holds for any fixed small μ uniformly in $0\le U\le U_c-\mu$ and any 0<κ<1 and 0<γ<1.

### Remark 7.2

Theorem 7.1 provides a lower bound for the ground state when one particle escapes while the other stays close to the nucleus. This is an important a-priori bound for our proof of the global lower bound.

Since the leading order behavior of $F_U(r)$ switches from linear to $r^{1/2}$ at $U=U_c$, we need the restriction κ<1/2 in the first case, so that $r^{\kappa }$ is a lower order correction compared to the leading order term $F_U(r)$, uniformly in $0\le U\le U_c$.

When *U* stays away from $U_c$, the leading order term $F_U(r)$ is always linear in *r*, which allows for a larger range for κ and γ.

### Proof

For 0<γ<1 define $\widetilde{A}=\{|x|_0\le |x| _\infty ^\gamma \}$. For large enough *R*, the regions $\widetilde{A}_R^-=\{ |x_2|\le |x_1|^\gamma , |x_1|> R \}$ and $\widetilde{A}_R^+=\{ |x_1|\le |x_2|^\gamma , |x_2|> R \}$ are disjoint. By symmetry, a lower bound of the form (7.1) on the set $\widetilde{A}_R^-$, for some R>0 implies the same bound on $\widetilde{A}_R^+$. The set $\widetilde{A}\setminus (\widetilde{A}_R^-\cup \widetilde{A}_R^+) =\{|x|_0\le |x|_\infty ^\gamma \le R^\gamma \}$ is a compact subset of ${\mathbb {R}}^6$. Clearly the right hand side of (7.1) bounded above on compact sets and, because of Proposition D.1, $\psi _U$ is bounded away from zero on compact sets. Hence there is some constant C>0 with

$$\begin{aligned} \psi _U(x)\ge C\exp \Big (-F_U(|x|_\infty )-K|x|_\infty ^\kappa -\frac{1}{2}|x|_0\Big ) \quad \text {for all } x\in \widetilde{A}\setminus (\widetilde{A}_R^-\cup \widetilde{A}_R^+)\, . \end{aligned}$$

Thus, shrinking the involved constant, if necessary, one sees that the lower bound (7.1) holds on $\widetilde{A}$ iff it holds on $\widetilde{A}_R^-$ for some R>0.

For technical reasons, we need to work on the slightly larger set

$$\begin{aligned} A_{R}^-{:}{=}\{ |x_2|< 2|x_1|^\gamma , |x_1|\ge R \}. \end{aligned}$$

which is just the (lower) tricky region from Sect. 3. Recall that $a=(U-1)_+^{1/2}$ and $\varepsilon = -\frac{1}{4}-E_U$ is the ionization energy. $F_U$, defined in (1.9), depends on these two parameters. Finally we define

7.2 $$\begin{aligned} \begin{aligned} F_{3,U}(r_1,r_2)&= F_U(r_1) +K r_1^\kappa -\tfrac{1}{2}r_2\quad \text { for } r_1,r_2\ge 0\, ,\\ \Phi (s)&{:}{=}{\left\{ \begin{array}{ll} 1\,, & \quad 0\le s\le 1\\ \cos \left( \frac{\pi }{2}(s-1)\right) \,, & \quad 1<s<2\\ 0\,, & \quad 2\le s\\ \end{array}\right. } \, \\ \chi (x)&{:}{=}\Phi \Big ( \frac{|x_2|}{|x_1|^\gamma } \Big ) \quad \text {for } x=(x_1,x_2)\in A_{R}^- \end{aligned} \end{aligned}$$

and

7.3 $$\begin{aligned} g_U(x){:}{=}\chi (x) \exp (-F_{3,U}(|x_1|, |x_2|)) \quad \text {for } x=(x_1,x_2)\in A_{R}^-\, . \end{aligned}$$

We will suppress the dependence of $F_{3,U}$ and $g_U$ on the other parameters, except on *U*, for simplicity of notation. Note that χ(x)=1 on $\widetilde{A}_R^-$, so the bound (7.1) will hold on $\widetilde{A}_R^-$, hence also on $\widetilde{A}$, once we show that $\psi _U\ge C g_U$ on $A_{R}^-$ for some R>0 and some constant C>0, which can depend on all the parameters, but not on *U*.

The boundary $\partial A_{R}^-$ is the union of the unbounded part $\partial A_{R,1}^-=\{ |x_2|= 2|x_1|^\gamma , |x_1|\ge R \}$ and the compact set $\partial A_{R,2}^-=\{|x_1|=R, |x_2|\le 2 R^\gamma \}$. We clearly have $\psi _U(x)>0 =g_U(x)$ for all $x\in \partial A_{R,1}^-$. By continuity of $\psi _U$ and $g_U$, there exists an open neighborhood $O_1$ of $\partial A_{R,1}^-$ such that $\psi _U(x)> g_U(x)$ for all $x\in O_1$. Hence $\psi _U \ge g_U$ on $O_1\cap A_R^-$.

Moreover, $\psi _U>0$ is bounded from below by a positive constant on compact subsets of ${\mathbb {R}}^6$, uniformly in $0\le U\le U_c$, see Proposition D.1 and $g_U\ge 0$ is continuous and depends continuously on the parameters. Thus, since $\partial A_{R,2}^-$ is compact, there exists a constant C>0 such that $\psi _U\ge 2Cg_U>Cg_U$ on $\partial A_{R,2}^-$ for all $0\le U\le U_c$. By continuity, there exist an open neighborhood $O_2$ of $\partial A_{R,2}^-$ such that $\psi _U>Cg_U$ on $O_2$, hence $\psi _U> Cg_U$ on $O_2\cap A_R^-$. Thus

$$\begin{aligned} \psi _U\ge \min (C,1)g_U \text {~~ on the boundary layer } (O_1\cup O_2)\cap A_R^- \, . \end{aligned}$$

Now assume that 1/6<κ<1/2 and $(3-2\kappa )/4<\gamma <\kappa +1/2$. In Lemma we show that under these conditions there exists R>0 such that $g_U$ is a subsolution of $H_U$ at energy $E_U$ in $A_{R}^-$, for all $0\le U \le U_c$, in the quadratic form sense a la Agmon. Moreover, it is easy to see that $g_U\in L^2({\mathbb {R}}^6)$. Hence we can apply Theorem B.3 to conclude that $\psi _U\ge C g_U$ on all of $A_{R}^-$, hence on all of $\{|x|_0\le |x|_\infty ^\gamma \}$.

It remains to get rid of the lower bound on γ. Note that the sets $\{|x|_0\le |x|_\infty ^\gamma \}$ are increasing in 0<γ<1, i.e., once the bound (7.1) holds for some $(3-2\kappa )/4<\gamma <\kappa +1/2$ it holds for all $0<\gamma \le \kappa +1/2$. This finishes the proof of Theorem 7.1. □

It remains to prove that for suitable choices of the parameters, the function $g_U$ defined in (7.3) is a classical subsolution in the tricky region.

### Lemma 7.3

Let κ>1/6 and $(3-2\kappa )/4<\gamma <\min (\kappa +1/2,1)$. Then there exists R>0 such that the function $g_U$ defined in (7.3) is a classical subsolution of $H_U$ at energy $E_U= -1/4-\varepsilon _U\le -1/4$ on the set $A_{R}=\{ |x|_0<2|x|_\infty ^\gamma , |x|_\infty >R \}$, that is,

7.4 $$\begin{aligned} (H_U-E_U) g_U \le 0 \quad \text {pointwise in } A_{R} \, \end{aligned}$$

for any $0\le U\le U_c$. In particular, it is a subsolution in the quadratic form sense a la Agmon: for large enough *R* and all $0\le \varphi \in \mathcal {C}^\infty _0(A_{R})$ we have the quadratic form inequality

7.5 $$\begin{aligned} \langle \varphi , (H_U-E_U) g_U \rangle \le 0 \, . \end{aligned}$$

Moreover, in the subcritical case, i.e., when *U* stays away from $U_c$, the same holds for any fixed small μ uniformly in $0\le U\le U_c-\mu$ and any κ>0 and $\max ((1-\kappa )/2,0)<\gamma <1$.

### Remark 7.4

It is easy to see that $(3-2\kappa )/4<\kappa +1/2$ is equivalent to κ>1/6. So the set of allowed values for γ is not empty iff κ>1/6.

### Proof

The bound (7.5) immediately follows from (7.4) by integration by parts, since φ has compact support inside $A_{R}$ and is non–negative on $A_{R}$. Moreover, since $A_{R}$ splits into the regions $A_{R}^-=\{ |x_2|<2|x_1|^\gamma , |x_1|>R \}$ and $A_{R}^+=\{ |x_1|<2|x_2|^\gamma , |x_2|>R \}$ which are disjoint for large enough *R*, it is enough to prove (7.4) on $A_{R}^-$, by symmetry.

In the remainder of the proof, we will work only on $A_{R}^-$, abbreviate $F=F_{3,U}$ and $g=g_U$, and also identify the function *F* with $F(|x_1|,|x_2|)$, for simplicity of notation.

Clearly, *F* is twice continuously differentiable on $A_{R}^-$. Moreover, Φ is twice differentiable on [0, 2) and one easily checks that $g\in H^1_0(A_{R}^-)\cap H^2(A_{R}^-)$. Since $\nabla g = \nabla \chi e^{-F}-\chi \nabla F e^{-F}$, we have

$$\begin{aligned} -\Delta g = e^{-F}\big [ \chi \Delta F -\chi |\nabla F|^2 -\Delta \chi +2\nabla \chi \nabla F\big ] \, . \end{aligned}$$

From the definition of *F* and χ, setting $r_1=|x_1|$, $r_2=|x_2|$, and recalling $\partial _1=\partial _{r_1}$, $\partial _2=\partial _{r_2}$, one calculates that

$$\begin{aligned} \nabla F(x)&= \begin{pmatrix} \partial _1 F\frac{x_1}{|x_1|} \\ \partial _2 F\frac{x_2}{|x_2|} \end{pmatrix} = \begin{pmatrix} \big ( F_U'(r_1)+K\kappa r_1^{\kappa -1}\big )\frac{x_1}{|x_1|} \\ \frac{1}{2}\frac{x_2}{|x_2|} \end{pmatrix}\, \\ \end{aligned}$$

with $F_U'(r_1) =(\varepsilon +a^2/r_1)^{1/2}$ and $\varepsilon =1/4-E_U\ge 0$ and $a=(U-1)_+^{1/2}$. Moreover,

$$\begin{aligned} \nabla \chi (x)&= \Phi '\Big (\frac{r_2}{r_1^\gamma } \Big ) \begin{pmatrix} -\gamma \frac{r_2}{r_1^{\gamma +1}}\frac{x_1}{|x_1|} \\ \frac{1}{r_1^\gamma }\frac{x_2}{|x_2|} \end{pmatrix}\, . \end{aligned}$$

Thus

$$\begin{aligned} \nabla \chi (x) \nabla F(x)&= \Phi '\Big (\frac{r_2}{r_1^{\gamma }}\Big ) \, \left[ \partial _2 F r_1^{-\gamma } - \gamma \partial _1 F \,r_2 r_1^{-\gamma -1} \right] \end{aligned}$$

and

$$\begin{aligned} \Delta \chi (x)&= \Phi ''\Big (\frac{r_2}{r_1^{\gamma }}\Big )\, \left( \gamma ^2 r_2^2 r_1^{-2\gamma -2} + r_1^{-2\gamma } \right) +\Phi '\Big (\frac{r_2}{r_1^{\gamma }}\Big ) \left( 2r_1^{-\gamma } r_2^{-1}- \gamma (1-\gamma ) r_2r_1^{-\gamma -2} \right) \, , \end{aligned}$$

so

7.6 $$\begin{aligned} \begin{aligned} 2\nabla \chi (x) \nabla F(x)&-\Delta \chi (x) \\&= - \Phi ''\Big (\frac{r_2}{r_1^{\gamma }}\Big )\, \left( \gamma ^2 r_2^2 r_1^{-2\gamma -2} + r_1^{-2\gamma } \right) \\&\quad + \Phi '\Big (\frac{r_2}{r_1^{\gamma }}\Big ) \, r_1^{-\gamma }\left[ 2\partial _2 F - 2\gamma \partial _1 F\, r_2 r_1^{-1} +\gamma (1-\gamma ) r_2r_1^{-2} - 2 r_2^{-1} \right] \, . \end{aligned} \end{aligned}$$

Note that $$\Phi '\le 0$$ and since the support of $\Phi^{\prime }$ is contained in the interval [1, 2], we have $$r_1^\gamma \le r_2\le 2r_1^\gamma $$ for the second term on the right hand side above. Thus we get the lower bound

$$\begin{aligned} 2\partial _2 F - 2\gamma \partial _1 F\, r_2 r_1^{-1} +\gamma (1-\gamma ) r_2r_1^{-2} - 2 r_2^{-1} \ge 2\partial _2 F - 4\gamma \partial _1 F\, r_1^{\gamma -1} - 2 r_2^{-\gamma } \gtrsim 1 \end{aligned}$$

since $2\partial _2F = 1$ and $\partial _1F$ is bounded at infinity uniformly in $0\le U\le U_c$.

So we can again use $\Phi '\le 0$ to drop the second term on the right hand side of (7.6) to arrive at

$$\begin{aligned}&2\nabla \chi (x) \nabla F(x) -\Delta \chi (x) \le - \Phi ''\Big (\frac{r_2}{r_1^{\gamma }}\Big )\, \left( \gamma ^2 r_2^2 r_1^{-2\gamma -2} + r_1^{-2\gamma } \right) \\&\quad \le \frac{\pi ^2}{4}\chi (x) \, \left( 4\gamma ^2 r_1^{-2} + r_1^{-2\gamma } \right) \end{aligned}$$

for all large enough $r_1$, where we also used that $-\Phi ''\le \tfrac{\pi ^2}{4} \Phi$ on [0, 2) by definition of Φ. Hence

7.7 $$\begin{aligned} -\Delta g \le g \big [ \Delta F - |\nabla F|^2 + \frac{\pi ^2}{4}( 4\gamma ^2 r_1^{-2} + r_1^{-2\gamma }) \big ] \quad \text {on } A_{R}^- \, . \end{aligned}$$

for large enough *R*.

Now the rest of the argument is easy: One checks

$$\begin{aligned} \Delta F = \partial _1^2 F +\partial _1 F \frac{2}{r_1} + \partial _2^2 F + \partial _2 F\frac{2}{r_2} = \partial _1^2 F +\partial _1 F \frac{2}{r_1} + \frac{1}{r_2} \end{aligned}$$

since $\partial _2 F=1/2$. Using this, $1/4-(\partial _2F)^2=0$, and (7.7), we get

7.8 $$\begin{aligned} (H_U-E_U) g&\le g\left[ \varepsilon -(\partial _1 F)^2 +\frac{U}{|x_1-x_2|} -\frac{1}{r_1} + \partial _1^2 F +\partial _1 F \frac{2}{r_1} + \frac{\pi ^2}{4}( 4\gamma ^2 r_1^{-2} + r_1^{-2\gamma }) \right] \end{aligned}$$

On $A_{R}^-$ we have $|x_2|\le 2 |x_1|^\gamma$, so $|x_1-x_2|\ge |x_1|-|x_2| \ge r_1-2r_1^{\gamma }$. Thus

$$\begin{aligned} \frac{U}{|x_1-x_2|} - \frac{1}{r_1} \le \frac{U}{r_1-2r_1^\gamma }-\frac{1}{r_1} = \frac{U-1}{r_1} + \frac{2U r_1^{\gamma -2}}{1-2r_1^{\gamma -1}} \quad \text {on } A_{R}^- \end{aligned}$$

for large enough *R*. Since $\partial _1F = F_U'(r_1)+K\kappa r_1^{\kappa -1}$ and γ<1, we have

$$\begin{aligned} \varepsilon -(\partial _1 F)^2 +\frac{U}{|x_1-x_2|} -\frac{1}{r_1}&\le -2K\kappa _1 F_U'(r_1)r_1^{\kappa -1} + 4U r_1^{\gamma -2} \end{aligned}$$

for all $r_1>0$ such that $1-2r_1^{\gamma -1}\ge 1/2$. The bound $F_U''\le 0$ implies

$$\begin{aligned} \partial _1^2 F +\partial _1 F\frac{2}{r_1}&= F_U''(r_1) + K\kappa (\kappa +1) r_1^{\kappa -2} + 2F_U'(r_1)r_1^{-1} \\&\le 2F_U'(r_1)r_1^{-1} + K\kappa (\kappa +1) r_1^{\kappa -2}, \end{aligned}$$

which leads to

$$\begin{aligned} \varepsilon -(\partial _1 F)^2&+\frac{U}{|x_1-x_2|} -\frac{1}{r_1} + \partial _1^2 F +\partial _1 F\frac{2}{r_1} \\&\lesssim - F_U'(r_1) r_1^{\kappa -1} + r_1^{\gamma -2} + r_1^{\kappa -2} \lesssim -r_1^{\kappa -3/2} + r_1^{\gamma -2} \end{aligned}$$

for large $r_1$, using again the bound $F_U'(r_1)\ge c r_1^{-1/2}$ with some constant c>0, uniformly in $0\le U\le U_c$. Thus from (7.8) we get

$$\begin{aligned} (H_U-E_U) g&\lesssim g\left[ -r_1^{\kappa -3/2} +r_1^{\gamma -2} +r_1^{-2} +r_1^{-2\gamma } \right] \le 0 \quad \text {in } A_{R}^- \end{aligned}$$

for all large enough *R*, under the condition that $\kappa -3/2>\max (\gamma -2, -2\gamma )$, which is equivalent to $(3-2\kappa )/4<\gamma <\kappa +1/2$. Since we have the constraint γ<1, this is equivalent to the condition on the parameters in the range $0\le U\le U_c$.

In the subcritical case we use that $F_U'(r_1)\gtrsim c_\mu >0$, for any $0\le U\le U_c-\mu$ so $F_U'(r_1) r_1^{\kappa -1}\gtrsim r_1^{\kappa -1}$ uniformly in $0\le U\le U_c-\mu$. Using this as before, we see that $(H_U-E_U) g \le 0$ on $A_{R}^-$ for all large enough *R*, under the condition that $\kappa -1>\max (\gamma -2, -2\gamma )$. This is equivalent to $(1-\kappa )/2<\gamma < \kappa +1$. Since we have the constraint 0<γ<1, this is equivalent to the condition on the parameters in the subcritical case. □

## Global Lower Bound

In this section we prove the global pointwise lower bound for the ground state of helium type atoms, including the critical coupling. The Coulomb repulsion of the particles, which can become *arbitrary large* when the particles are close to each other, requires a considerably more complicated ansatz for the subsolution compared to the proof of the lower bound in the *tricky region* in Sect. 7. On the other hand, since we already proved a lower bound in Theorem 7.1 in the tricky region, we can now assume that both $|x|_0$ and $|x|_\infty$ are large, which helps to control the errors.

Recall that for non-negative constants $\kappa _1,\kappa _2, K_1, K_2$, and $s,r_1,r_2\ge 0$ the weight function $F^U_-$ is given by

8.1 $$\begin{aligned} F^U_-(r_1,r_2)&{:}{=}F_U(r_1) +\frac{1}{2} r_2 +K_1 r_1^{\kappa _1} +K_2 r_2^{\kappa _2} \end{aligned}$$

with $F_U$ defined in (1.9) and we consider the couplings in the range $\mu \le U\le U_c$ for some positive μ. Moreover, we will use

8.2 $$\begin{aligned} \theta (s)&{:}{=}\frac{1}{1+s} \end{aligned}$$

8.3 $$\begin{aligned} h(s,r_2)&{:}{=}s\, \theta (r_2^{-\nu } s) \end{aligned}$$

for some 0<ν<1. Recall also $1< U_c\le 2$. Furthermore, we will use

8.4 $$\begin{aligned} L_U(x)= L_U(x_1,x_2) {:}{=}F^U_-(|x|_\infty ,|x|_0) - h(|x_1-x_2|,|x|_0) \end{aligned}$$

and abbreviate

8.5 $$\begin{aligned} g_U{:}{=}\exp (-L_U) \end{aligned}$$

in the remainder of this section. Again, for simplicity of notation, we do not indicate the dependence of $L_U$ and $g_U$ on the other parameters except for *U*, in our notation. Note that $g_U\in H^1({\mathbb {R}}^6)$, but its gradient has a jump discontinuity along the diagonal $|x_1|=|x_2|$, so it is not even locally in $H^2(U)$ for any open set $U\cap \{|x_1|=|x_2|\}\ne \emptyset$. Fortunately, the subharmonic comparison principle which we will use does not require local $H^2$ regularity.

### Proposition 8.1

For all $0<\beta ,\nu <1$, $0<\kappa _1<\kappa _2< 1$, $\max (\nu ,1-\nu )<\kappa _2$, and $K_1,K_2>0$, the function $g_U=\exp (-L_U)$ is a subsolution of $H_U$ at energy $E_U$ in the sense of Agmon in the region

8.6 $$\begin{aligned} C_{R,\beta }{:}{=}\big \{ |x|_0> |x|_\infty ^\beta , |x|_\infty >R \big \} \end{aligned}$$

for all large enough *R* uniformly in $0\le U\le U_c$. That is, for all $0\le \varphi \in H^1(C_{R,\beta })$ one has, as quadratic forms,

8.7 $$\begin{aligned} \langle \varphi , (H_U-E_U)g_U \rangle \le 0 \, \end{aligned}$$

and *R* depends on the parameters $\kappa _1,\kappa _2$, and ν, but not on *U* in the range $0\le U\le U_c$.

Moreover, if $0<\beta ,\nu , \kappa _1,\kappa _2< 1$, $\max (\nu ,1-\nu )<\kappa _2$, and $0<\mu <U_c$ then there exists R>0 independent of *U* in the range $\mu \le U\le U_c$ such that (8.7) again holds.

### Proof

The proof of Proposition 8.1 follows directly from Lemma 8.6 and 8.8 at the end of this section. 6.4□

### Remark 8.2

Since $\max (\nu ,1-\nu )\ge 1/2$ we always have $1/2<\kappa _2<1$ in Proposition 8.1.

The lower bound $U\ge \mu >0$ is needed in the proofs of Lemma 8.6 and 8.8 when $0<\kappa _2\le \kappa _1<1$. It is not needed if $0<\kappa _1<\kappa _2<1$.

The additional term *h* in the comparison function $g_U= \exp (-L_U)$ allows us to control the Coulomb repulsion between the particles. We believe that our choice of subharmonic comparison function $g_U= \exp (-L_U)$ is not only considerably simpler than the one used by Thomas Hoffmann–Ostenhof [24] in his derivation of exponential lower bounds for the ground state of subcritical helium-type systems but it also allows us to get the *sharp coefficients* of the leading order terms in the lower bound!

Moreover, the proof shows that one has considerable flexibility in the choice of θ. The only properties of θ which we need are

1. $\theta \ge 0$ is continuous on [0,∞) and twice differentiable on (0,∞),
2. $$\theta (s)\ge 1/2$$ for $$0\le s\le 1$$,
3. $s\mapsto s\theta (s)$ is increasing and bounded on [0,∞),
4. $s\mapsto (1+s+s^2)|\theta '(s)|$ and $s\mapsto (s+s^2+s^3)|\theta ''(s)|$ are bounded on (0,∞)

Assuming Proposition 8.1 and given the quadratic form version of the sub-harmonic comparison principle, the proof of the following lower bound is relatively straightforward.

### Theorem 8.3

Let $\psi _U$ be the positive ground state of helium–type operator $H_{U}$, i.e, $H_U\psi _U\!=E_U\psi _U$, in the quadratic form sense. Then for $1/6< \kappa _1<\frac{1}{2}$, 0<ν<1, $\max (\nu ,1-\nu )<\kappa _2<1$, and $K_1,K_2>0$, we have the lower bound

8.8 $$\begin{aligned} \psi _U(x)\ge C \exp \big ( -L_U(x) \big ) \end{aligned}$$

for all $x\in {\mathbb {R}}^6$ where $L_U$ is defined in (8.4), the constant *C* depends on the parameters $\kappa _1,\kappa _2,\nu$, and $K_1,K_2$ but not on *U* in the range $0\le U\le U_c$.

Moreover, if $0< \nu , \kappa _1< 1$, $\max (\nu ,1-\nu )<\kappa _2<1$, $K_1,K_2>0$, and $0<\mu <U_c/2$, then we again have the bound (8.8) for some constant *C*, depending on the parameters $\kappa _1,\kappa _2,\nu$, $K_1,K_2$, and μ but not on *U* in the range $\mu \le U\le U_c-\mu$.

### Proof

The lower bound given in Theorem 7.1 shows that uniformly in $0\le U\le U_c$ and for all fixed $1/6<\kappa _1<1/2$, $0<\gamma <\kappa _1+1/2$, and $K_1>0$

8.9 $$\begin{aligned} \psi _U(x)\gtrsim \exp \big (-F_U(|x|_\infty ) -\frac{K_1}{2} |x|_\infty ^{\kappa _1} -\frac{1}{2}|x|_0\big )\, . \end{aligned}$$

for all $|x|_0\le |x|_\infty ^\gamma$. Since $s\theta (s)\le 1$, one sees $0< h(s,r_2)= s \theta (r_2^{-\nu }s)\le r_2^\nu$ uniformly in s≥0. Thus, since $0<\nu <\kappa _2$,

$$\begin{aligned}&F_U(r_1) + K_1 r_1^{\kappa _1} +\frac{1}{2}r_2 \le F^U_-(r_1,r_2) -h(s,r_2) + r_2^\nu -K_2r_2^{\kappa _2}\\&\quad \le F^U_-(r_1,r_2) - h(s,r_2) +C \end{aligned}$$

for all $s,r_1,r_2 \ge 0$ and some constant $0\le C<\infty$. This proves the lower bound (8.8) in the tricky region $\widetilde{A}=\{|x|_0\le |x|_\infty ^\gamma \}$ for any $0<\gamma <\kappa _1+1/2$.

The same argument applies, for subcritical *U* uniformly in the range $0\le U\le U_c-\mu$, for small fixed μ>0, for all fixed $0<\gamma ,\kappa _1<1$ and $0<\nu <\kappa _2$.

Let $0<\beta <\gamma$ and abbreviate $C_R=C_{R,\beta }=\{ |x|_0> |x|_\infty ^\beta , |x|_\infty >R \}$ for the remainder of this proof. Its boundary is given by $\partial C_R= \partial C_R^1\cup \partial C_R^2$ with $\partial C_R^1=\{ R^\beta \le |x|_0\le R, |x|_\infty =R\}$, which is compact, and $\partial C_R^2=\{ |x|_0=|x|_\infty ^\beta , |x|_\infty >R \}$, which is *unbounded*. Since the bound (8.8) holds on the tricky region $\widetilde{A}$ it clearly holds on $\partial C_R^2$, that is, there exist a constant $C_1>0$ such that

$$\begin{aligned} \psi _U(x)\ge C_1 g_U(x) \quad \text {for all } x\in \partial C_R^2\, \end{aligned}$$

where the constant $C_1$ does not depend on *U*. Moreover, since $\psi _U$ is bounded away from zero uniformly in $0\le U\le U_c$ on compact sets, see Proposition D.1, and $g_U$ is continuous and $\partial C_R^1$ is compact, there exist a constant $C_2>0$ such that

$$\begin{aligned} \psi _U(x)\ge C_2 g_U(x) \quad \text {for all } x\in \partial C_R^1\,, \end{aligned}$$

with $C_2$ independent of *U*. Hence, for some constant C>0 we have $\psi _U(x)\ge 2C g_U(x)$ for all $x\in \partial C_R$. By continuity of $\psi _U$ and $g_U$, there exists a boundary layer $\widetilde{\partial C}_R$ such that

8.10 $$\begin{aligned} \psi _U(x)\ge C g_U(x) \quad \text {for all } x\in \widetilde{\partial C}_R \, . \end{aligned}$$

Such a boundary layer is an open subset of $C_R$ near the boundary $\partial C_R$ with $\textrm{dist}(x,\partial C_R)>0$ for all $x\in C_R\setminus \widetilde{\partial C}_R$, see Appendix Appendix B.

Due to Proposition 8.1 we can use Theorem B.3, to extend the bound (8.10) to almost all $x\in C_R$. Since $\psi _U$ and $g_U=\exp (-L_U)$ are continuous, this lower bound clearly holds for all $x\in C_R$ and since we already showed that the same type of lower bound holds in the region $\widetilde{A}=\big \{|x|_0\le |x|_\infty ^\gamma \big \}$, it holds on $C_R\cup \widetilde{A}$.

The complement of $C_R\cup \widetilde{A}$ in ${\mathbb {R}}^6$ is bounded, hence its closure is compact. Using Proposition D.1 one sees that $\psi _U$ is bounded away from zero on compact sets and since $g_U$ is bounded uniformly in $0\le U\le U_c$, a bound of the form $\psi _U\ge C g_U$ also holds on ${\mathbb {R}}^6\setminus (C_R\cup \widetilde{A})$. Thus, at the expense of decreasing the constant C>0, if necessary, the lower bound (8.8) holds globally uniformly in in the critical range $0\le U\le U_c$.

In the subcritical case, where we allow for a wider range of parameters $\kappa _1,\kappa _2$, the same arguments apply, but now *U* has to stay away from both $U_c$ and zero. That is, we have to restrict the range of allowed parameters *U* to $\mu \le U\le U_c-\mu$ for arbitrary but fixed small μ, due to the additional lower bound on *U* in Proposition 8.1 and the upper bound on *U* from Theorem 7.1. □

Now we come to the proof of the global lower bound.

### Theorem 8.4

(Sharp lower bound, arbitrary coupling) For any choice of parameters $K_1,K_2>0$, $1/6<\kappa _1<1/2$, and $1/2<\kappa _2<1$ there exist a constant $C_-$ depending only on $\kappa _1,\kappa _2, K_1,$ and $K_2$, such that for the unique positive choice of the ground state of the helium-type operator $H_U$ the pointwise bound

8.11 $$\begin{aligned} \psi _U(x)\ge C_- \exp \left( -F^U_-(|x|_\infty ,|x|_0) \right) \end{aligned}$$

holds uniformly in $\mu \le U\le U_c$.

For the subcritical case let $0<\kappa _1<1$, $1/2<\kappa _2<1$, $K_1,K_2>0$, and $0<\mu <U_c/2$ be arbitrary. Then there exist a constant $\widetilde{C}_-$, depending only on $\kappa _1,\kappa _2, K_1, K_2$, and also on μ, such that the lower bound

8.12 $$\begin{aligned} \psi _U(x)\ge \widetilde{C}_- \exp \left( -F^U_-(|x|_\infty ,|x|_0) \right) \end{aligned}$$

holds for all $\mu \le U\le U_c -\mu$.

### Remark 8.5

Note that Theorem 1.2 is a special case of Theorem 8.4 for $U=U_c$.

### Proof of Theorem 8.4:

Given $1/6<\kappa _1<1/2<\kappa _2<1$ we choose any 0<ν<1 with $\max (\nu ,1-\nu )<\kappa _2$. Theorem 8.3 gives the global lower bound $\psi _U\ge C \exp (-L_U) =C\exp (-F^U_- +h)\ge C \exp (-F^U_-)$, since h≥0 with the constant *C* from Theorem 8.3 which does not depend on *U* in the range $0\le U\le U_c$. The same argument applies for the subcritical range $\mu \le U\le U_c-\mu$. □

We still have to give the proof of Proposition 8.1. This will be done in two steps. We split the region $C_{R,\beta }$ in two subregions

8.13 $$\begin{aligned} C_{R,\beta }^-&{:}{=}\big \{ |x_1|^\beta< |x_2|<|x_1|,\, |x_1|>R \big \}\, , \end{aligned}$$

8.14 $$\begin{aligned} C_{R,\beta }^+&{:}{=}\big \{ |x_2|^\beta< |x_1|<|x_2|,\, |x_2|>R \big \}\, , \end{aligned}$$

and the diagonal

8.15 $$\begin{aligned} D_R&{:}{=}\big \{ |x_1|= |x_2|>R \big \}\, . \end{aligned}$$

While $e^{-L_U}$ with $L_U$ defined in (8.4) is not $H^2(C_{R,\beta })$, due to the jump of the gradient of $L_U$ along the diagonal $D_R$, it is twice differentiable in $C_{R,\beta }^\pm$. The next lemma shows that $e^{-L_U}$ is a subsolution on $C_{R,\beta }$ in the quadratic form sense a la Agmon, see [2] and Appendix Appendix B, as soon as it is a classical subsolution locally on $C_{R,\beta }^\pm$, that is, $(H_U -E_U) e^{-L_U}\le 0$ on $C_{R,\beta }^+$ and on $C_{R,\beta }^-$ separately.

### Lemma 8.6

Let 0<β<1 and assume that $g_U{:}{=}e^{-L_U}$ is a classical subsolution of $H_U$ at energy $E_U$, i.e., $(H_U-E_U)g_U\le 0$ pointwise, both in $C_{R,\beta }^+$ and $C_{R,\beta }^-$.

If $0<\nu ,\kappa _1<\kappa _2<1$, then $g_U$ is a subsolution in the quadratic form sense a la Agmon on $C_{R,\beta }$ for any large enough R>0 independent of $0\le U\le U_c$. That is

8.16 $$\begin{aligned} \langle \varphi , (H_U-E_U) g_U \rangle \le 0 \end{aligned}$$

for all $$0\le \varphi \in \mathcal {C}^\infty _0(C_{R,\beta })$$.

Moreover, if $0<\nu ,\kappa _1,\kappa _2<1$ and $0<\mu <U_c$ then there exists R>0 independent of *U* in the range $\mu \le U\le U_c$ such that (8.16) again holds.

### Proof

We will write *g*, *L*, and *F* for $g_U$, $L_U$, respectively, $F^U_-$ in the remainder of this proof, for simplicity of notation. The contribution of the Coulomb potential is local. So we only have to show that for *R* large enough,

8.17 $$\begin{aligned} \langle \nabla \varphi , \nabla g\rangle _{L^2(C_{R,\beta })} \le \langle \varphi , -\Delta g\rangle _{L^2(C_{R,\beta }^+)} + \langle \varphi , -\Delta g\rangle _{L^2(C_{R,\beta }^-)} \end{aligned}$$

for all $\varphi \in \mathcal {C}^\infty _0(C_{R,\beta })$, since then for all $\varphi \ge 0$ the pointwise bounds $(H_U-E_U)g\le 0$ in $C_{R,\beta }^\pm$ together with (8.17) will imply (8.16). The bound (8.17) follows from splitting the domain $C_{R,\beta }$ into two pieces along the diagonal $D_R=\{|x_2|=|x_1|>R\}$ and integration by parts, since the boundary term on $D_R$ has the right sign when *R* is large enough, as we will show in a moment. Thus we can drop the boundary term to get (8.17).

Given $x=(x_1,x_2)\in D_R$, the vectors

8.18 $$\begin{aligned} n_-(x){:}{=}\frac{1}{\sqrt{2}}\begin{pmatrix} \frac{-x_1}{|x_1|} \\ \frac{x_2}{|x_2|} \end{pmatrix} \quad \text {and } n_+(x){:}{=}\frac{1}{\sqrt{2}}\begin{pmatrix} \frac{x_1}{|x_1|} \\ \frac{-x_2}{|x_2|} \end{pmatrix} \end{aligned}$$

are the outer normals of $C_{R,\beta }^-$, respectively $C_{R,\beta }^+$, on the diagonal $D_R$. To see this simply note that level sets $\{N(x)=\lambda \}\cap C_{R,\beta }^-$ with λ<1 of the function $N(x)= |x_2|/|x_1|$ converge to the diagonal $D_R$ from within $C_{R,\beta }^-$ as $\lambda \nearrow 1$. The gradient of *N* points into the direction of the largest increase, hence the vector $n_-(x)$ is a positive, and $n_+(x)$ a negative, multiple of ∇N(x) when $x=(x_1,x_2)\in D_R$. This proves (8.18), because

$$\begin{aligned} \nabla N(x) = \begin{pmatrix} \frac{-|x_2|}{|x_1|^2} \frac{x_1}{|x_1|} \\ \frac{1}{|x_1|}\frac{x_2}{|x_2|} \end{pmatrix} = \frac{1}{|x_1|}\begin{pmatrix} \frac{-x_1}{|x_1|} \\ \frac{x_2}{|x_2|} \end{pmatrix} \end{aligned}$$

when $$|x_1|=|x_2|$$. Since

8.19 $$\begin{aligned} \langle \nabla \varphi , \nabla g\rangle _{L^2(C_{R,\beta })} = \langle \nabla \varphi , \nabla g\rangle _{L^2(C_{R,\beta }^-)}+ \langle \nabla \varphi , \nabla g\rangle _{L^2(C_{R,\beta }^+)}\, , \end{aligned}$$

Gauß’ formula, i.e., integration by parts, gives

8.20 $$\begin{aligned} \langle \nabla \varphi , \nabla g\rangle _{L^2(C_{R,\beta }^-)} = \langle \varphi , -\Delta g\rangle _{L^2(C_{R,\beta }^-)} +\int _{D_R}\varphi \nabla g \cdot n_-\, dS \end{aligned}$$

where *S* is 5-dimensional Hausdorff (surface) measure on $D_R$. There are no contributions from the other parts of the boundary of $C_{R,\beta }$, since φ vanishes there. Similarly,

8.21 $$\begin{aligned} \langle \nabla \varphi , \nabla g\rangle _{L^2(C_{R,\beta }^+)} = \langle \varphi , -\Delta g\rangle _{L^2(C_{R,\beta }^+)} +\int _{D_R}\varphi \nabla g \cdot n_+\, dS \, . \end{aligned}$$

Thus (8.17) will follow once the boundary integrals in (8.20) and (8.21) are non-positive for all $\varphi \ge 0$ and large enough *R*. By symmetry, it is enough to show this for the boundary integral in (8.20).

Clearly $\nabla g = -e^{-L}\nabla L$. Using $\varphi \ge 0$ and $e^{-L}\ge 0$, one sees that the second term in (8.20) is non-positive as soon as

8.22 $$\begin{aligned} \nabla L \cdot n_- \ge 0 \quad \text {on } D_R\, . \end{aligned}$$

On $C_{R,\beta }^-$ the function *L* is given by

8.23 $$\begin{aligned} L(x)= F(|x_1|, |x_2|) - h(|x_1-x_2|, |x_2|) \end{aligned}$$

with $$h(s,r_2)= s \theta (s^{-\nu }s)$$. So

8.24 $$\begin{aligned} \nabla L = \begin{pmatrix} \nabla _{x_1}(F -h) \\ \nabla _{x_2}(F -h) \end{pmatrix} = \begin{pmatrix} \partial _1 F \frac{x_1}{|x_1|} - \partial _s h\frac{x_1-x_2}{|x_1-x_2|} \\ \partial _2 F \frac{x_2}{|x_2|} - \partial _2h \frac{x_2}{|x_2|} -\partial _s h\frac{x_2-x_1}{|x_1-x_2|} \end{pmatrix}\, , \end{aligned}$$

hence on $D_R$ we have

8.25 $$\begin{aligned} \begin{aligned} \sqrt{2}\nabla L\cdot n_-&= - \partial _1 F + \partial _s h\frac{x_1\cdot (x_1-x_2)}{|x_1||x_1-x_2|} + \partial _2 F - \partial _2h -\partial _s h\frac{x_2\cdot (x_2-x_1)}{|x_2||x_1-x_2|} \\&= - \partial _1 F + \partial _2 F - \partial _2 h +\partial _s h\frac{x_1\cdot (x_1-x_2)-x_2\cdot (x_2-x_1)}{|x_1||x_1-x_2|} \end{aligned} \end{aligned}$$

using also $|x_1|=|x_2|$ on $D_R$. Since $x_1\cdot (x_1-x_2)- x_2\cdot (x_2-x_1)= |x_1|^2-|x_2|^2= 0$ on $D_R$ this simplifies to

8.26 $$\begin{aligned} \sqrt{2}\nabla L\cdot n_- =- \partial _1 F + \partial _2 F - \partial _2 h \, . \end{aligned}$$

Also

8.27 $$\begin{aligned} 0\le \partial _2 h = \partial _2(s\theta (r_2^{-\nu }s)) = -\nu r_2^{\nu -1} (r_2^{-\nu }s)^2\theta '(r_2^{-\nu }s) \lesssim r_2^{\nu -1} \end{aligned}$$

by our choice of θ. Thus, with $r_1=|x_1|=|x_2|=r_2$, we get for all large enough $r_1$

$$\begin{aligned} \sqrt{2}\nabla&L\cdot n_- \ge - \partial _1 F + \partial _2 F -\partial _2 h \ge -F_U'(r_1) -K_1\kappa _1r_1^{\kappa _1-1} +\frac{1}{2} +K_2\kappa _2 r_2^{\kappa _2-1} - Cr_1^{\nu -1} \\&= -\left( \varepsilon +\frac{a^2}{r_1}\right) ^{1/2} +\frac{1}{2} -K_1\kappa _1r_1^{\kappa _1-1} +K_2\kappa _2 r_1^{\kappa _2-1} - Cr_1^{\nu -1} > 0 \end{aligned}$$

where we used the bound (8.28) below, $r_2= r_1$ is large, and $0<\nu ,\kappa _1<\kappa _2<1$ uniformly in $0\le U\le U_c$. If *U* stays away from zero, we also get $\sqrt{2}\nabla L\cdot n_- \ge 0$ uniformly in *U* in the range $\mu \le U\le U_c$ for large $r_1$, any small μ>0, and $0<\nu ,\kappa _1,\kappa _2<1$, using (8.29).

Thus the second term in (8.20), hence by symmetry also the second term in (8.21), is non-positive when *R* is large enough. □

### Remark 8.7

In the proof above, and also in the proof of Lemma 8.8 below the inequality

8.28 $$\begin{aligned} \sup _{0\le U\le U_c}\Big (\varepsilon _U+ \frac{a_U^2}{r_1}\Big ) \le \frac{1}{4}\, \end{aligned}$$

for all large enough $r_1$ plays an important role. Also the refined bound

8.29 $$\begin{aligned} \limsup _{r_1\rightarrow \infty }\sup _{\mu \le U\le U_c} \Big (\varepsilon _U+ \frac{a_U^2}{r_1}\Big ) < \frac{1}{4} \end{aligned}$$

for any small μ>0. The second bound clearly implies that

8.30 $$\begin{aligned} c_\mu = \liminf _{r_1\rightarrow \infty }\inf _{\mu \le U\le U_c} \Big (\frac{1}{2}- \Big (\varepsilon _U+ \frac{a_U^2}{r_1}\Big )^{1/2} \Big ) > 0 \end{aligned}$$

for any $0<\mu <U_c$ which is needed in the proof of Lemma 8.8 below.

These bounds can be seen as follows: Since the Coulomb repulsion is positive and the ground state energy of $H_0$ is -1/2 (twice the energy of hydrogen), we clearly have $E_U\ge E_0= -1/2$, so $\varepsilon _U = -1/4-E_U\le 1/4$, always. Since $E_U$ is strictly increasing in $0\le U\le U_c$, by the Hellman-Feynman formula, we also have $\varepsilon _U= -1/4-E_U<-1/4-E_{\mu }=\varepsilon _{\mu }<1/4$ for any $0\le \mu <U\le U_c$. Since $a_U=(U-1)_+^{1/2}$ we get

$$\begin{aligned} \sup _{\mu \le U\le U_c} (\varepsilon _U +\frac{a_U^2}{r_1})\le \varepsilon _\mu + \frac{U_c-1}{r_1} <\frac{1}{4} \end{aligned}$$

for all large enough $r_1>0$, which immediately implies (8.29). Moreover, for $0\le U\le 1$ we have

$$\begin{aligned} \varepsilon _U+ \frac{a_U^2}{r_1} = \varepsilon _U\le \varepsilon _0= \frac{1}{4} \end{aligned}$$

for all $r_1>0$. Since $U_c\le 2$, we get for $1\le U\le U_c$,

$$\begin{aligned} \varepsilon _U+ \frac{a_U^2}{r_1} \le \varepsilon _1+ \frac{1}{r_1} < \frac{1}{4} \end{aligned}$$

for all large enough $r_1$, independently of $1\le U\le U_c$, since $\varepsilon _U\le \varepsilon _1<1/4$ for U≥1. The last two bounds prove (8.28).

### Lemma 8.8

Assume $0<\beta ,\nu <1$, $0<\kappa _1<\kappa _2< 1$ and $\max (\nu ,1-\nu )<\kappa _2$ and $L_U$ given by (8.4). Then $g_U= e^{-L_U}$ is a classical subsolution of $H_U$ at energy $E_U$ on $C_{R,\beta }^\pm$ for all large *R* independently of $0\le U\le U_c$.

Moreover, if $0<\beta ,\nu , \kappa _1,\kappa _2< 1$, $\max (\nu ,1-\nu )<\kappa _2$, and $0<\mu <U_c$ then there exists R>0 independent of *U* in the range $\mu \le U\le U_c$ such that $g_U$ is again a classical subsolution of $H_U$ at energy $E_U$ on $C_{R,\beta }^\pm$.

### Proof

Again, we abbreviate $g=g_U$, $L=L_U$, and $F=F^U_-$. Clearly, *L* and hence $g= e^{-L}$ is $\mathcal {C}^2$ on $C_{R,\beta }^\pm$ and the derivatives of *g* up to order two are in $L^2(C_{R,\beta }^\pm )$ for large enough *R*. Moreover, we only have to show the pointwise bound $(H_U-E_U)g\le 0$ in $C_{R,\beta }^-$, since by symmetry the same bound then also holds on $C_{R,\beta }^+$.

From $\nabla g= -e^{-L}\nabla L$, one gets

8.31 $$\begin{aligned} -\Delta g = g\left[ -|\nabla L|^2 +\Delta L \right] \, . \end{aligned}$$

On $C_{R,\beta }^-$ we have $L(x) = F(|x_1|,|x_2|) - h(|x_1-x_2|,|x_2|)$. Using formula (8.24) for ∇L we have

8.32 $$\begin{aligned} \begin{aligned} \Delta L&= \nabla _{x_1} \left( \partial _1 F \frac{x_1}{|x_1|} - \partial _s h\frac{x_1-x_2}{|x_1-x_2|} \right) \\&+ \nabla _{x_2} \left( \partial _2 F \frac{x_2}{|x_2|} - \partial _2h \frac{x_2}{|x_2|} -\partial _s h\frac{x_2-x_1}{|x_1-x_2|} \right) \\&= \partial _1^2 F + \partial _1 F \frac{2}{|x_1|} + \partial _2^2 F + \partial _2 F \frac{2}{|x_2|} -\partial _2^2 h - \partial _2 h \frac{2}{|x_2|} - 2\partial _s^2 h \\&-2\partial _2\partial _s h \frac{x_2\cdot (x_2-x_1)}{|x_2||x_1-x_2|} - \partial _s h \frac{4}{|x_1-x_2|} \, . \end{aligned} \end{aligned}$$

The term $\partial _s h \tfrac{4}{|x_1-x_2|}$ will allow us to control the Coulomb repulsion between the electrons. Dropping the positive term $(\partial _s h)^2$ we also get from (8.24)

$$\begin{aligned} |\nabla L|^2&= |\nabla _{x_1}L|^2+ |\nabla _{x_2}L|^2 \\&\ge (\partial _1 F)^2 -2\partial _s h\partial _1 F \frac{x_1\cdot (x_1-x_2)}{|x_1||x_1-x_2|} + (\partial _2 F -\partial _2 h)^2 \\&\quad -2(\partial _2 F -\partial _2 h)\partial _s h \frac{x_2\cdot (x_2-x_1)}{|x_2||x_1-x_2|} \, \end{aligned}$$

hence

8.33 $$\begin{aligned} \begin{aligned} \big ( H_U-E_U\big ) g&= \big ( -\Delta -\frac{1}{|x_1|} -\frac{1}{|x_2|} +\frac{U}{|x_1-x_2|} +\frac{1}{4} +\varepsilon \big ) g\\&\le g\Big [ \underbrace{\varepsilon -(\partial _1 F)^2}_{\textrm{I}} + \underbrace{ \frac{1}{4}-(\partial _2 F -\partial _2 h)^2}_{\textrm{II}} \\&+ \underbrace{2\partial _s h (\partial _1 F \frac{x_1\cdot (x_1-x_2)}{|x_1||x_1-x_2|} +(\partial _2 F-\partial _2 h)\frac{x_2\cdot (x_2-x_1)}{|x_2||x_1-x_2|} }_{\textrm{III}} \\&+\underbrace{\partial _1^2 F +\partial _1 F\frac{2}{|x_1|} -\frac{1}{|x_1|}}_{\textrm{IV}} + \underbrace{\partial _2^2 F + \partial _2 F \frac{2}{|x_2|} -\frac{1}{|x_2|}}_{\textrm{V}} \\&-\underbrace{\partial _2^2 h - \partial _2 h \frac{2}{|x_2|} - 2\partial _s^2 h}_{\textrm{VI}} - \underbrace{2\partial _2\partial _s h \frac{x_2\cdot (x_2-x_1)}{|x_2||x_1-x_2|}}_{\textrm{VII}} \\&+ \underbrace{ \frac{U}{|x_1-x_2|} - \partial _s h \frac{4}{|x_1-x_2|} }_{\textrm{VIII}} \Big ] \, . \end{aligned} \end{aligned}$$

Abbreviating $r_1=|x_1|, r_2=|x_2|, s=|x_1-x_2|$, we have

$$\begin{aligned} \partial _1 F = F_U'(r_1) +K_1\kappa _1 r_1^{\kappa _1-1}, \end{aligned}$$

and since $F_U'(r_1)=\left( \varepsilon +a^2/r_1 \right) ^{1/2}\ge 0$ we have $\varepsilon -(F_U'(r_1))^2\le 0$. Hence

$$\begin{aligned} \textrm{I}\le 0 \, . \end{aligned}$$

Moreover,

$$\begin{aligned} \partial _2 F&= \frac{1}{2} + K_2\kappa _1 r_2^{\kappa _2-1}\, , \\ \partial _2 h&= -\nu r_2^{\nu -1}(r_2^{-\nu }s)^2 \theta '(r_2^{-\nu }s) \le \nu r_2^{\nu -1}\, , \end{aligned}$$

where the last bound holds since $0\le t\mapsto t^2 |\theta '(t)|\le 1$. Since $\nu<\kappa _2<1$, we get

$$\begin{aligned} \textrm{II}&= \big (\frac{1}{2} -\partial _2F+\partial _2h\big )\big (\frac{1}{2}+\partial _2F -\partial _2 h\big ) \\&\le -\big (K_2\kappa _2 r_2^{\kappa _2-1} -\nu r_2^{\nu -1}\big )\big ( 1+K_2\kappa _2 r_2^{\kappa _2-1} -\nu r_2^{\nu -1} \big ) \lesssim -r_2^{\kappa _2-1} \end{aligned}$$

for $$r_2 \gg 1$$.

The bound for the third term $\textrm{III}$ is tricky, since neither $\partial _1 F$, $\partial _2F$ nor $\partial _s h$ go to zero as $r_1,r_2\rightarrow \infty$. We will do this one last and bound the other terms first. Since $\partial _1 F = F_U'(r_1) + K_1\kappa _1 r_1^{\kappa _1-1}$ we get

$$\begin{aligned} \partial _1^2 F&= F_U''(r_1) -K_1\kappa _1(1-\kappa _1)r_1^{\kappa _1-2} \le 0\, , \end{aligned}$$

since $\kappa _1<1$ and $F_U''\le 0$. Thus

$$\begin{aligned} \textrm{IV}\le (2F_U'(r_1)-1) r_1^{-1} = \left( 2\big (\varepsilon +\frac{a^2}{r_1}\big )^{1/2}-1\right) r_1^{-1}\le 0 \end{aligned}$$

for $r_1\gg 1$ because of (8.28). Similarly,

$$\begin{aligned} \textrm{V} = K_2\kappa _2(\kappa _2-1)r_2^{\kappa _2-2} +\big (\frac{1}{2} +K_2\kappa _2 r_2^{\kappa _2-1}\big ) \frac{2}{r_2} - r_2^{-1} = K_2\kappa _2(\kappa _2+1) r_2^{\kappa _2-2}\, . \end{aligned}$$

Moreover, with $t=r_2^{-\nu }s$, we have

$$\begin{aligned} |\partial _2^2 h|&\lesssim r_2^{\nu -2}\big (|t^2|\theta '(t)| + t^3|\theta ''(t)| \big ) \lesssim r_2^{\nu -2}\, ,\\ |\partial _s^2 h|&= r_2^{-\nu }\big | 2\theta '(t) + t\theta ''(t) \big | \lesssim r_2^{-\nu } \end{aligned}$$

since $\theta^{\prime }\left(t\right)$ and $t\theta^{\prime \prime }\left(t\right)$ are bounded for t≥0. Thus we get

$$\begin{aligned} \textrm{VI} \lesssim r_2^{\nu -2} + r_2^{-\nu } \end{aligned}$$

for all $r_2\gg 1$ and since

$$\begin{aligned} |\partial _2\partial _s h| = \nu r_2^{-1}\big | 2 t\theta '(t) +t^2\theta ''(t) \big | \lesssim r_2^{-1} \end{aligned}$$

we have

$$\begin{aligned} \textrm{VII} \lesssim r_2^{-1}\, . \end{aligned}$$

For the term $\textrm{VIII}$ which contains the Coulomb repulsion, we have

$$\begin{aligned} \textrm{VIII} = \frac{1}{s}\big ( U- 4\theta (r_2^{-\nu }s)\big ) - 4r_2^{-\nu } \theta '(r_2^{-\nu }s) \le \frac{1}{s}\big ( 2- 4\theta (r_2^{-\nu }s)\big ) + 4r_2^{-\nu } \, . \end{aligned}$$

Recall that $U\le U_c\le 2$. If $r_2^{-\nu }s\le 1$, we have $\theta (r_2^{-\nu }s)\ge 1/2$, hence also $2-4\theta ((r_2^{-\nu }s))\le 0$. If $r_2^{-\nu }s\ge 1$ we have $s^{-1}(2-4\theta (r_2^{-\nu }s))\le 2r_2^{-\nu }$. Altogether,

$$\begin{aligned} \textrm{VIII}\le 2 r_2^{-\nu } + 4r_2^{-\nu } = 6 r_2^{-\nu } \end{aligned}$$

for all $$r_2>0$$.

Now we come to the term $\textrm{III}$, which is the hardest term to bound. Recall that $|\partial _2 h|\lesssim r_2^{\nu -1}$. Moreover, $\partial _sh= \theta (r_2^\nu s)+r_2^{-\nu }s \theta '(r_2^{-\nu }s)\ge 0$, so $|\partial _sh|\lesssim 1$. Thus,

$$\begin{aligned} \textrm{III}&= 2\partial _sh \left( \partial _1 F \frac{x_1\cdot (x_1-x_2)}{|x_1||x_1-x_2|} +\partial _2 F\frac{x_2\cdot (x_2-x_1)}{|x_2||x_1-x_2|} \right) -\partial _sh \partial _2 h\frac{x_2(x_2-x_1)}{|x_2||x_1-x_2|} \\&\le 2\partial _sh \left( \partial _1 F \frac{x_1\cdot (x_1-x_2)}{|x_1||x_1-x_2|} +\partial _2 F\frac{x_2\cdot (x_2-x_1)}{|x_2||x_1-x_2|} \right) +C r_2^{\nu -1}\,. \end{aligned}$$

Note also that

$$\begin{aligned} x_1\cdot (x_1-x_2)&= \frac{1}{2}\left( x_1^2-x_2^2 +|x_1-x_2|^2\right) \end{aligned}$$

and

$$\begin{aligned} x_2\cdot (x_2-x_1)&= \frac{1}{2}\left( x_2^2-x_1^2 +|x_1-x_2|^2\right) \, , \end{aligned}$$

so with $r_1^2=x_1^2 \ge r_2^2= x_2^2$ we have

$$\begin{aligned} \partial _1 F \frac{x_1\cdot (x_1-x_2)}{|x_1||x_1-x_2|} +\partial _2 F\frac{x_2\cdot (x_2-x_1)}{|x_2||x_1-x_2|}&\!= \!\! \left( \frac{\partial _1F}{2r_1} - \frac{\partial _2 F}{2r_2}\right) \! \frac{r_1^2-r_2^2}{|x_1-x_2|} + \left( \frac{\partial _1F}{2r_1} + \frac{\partial _2 F}{2r_2}\right) \!|x_1-x_2| \, . \end{aligned}$$

Due to $r_2\le r_1$ and (8.28), we have uniformly in $0\le U\le U_c$

$$\begin{aligned} \frac{\partial _1F}{r_1} - \frac{\partial _2 F}{r_2}&= \frac{(\varepsilon +\frac{a^2}{r_1})^{1/2}}{r_1} + K_1\kappa _1 r_1^{\kappa _1-2} - \frac{1}{2r_2} -K_2\kappa _2 r_2^{\kappa _2-2}\\&\le r_1^{-1}\left[ \Big (\varepsilon +\frac{a^2}{r_1} \Big )^{1/2} -\frac{1}{2} + K_1\kappa _1 r_1^{\kappa _1-1} - K_2\kappa _2 r_1^{\kappa _2-1} \right] \lesssim - r_1^{\kappa _2-1} < 0 \end{aligned}$$

for all large $r_1$ when $0<\kappa _1<\kappa _2<1$.

When $0<\kappa _1,\kappa _2<1$ and *U* stays away from zero, we again get $\frac{\partial _1F}{r_1} - \frac{\partial _2 F}{r_2}\le -r_1^{-1}\, c_\mu /2 < 0$ uniformly in *U* in the range $\mu \le U\le U_c$ for large $r_1$ and small μ>0 using now (8.30).

Thus with $s=|x_1-x_2|$ we see that

$$\begin{aligned} \textrm{III}&\le s\partial _s h \left( \frac{\partial _1F}{r_1} + \frac{\partial _2 F}{r_2}\right) +C r_2^{\nu -1} \end{aligned}$$

for large $r_1$. Since $\partial _s h = \theta (r_2^{-\nu } s)+r_2^{-\nu }s \theta '(r_2^{-\nu }s)\ge 0$ we have

$$\begin{aligned} s\partial _s h = r^\nu \big ( (r_2^{-\nu }s) \theta (r_2^{-\nu } s)+(r_2^{-\nu }s)^2 \theta '(r_2^{-\nu }s)\big )\lesssim r_2^\nu \end{aligned}$$

uniformly in s≥0. Moreover, $\partial _1 F r_1^{-1}\lesssim r_1^{-1}+r_1^{\kappa _1-2}$ and $\partial _2 F r_2^{-1}\lesssim r_2^{-1}+r_2^{\kappa _2-2}$, so we arrive at

$$\begin{aligned} \textrm{III} \lesssim r_2^{\nu }\left[ r_1^{-1}+r_1^{\kappa _1-2}+ r_2^{-1}+r_2^{\kappa _2-2} \right] \lesssim r_2^{\nu -1} + r_2^{\nu +\kappa _1-2} + r_2^{\nu +\kappa _2-2} \end{aligned}$$

since $$\kappa _1, \kappa _2<1$$ and $$r_2\le r_1$$.

Collecting the above bounds in (8.33) we finally arrive at

$$\begin{aligned} \big ( H_U +E_U\big ) g&\lesssim g \big [ -r_2^{\kappa _2-1} + r_2^{\nu -1} + r_2^{\nu +\kappa _1-2} + r_2^{\nu +\kappa _2-2} + r_2^{\kappa _2-2} + r_2^{\nu -2} + r_2^{-\nu } + r_2^{-1} \big ] \\&\le g r_2^{\kappa _2-1}\!\! \left[ -1 \!+ r_2^{\nu -\kappa _2} + r_2^{\nu +\kappa _1-\kappa _2-1}+ r_2^{\nu -1} +r_2^{-1} + r_2^{\nu -1-\kappa _2} + r_2^{1-\nu -\kappa _2} + r_2^{-\kappa _2} \right] \\&< 0 \end{aligned}$$

for all large enough $r_2$, since $0<\nu ,\kappa _1,\kappa _2<1$ and $\max (\nu ,1-\nu )<\kappa _2<1$. This proves the lemma. □

### Remark 8.9

The lower bound $U\ge \mu >0$ on the electron electron repulsion is needed in the last bound on the third term $\textrm{III}$, due to the bound (8.30) in Remark 8.7.

## Data Availability

All data generated or analyzed during this study are included in this article.

## A Quadratic Form Version of the IMS Localization Formula

In this section we derive the well-known IMS localization formula under rather weak assumptions on the localization functions. Our derivation is motivated by [20] who used quadratic form methods to derive an IMS localization formula for pseudo–relativistic Schrödin-ger operators under weak assumptions on the localization functions. While we believe that the result for non-relativistic Schrödinger operators is known, at least to the experts, we could not find any reference in the literature for the version we need.

In the following, we consider the momentum operator P=-i∇ on ${\mathbb {R}}^d$ and acting in $L^2({\mathbb {R}}^d)$ for general $d\in {\mathbb {N}}$. The domain of *P* is the Sobolev space $\mathcal {D}(P)=H^1({\mathbb {R}}^d)=H^1$. The kinetic energy is given by the the quadratic form $\langle P\varphi , P\varphi \rangle$, which is a closed quadratic form on $\mathcal {D}(P)$. In order to be able to even formulate the IMS localization formula, on needs to have weight functions $\xi :{\mathbb {R}}^d\rightarrow {\mathbb {R}}$ such that $\xi ^2\varphi , \xi \varphi \in \mathcal {D}(P)$ for any $\varphi \in \mathcal {D}(P)$.

### Lemma A.1

Assume that $\xi :{\mathbb {R}}^d\rightarrow {\mathbb {R}}$ is bounded and continuous and there exist $k\in {\mathbb {N}}$ and pairwise disjoint open sets $U_j$, j=1,…,k with Lipshitz boundary, such that ${\mathbb {R}}^d=\cup _{j} \overline{U_j}$ and

A.1 $$\begin{aligned} \xi \big {|}_{U_j} \in W^{1,\infty }(U_j) \quad \text {for all } j=1,\ldots ,k \end{aligned}$$

that is, the weak derivative $\nabla \xi$ is bounded on each $U_j$, j=1,…,k. Then $\xi \in W^{1,\infty }({\mathbb {R}}^d)$ and for all $\varphi \in \mathcal {D}(P)$ we have $\xi ^2\varphi , \xi \varphi \in \mathcal {D}(P)$, that is, as multiplication operators $\xi ^2$ and ξ map $\mathcal {D}(P)$ into itself.

### Proof

First, we show that the weak derivative of ξ exists and is given by the function $\widetilde{ \nabla \xi }$ defined by $\widetilde{ \nabla \xi }{:}{=}\nabla \xi$ on $U_j$ and $\widetilde{ \nabla \xi }(x){:}{=}0$ if $x\not \in \cup _{j}U_j$. Of course, we can set $\widetilde{ \nabla \xi }(x)$ equal to an arbitrary fixed vector in ${\mathbb {R}}^d$ for $x\not \in \cup _{j}U_j$.

Since the boundary $\partial U_j$ is Lipshitz regular it has zero *d*-dimensional Lebesgue measure. By definition of the distributional derivative, we get

A.2 $$\begin{aligned} \langle \nabla \xi ,\varphi \rangle = -\langle \xi ,\nabla \varphi \rangle = - \sum _{j=1}^k \int _{U_j} \overline{\xi } \nabla \varphi \, \textrm{d}x = \sum _{j=1}^k\left( \int _{U_j} \overline{{\nabla \xi }} \varphi \, d x - \int _{\partial U_j} \overline{\xi } \varphi \nu _j\textrm{d}H^{d-1} \right) \, \end{aligned}$$

for any test function $\varphi \in \mathcal {C}^\infty _0({\mathbb {R}}^d)$, where the last equality follows from the weak form of Gauß’s theorem, see [5, Theorem A6.8] or [14, Theorem 1 in Chapter 5.8], $H^{d-1}$ is d-1 dimensional Hausdorff measure, and $\nu _j$ is the outer normal, which is well defined $H^{d-1}$ almost everywhere on $\partial U_j$, see [5, Section 6.5] or [14, Section 5.1].

Since in the sum over *j* each part of the boundaries $\partial U_j$ comes in pairs with *opposite* direction of their corresponding normals and the integrand $\overline{\xi }\varphi$ is the same on each of these pairs, we have

A.3 $$\begin{aligned} \sum _{j=1}^k \int _{\partial U_j} \overline{\xi } \varphi \nu _j\textrm{d}H^{d-1} =0\, . \end{aligned}$$

Thus

A.4 $$\begin{aligned} - \langle \xi ,\nabla \varphi \rangle = \sum _{j=1}^k \int _{U_j} \overline{{\nabla \xi }} \varphi \, d x = \langle \widetilde{\nabla \xi },\varphi \rangle \end{aligned}$$

for any test function $\varphi \in \mathcal {C}^\infty _0({\mathbb {R}}^d)$, so $\xi \in W^{1,\infty }({\mathbb {R}}^d)$.

Now we check that $\xi :\mathcal {D}(P)\rightarrow \mathcal {D}(P)$: Clearly, since ξ is bounded, $\Vert \xi \varphi \Vert \le \Vert \xi \Vert _\infty \Vert \varphi \Vert$. Since ξ has weak derivative in $W^{1,\infty }$ we have

A.5 $$\begin{aligned} \nabla (\xi \varphi ) = (\nabla \xi )\varphi + \xi \nabla \varphi \end{aligned}$$

for all $\varphi \in \mathcal {D}(P)$. Thus

$$\begin{aligned} \Vert P(\xi \varphi )\Vert&\le \Vert (\nabla \xi )\varphi \Vert + \Vert \xi P\varphi \Vert \le \Vert \nabla \xi \Vert _\infty \Vert \varphi \Vert +\Vert \xi \Vert _\infty \Vert P\varphi \Vert \\&\le (\Vert \nabla \xi \Vert _\infty +\Vert \xi \Vert _\infty )\Vert \varphi \Vert _{H^1} \end{aligned}$$

which shows that the multiplication operator $\xi :\mathcal {D}(P)\rightarrow \mathcal {D}(P)$ is bounded.

This argument shows also that $\xi ^2: \mathcal {D}(P)\rightarrow \mathcal {D}(P)$ is bounded, since replacing ξ by $\xi ^2$ in the argument leading to (A.4) shows $\xi ^2\in W^{1,\infty }({\mathbb {R}}^d)$ as soon as ξ is bounded and continuous, with bounded weak derivatives on $U_j$, since on each component $U_j$ we clearly have $\nabla (\xi ^2) = 2\xi \nabla \xi \in W^{1,\infty }(U_j)$. Thus, by what we already proved, $\xi ^2\in W^{1,\infty }({\mathbb {R}}^d)$ and $\xi ^2: \mathcal {D}(P)\rightarrow \mathcal {D}(P)$ is bounded. □

### Remark A.2

As the proof shows, the continuity assumption on ξ can be weakened to ξ is continuous in a neighborhood of the boundary set $\cup _j\partial U_j$ and this can also be further weakened using the appropriate notion of traces, see, e.g., [5, Section A.6]. Furthermore, the natural regularity assumption on the boundary of $U_j$ is that $U_j$ has locally finite perimeter, see [14, Chapter 5], so that a suitable integration–by–parts formula holds.

Our version of the famous IMS localization formula is

### Lemma A.3

(IMS localization formula, quadratic form version) Let $\xi \in W^{1,\infty }({\mathbb {R}}^d)$ be real–valued. Then for all $\varphi \in \mathcal {D}(P)=H^1({\mathbb {R}}^d)$ we have

A.6 $$\begin{aligned} \textrm{Re}\langle \nabla (\xi ^2\varphi ), \nabla \varphi \rangle = \langle \nabla (\xi \varphi ), \nabla (\xi \varphi )\rangle - \langle \varphi , |\nabla \xi |^2 \varphi \rangle \, . \end{aligned}$$

Moreover, if ρ and *f* are real–valued, $\rho \in \mathcal {D}(P)=H^1({\mathbb {R}}^d)$ and $f\in H^1_{loc}({\mathbb {R}}^d)$ with ρf and $\rho ^2 f\in H^1({\mathbb {R}}^d)$, and ρ having compact support, then

A.7 $$\begin{aligned} \langle \nabla (\rho ^2 f), \nabla f\rangle = \langle \nabla (\rho f), \nabla (\rho f)\rangle - \langle f, |\nabla \rho |^2 f\rangle \, . \end{aligned}$$

In particular, for a Schrödinger operator $H= P^2 +V$, defined in the sense of quadratic forms with a local potential *V*, we have, in the sense of quadratic forms,

A.8 $$\begin{aligned} \textrm{Re}\langle \xi ^2\varphi , H\varphi \rangle = \langle \xi \varphi , H \xi \varphi \rangle - \langle \varphi , |\nabla \xi |^2 \varphi \rangle \, . \end{aligned}$$

for any real–valued $\xi \in W^{1,\infty }({\mathbb {R}}^d)$ and any $\varphi \in \mathcal {D}(P)= H^1({\mathbb {R}}^d)$ and

A.9 $$\begin{aligned} \langle \rho ^2 f, H f\rangle = \langle \rho f, H \rho f\rangle - \langle f, |\nabla \rho |^2 f\rangle \, . \end{aligned}$$

for any real-valued ρ and *f* with $\rho \in \mathcal {D}(P)=H^1({\mathbb {R}}^d)$, $f\in H^1_{loc}({\mathbb {R}}^d)$, ρf, and $\rho ^2 f\in H^1({\mathbb {R}}^d)$, and ρ having compact support.

### Remark A.4

A natural choice for ξ is $\xi ^2=1= \sum _j\chi _j^2$ for suitable “partition of unity" given by cut–off functions $\chi _j$. A second choice, which is useful to derive weighted $L^2$ bounds, is given by an exponentially weighted function $\xi = \chi e^F$ for some (bounded) function *F*. Our version of the IMS localization formula allows us to have rather minimal, weak regularity properties on the involved functions $\chi _j$, χ, and *F*, which is very convenient for the proof of our upper bounds. The last version (A.9) is needed in the proof of Agmon’s subharmonic comparison principle.

### Proof

Lemma A.1 shows that $\xi ^2\varphi$ and $\xi \varphi$ are in $H^1({\mathbb {R}}^d)$ as soon as φ is. So both sides of (A.6) are well defined. Given $\varphi \in H^1$ and using that ξ is real-valued, a short calculation reveals

$$\begin{aligned} \textrm{Re}\langle \nabla (\xi ^2\varphi ), \nabla \varphi \rangle&= \textrm{Re}\langle \xi \nabla (\xi \varphi )+ (\nabla \xi )\xi \varphi ,\nabla \varphi \rangle = \textrm{Re}\langle \nabla (\xi \varphi ),\xi \nabla \varphi \rangle + \textrm{Re}\langle (\nabla \xi )\varphi ,\xi \nabla \varphi \rangle \\&= \textrm{Re}\langle \nabla (\xi \varphi ),\nabla (\xi \varphi )-(\nabla \xi )\varphi \rangle + \textrm{Re}\langle (\nabla \xi )\varphi ,\xi \nabla \varphi \rangle \\&= \langle \nabla (\xi \varphi ),\nabla (\xi \varphi )\rangle - \textrm{Re}\langle (\nabla \xi )\varphi + \xi \nabla \varphi , (\nabla \xi )\varphi \rangle + \textrm{Re}\langle (\nabla \xi )\varphi ,\xi \nabla \varphi \rangle \\&= \langle \nabla (\xi \varphi ),\nabla (\xi \varphi )\rangle - \langle (\nabla \xi )\varphi ,(\nabla \xi )\varphi \rangle \end{aligned}$$

since also $\textrm{Re}\langle (\nabla \xi )\varphi ,\xi \nabla \varphi \rangle = \textrm{Re}\langle \xi \nabla \varphi , (\nabla \xi )\varphi \rangle$. Thus (A.6) holds.

The formula (A.8) follows immediately from (A.6) since the potential *V* is a local multiplication operator, hence we have $\langle \xi ^2\varphi , V\varphi \rangle = \langle \xi \varphi , V\xi \varphi \rangle$ for the quadratic form.

Lastly, if $\rho \in H^1({\mathbb {R}}^d)$ and $f\in H^1_{loc}({\mathbb {R}}^d)$ with $\rho f, \rho ^2 f\in H^1({\mathbb {R}}^d)$ then their distributional derivatives satisfy $\nabla (\rho ^2 f) = \rho \nabla (\rho f) + \rho f \nabla \rho \in L^1({\mathbb {R}}^d)$ and

A.10 $$\begin{aligned} \begin{aligned} \nabla (\rho ^2 f) \nabla f&= (\rho \nabla (\rho f)+ \rho f \nabla \rho ) \nabla f = |\nabla (\rho f)|^2 - \nabla (\rho f) \rho \nabla f + \rho f \nabla \rho \nabla f \\&= |\nabla (\rho f)|^2 - f^2 |\nabla \rho |^2 \, \end{aligned} \end{aligned}$$

pointwise almost everywhere. By assumption, $\rho ^2 f$ and $\rho f\in H^1({\mathbb {R}}^d)$. If ρ has compact support, then also $\rho ^2 f$, ρf and their derivatives have compact support, so $\nabla (\rho ^2 f)\nabla f$ is integrable. Together with (A.10), this shows that $f^2 |\nabla \rho |^2$ is integrable. Integrating (A.10) yields (A.7). Moreover, for a local potential *V* we have $\rho ^2 f V f = V (\rho f)^2$ for real–valued functions ρ,f. Since by assumption $\rho f\in H^1({\mathbb {R}}^d)$, this together with the potential being relatively form small w.r.t. the kinetic energy $P^2$ and (A.7) proves the last claim (A.9). □

## Sub– and Supersolution Bounds a la Agmon

Let H=-Δ+V be a Schrödinger operator defined by the quadratic form

B.1 $$\begin{aligned} \langle \varphi , H\psi \rangle {:}{=}\langle \nabla \varphi , \nabla \psi \rangle + \langle \varphi , V\psi \rangle \end{aligned}$$

for $\varphi , \psi \in H^1({\mathbb {R}}^d)$ and an infinitesimally form small perturbation by a real-valued function *V*. Here we define $\langle \varphi , V\psi \rangle = \langle {\text {sgn}}(V) |V|^{1/2}\varphi , |V|^{1/2}\psi \rangle$ where ${\text {sgn}}(t)= t/|t|$ when t≠0 and ${\text {sgn}}(0){:}{=}0$.

### Definition B.1

*(Sub– and supersolutions in the quadratic form sense a la Agmon)* Let Ω be an open subset of ${\mathbb {R}}^d$. Then a real-valued function $\psi \in H^1_{\text {loc}}(\Omega )$ is a *supersolution* of *H* at energy $E\in {\mathbb {R}}$ in Ω if

B.2 $$\begin{aligned} \langle \varphi , (H-E)\psi \rangle \ge 0 \end{aligned}$$

and ψ is a *subsolution* if

B.3 $$\begin{aligned} \langle \varphi , (H-E)\psi \rangle \le 0 \end{aligned}$$

for all non-negative $\varphi \in \mathcal {C}^\infty _0(\Omega )$.

Of course, we can also take any non–negative $\varphi \in H^1({\mathbb {R}}^d)$ with compact support in (B.2) and (B.3). For our slight extension of a beautiful result of Agmon [2] we need one more notation.

### Definition B.2

Let $\Omega \subset {\mathbb {R}}^d$ be an open set with boundary $\partial \Omega$. We call a closed set $\widetilde{\partial \Omega }$ an (inner) boundary layer of Ω if it is a subset of the closure of Ω which contains the boundary $\partial \Omega$ of Ω in such a way that $\Omega \setminus \widetilde{\partial \Omega }$ has locally positive distance from the boundary $\partial \Omega$. More precisely, for any compact set $K\subset {\mathbb {R}}^d$ we have

B.4 $$\begin{aligned} \textrm{dist}( \partial \Omega , (K\cap \Omega )\setminus \widetilde{\partial \Omega }) >0 \, . \end{aligned}$$

In other words, a boundary layer $\widetilde{\partial \Omega }$ provides locally a safety distance to the boundary: For any R>0 there exist δ>0 such that

$$\begin{aligned} \textrm{dist}(x,\partial \Omega ) \ge \delta \end{aligned}$$

*uniformly* in $x\in \Omega \setminus \widetilde{\partial \Omega }$ with |x|≤R.

### Theorem B.3

Let Ω be an open subset of ${\mathbb {R}}^d$, *f* a positive supersolution which is bounded away from zero, and *g* a subsolution of *H* at energy *E* in Ω. Assume that for some λ>1

B.5 $$\begin{aligned} \liminf _{L\rightarrow \infty } L^{-2}\int _{\Omega \cap \{ L\le |x|\le \lambda L \}} g_+^2 \, dx =0\, \end{aligned}$$

where $g_+=\sup (g,0)$ is the positive part of *g*, and

B.6 $$\begin{aligned} f\ge g \quad \text { for {(almost)} all } x\in \widetilde{\partial \Omega } \end{aligned}$$

where $\widetilde{\partial \Omega }$ is an (inner) boundary layer of Ω. Then f≥g almost everywhere in Ω.

### Remark B.4

We would like to stress that this is basically Theorem 2.7 in [2]. It is a slight extension of Agmon’s result, since he considers neighborhoods of infinity, i.e., complements of compact subsets of ${\mathbb {R}}^d$. We need to work with more general unbounded domains which contain infinity but are not necessarily neighborhoods of infinity.

By f>0 being bounded away from zero we mean that for any compact subset $K\subset {\mathbb {R}}^d$ there exist a constant $c_K>0$ such that $f(x)\ge c_K>0$ for all x∈K.

### Proof

We give a sketch of the proof, for the convenience of the reader. Since *f* is a positive supersolution and *g* is a subsolution in Ω, the function

B.7 $$\begin{aligned} u{:}{=}(g-f)_+ \end{aligned}$$

is a subsolution of *H* at energy *E* in Ω, see [2, 2.9 Lemma] whose proof also shows that one only needs $V\in L^1_{\text {loc}}({\mathbb {R}}^d)$ for this. So for any real-valued $\xi \in \mathcal {C}^\infty _0(\Omega )$ we can choose $\varphi = \xi ^2u$ as a testfunction in the IMS localization formula (A.6) to get

$$\begin{aligned} \langle \xi u, (H-E)\xi u\rangle -\langle u, |\nabla \xi |^2 u\rangle = \textrm{Re}\langle \xi ^2 u, (H-E)u\rangle =\langle \xi ^2 u, (H-E)u\rangle \le 0\, . \end{aligned}$$

That is, we have

B.8 $$\begin{aligned} \langle \xi u, (H-E)\xi u\rangle \le \langle u, |\nabla \xi |^2 u\rangle \quad \text {for all } \xi \in \mathcal {C}^\infty _0(\Omega )\, . \end{aligned}$$

Extend *u* by zero to $\Omega ^c$, by a slight abuse of notation, we continue to use *u* for this extension. Since *u* vanishes on the boundary layer $\widetilde{\partial \Omega }$, i.e, within a safety–distance to the boundary $\partial \Omega$, this extension of *u* is in the Sobolev space $H^1({\mathbb {R}}^d)$. Moreover, thanks to *u* being zero on the boundary layer $\widetilde{\partial \Omega }$, it is easy to see that (B.8) continues to hold for all real-valued $\xi \in \mathcal {C}^\infty _0({\mathbb {R}}^d)$, since the both sides of the inequality (B.8) depend only on the values of ξ on the support of *u*. So

B.9 $$\begin{aligned} \langle \xi u, (H-E)\xi u\rangle \le \langle u, |\nabla \xi |^2 u\rangle \quad \text {for all } \xi \in \mathcal {C}_0^\infty ({\mathbb {R}}^d)\, . \end{aligned}$$

By assumption, f>0 is a supersolution of *H* at energy *E* in Ω. Thus for any real-valued non–negative $\xi \in \mathcal {C}^\infty _0({\mathbb {R}}^d)$ we can choose $\varphi = \xi ^2 f$, with $\rho =\xi /f$, as a positive test function with support in Ω. It is straightforward to check that the conditions of (A.9) are satisfied since *f* is locally bounded away from zero. So from the the definition of a supersolution and (A.9) we get

B.10 $$\begin{aligned} 0\le \langle \varphi , (H-E) f \rangle = \langle \rho ^2 f, (H-E)f\rangle = \langle \xi , (H-E)\xi \rangle - \langle f, \big | \nabla \Big ( \frac{\xi }{f} \Big ) \big |^2 f\rangle \, . \end{aligned}$$

Now replace ξ by $\xi g_n$ with $0\le g_n\in \mathcal {C}^\infty _0({\mathbb {R}}^d)$ converging to $u=(f-g)_+$ in $H^1_{loc}({\mathbb {R}}^d)$. Then (B.10) shows that

B.11 $$\begin{aligned} \begin{aligned} \left\langle f, \big | \nabla \Big ( \frac{\xi u}{f} \Big ) \big |^2 f\right\rangle&= \lim _{n\rightarrow \infty } \left\langle f, \big | \nabla \Big ( \frac{\xi g_n}{f} \Big ) \big |^2 f\right\rangle \\&\le \lim _{n\rightarrow \infty } \langle \xi g_n, (H-E)\xi g_n \rangle = \langle \xi u, (H-E)\xi u \rangle \, . \end{aligned} \end{aligned}$$

Together with (B.9) this yields

B.12 $$\begin{aligned} \left\langle f, \big | \nabla \Big ( \frac{\xi u}{f} \Big ) \big |^2 f\right\rangle \le \langle u, |\nabla \xi |^2 u\rangle \quad \text {for all } 0\le \xi \in \mathcal {C}_0^\infty ({\mathbb {R}}^d)\, . \end{aligned}$$

Now pick $\chi \in \mathcal {C}^\infty _0({\mathbb {R}}^d)$ with $0\le \chi \le 1$, χ(x)=1 for |x|≤1, χ(x)=0 for $|x|\ge \lambda >1$, and use the scaled version $\xi (x)=\xi _L(x)=\chi (x/L)$ in (B.12). Since $\xi _L\rightarrow 1$ pointwise as $L\rightarrow \infty$, Fatou’s lemma and (B.12) imply

B.13 $$\begin{aligned} \begin{aligned} \left\langle f, \big | \nabla \Big ( \frac{u}{f} \Big ) \Big |^2 f\right\rangle&\le \liminf _{L\rightarrow \infty } \left\langle f, \big | \nabla \Big ( \frac{\xi _L u}{f} \Big ) \big |^2 f\right\rangle \le \liminf _{L\rightarrow \infty }\langle u, |\nabla \xi _L|^2 u\rangle \\&\le \liminf _{L\rightarrow \infty }\int _\Omega |\nabla \xi _L|^2 g_+^2\, dx =0 \end{aligned} \end{aligned}$$

where in the second inequality we used $u = (g-f)_+\le g_+$, which follows from f>0 in Ω. The last equality in (B.13) is due to the bound $\nabla \xi _L\le \Vert \nabla \chi \Vert _\infty ^2 L^{-2} \textbf{1}_{\{L\le |x|\le \lambda L\}}$ and assumption (B.5).

Clearly, (B.13) implies that u=cf in Ω for some constant *c*. Since f>0 in Ω and u=0 on the boundary layer $\widetilde{\partial \Omega }$, we must have c=0. Thus $u=(g-f)_+=0$ almost everywhere in Ω, which proves the lemma. □

## A Tightness Argument

In this section we show how one can prove a simple tightness argument for weakly convergent sequences in $L^2({\mathbb {R}}^d)$ from [27]. Of course, such results are well-known using, for example, the locally compact embedding of the Sobolev space $H^1$ into suitable $L^p$ spaces. Our point is that one can work with a pure $L^2$–space version. The tightness argument we use is based on the following equivalence.

### Theorem C.3

([27]) Let $(\psi _n)_{n\in \mathbb {N}}$ be a sequence in $L^2(\mathbb {R}^d)$ and $(\widehat{\psi }_n)_{n\in {\mathbb {N}}}$ the sequence of Fourier transforms. Then the following are equivalent.

1. The sequence $(\psi _n)_{n\in \mathbb {N}}$ is converging strongly,
2. The sequence $(\psi _n)_{n\in \mathbb {N}}$ is converging weakly and satisfies
  C.1 $$\begin{aligned} \lim _{R\rightarrow \infty }\limsup _{n\rightarrow \infty }\int _{|x|>R}|\psi _n(x)|^2\text {d}x&=0\,,\end{aligned}$$
  C.2 $$\begin{aligned} \lim _{L\rightarrow \infty }\limsup _{n\rightarrow \infty }\int _{|k|>L}|\hat{\psi }_n(k)|^2\text {d}k&=0\, , \end{aligned}$$
3. The sequence $(\psi _n)_{n\in \mathbb {N}}$ is converging weakly and there exist functions H,F≥0 with $\lim _{|x|\rightarrow \infty }H(x)=\infty =\lim _{|k|\rightarrow \infty }F(k)$ such that
  C.3 $$\begin{aligned} \limsup _{n\rightarrow \infty }\int _{\mathbb {R}^d}H(x)|\psi _n(x)|^2\text {d}x&<\infty \,,\end{aligned}$$
  C.4 $$\begin{aligned} \limsup _{n\rightarrow \infty }\int _{\mathbb {R}^d}F(k)|\hat{\psi }_n(k)|^2\text {d}k&<\infty \,. \end{aligned}$$

### Remark C.2

It is straightforward to see that conditions (C.1) and (C.3), respectively, conditions (C.2) and (C.4), are equivalent.

### Proof

For the convenience of the reader, we give a sketch of the proof. The equivalence of part and of Theorem C.3 was already shown in [27]. The easy argument is as follows: It is clear that part implies part . For the converse assume that $\psi _n$ converges weakly to zero in $L^2$, without loss of generality. Let $P_L=\textbf{1}_{\{|P|\le L\}}$ be a Fourier cutoff onto momenta $|\eta |\le L$ in Fourier space and $Q_R=\textbf{1}_{\{|x|\le R\}}$ be a cutoff in *x*-space. Then it is easy to see that $P_L Q_R$ has a square integrable kernel, i.e., it is a Hilbert–Schmidt operator on $L^2$, hence compact, [40]. Since

$$\begin{aligned} \psi _n = P_L\psi _n + (1-P_L)\psi _n = P_L Q_R\psi _n + P_L(1-Q_R)\psi _n + (1-P_L)\psi _n \end{aligned}$$

and $P_L$ is an orthogonal projection we get

C.5 $$\begin{aligned} \limsup _{n\rightarrow \infty }\Vert \psi _n\Vert \le \limsup _{n\rightarrow \infty } \Vert (1-Q_R)\psi _n\Vert + \limsup _{n\rightarrow \infty }\Vert (1-P_L)\psi _n\Vert \end{aligned}$$

using that $\lim _{n\rightarrow \infty } \Vert P_L Q_R \psi _n\Vert =0$, by compactness of $P_L Q_R$ for all fixed *L*, *R*. Now simply note that the two terms on the right hand side of (C.5) go to zero as $R,L\rightarrow \infty$ due to (C.1), respectively (C.2).

Clearly part implies part using the Chebycheff–Markov inequality. For the converse assume that (C.3) holds and take an increasing sequence of radii $R_k\rightarrow \infty$ with

C.6 $$\begin{aligned} \limsup _{n\rightarrow \infty }\int _{|x|>R_k}|\psi _n(x)|^2\text {d}x\le 2^{-2k} \ \end{aligned}$$

for all $k\in {\mathbb {N}}$. Setting

C.7 $$\begin{aligned} H(x)\,= \sum _{k: R_k< |x|}^\infty 2^k \end{aligned}$$

we get

$$\begin{aligned} \limsup _{n\rightarrow \infty } \int H(x) |\psi _n(x)|^2\, dx \le \sum _{k=0}^\infty 2^k \limsup _{n\rightarrow \infty } \int _{|x|>R_k} |\psi _n(x)|^2\, dx \le \sum _{k=0}^\infty 2^{-k} =1. \end{aligned}$$

i.e., (C.3) holds. Similarly one shows that (C.2) implies (C.4). □

## Basic Properties of the Ground State

In this appendix we gather some basic properties of the ground state and the ground state energy. We call $E_U=\inf \sigma (H_U)$ the ground state energy, although, technically, it is only the ground state energy when a ground state exists.

A result of Lieb [33], based on a clever idea of Benguria, but now applied for the case that the electron–electron repulsion term contains a coupling constant *U*, shows that a single atom with nuclear charge *Z* can only bind

D.1 $$\begin{aligned} N< \frac{2Z}{U} +1 \end{aligned}$$

particles, independent of the statistics of the particles. That is, if the ground state energy is strictly below the essential spectrum, then (D.1) holds. For N=2 and Z=1 this shows that $E_U<-1/4= {\sigma _{\text {ess}}}(H_U)$ implies U<2. Thus, for the operator given by (1.1), the critical coupling $U_c$ is bounded by

D.2 $$\begin{aligned} 1<U_c\le 2 \, . \end{aligned}$$

These simple rigorous bounds give a rough, but not bad, estimate for the critical coupling $U_c$. As discussed in the introduction, calculations by quantum chemists predict $U_c\sim 1.097 66$, see [7], and even pushed the calculation to $U_c\simeq 1,097\, 660\, 833\, 738 56$ in [13]. Bethe showed in [9] with the help of a variational calculation that hydrogen can bind two electrons, hence clearly $U_c>1$. Moreover, Hill showed that even for finite nuclear mass *M* the $H^-$ quantum system has a ground state in the center–of–mass frame for some U>1 as long as the mass ration m/M≤0.2101.

### Proposition D.1

Given a helium-type operator $H_U$, with infinite nuclear mass let $E_U=\inf \sigma (H_U)$ be its ground state energy. Then

1. $U\mapsto E_U$ is concave and continuous. It is strictly increasing on $[0,U_c]$, constant on $[U_c,\infty )$, and
  D.3 $$\begin{aligned} E_U<-\frac{1}{4} \quad \text {for }0\le U<U_c,\quad E_{U_c}=-\frac{1}{4} \, . \end{aligned}$$
  Moreover, $E_U$ is differentiable in $U<U_c$ with a positive derivative. At $U_c$ its left derivative exists and is positive.
2. At critical coupling the energy $E_{U_c}=-1/4$ is a simple eigenvalue at the edge of the essential spectrum. The corresponding ground state $\psi _c=\psi _{U_c}\in L^2$ is unique and positive up to a global phase factor.
3. Choosing all ground states $\psi _U$ for $0\le U\le U_c$ normalized, i.e., $\Vert \psi _U\Vert _2=1$, and positive, the map $[0,U_c]\ni U\mapsto \psi _U$ is continuous in $H^1$ and in $L^q$ for all $2\le q\le \infty$. Moreover, there exist a constant C<∞ such that for any $2\le q\le \infty$ we have
  D.4 $$\begin{aligned} \sup _{0\le U\le U_c}\Vert \psi _U\Vert _q \le C \, . \end{aligned}$$
4. For any normalized positive ground state $\psi _U$ with $\Vert \psi _U\Vert _2=1$ and any compact set $K\subset {\mathbb {R}}^6$ there exists a constant $C_K>0$ such that
  D.5 $$\begin{aligned} \inf _{0\le U\le U_c} \inf _{x\in K} \psi _U(x)\ge C_K\, . \end{aligned}$$

### Proof

Let $H_0$ be the operator $H_U$ with U=0. Since $H_U= H_{0}+ \frac{U}{|x_1-x_2|}$ is linear in the perturbation, one sees that $E_U= \inf _{\Vert \psi \Vert =1} \langle \psi , H_U\psi \rangle$ is an infimum of linear functions, hence concave. Since $-\infty<E_U<\infty$ for all $U\in {\mathbb {R}}$, it is continuous.

At critical coupling and above, the energy $E_{U}$ is equal to the infimum of the essential spectrum, which by the HVZ theorem is given by the ground state energy of hydrogen. Hence $E_{U}= -1/4$ for all $U\ge U_c$.

When $U<U_c$, perturbation theory [36] applies. First order perturbation theory, the so-called Feynman–Hellmann formula, (see Remark (3.5.18) in [44]), shows that

D.6 $$\begin{aligned} \partial _U E_U = \langle \psi _U, \frac{dH_U}{dU}\psi _U \rangle = \langle \psi _U, \frac{1}{|x_1-x_2|}\psi _U \rangle >0 \end{aligned}$$

for all $U<U_c$. Moreover, this formula also holds, as soon as a normalizable ground $\psi _U$ state exists, for critical coupling $U=U_c$, when one replaces the derivative $\partial _U E_U$ with the left derivative $\partial _U^-E_U= \lim _{U\nearrow U_c} \frac{E_U-E_{U_c}}{U-U_c}$, see [38]. This proves claim D.1., once a normalizable ground state exists at critical coupling. This is known from [15, 23] but we will give an alternative proof for this, which uses the isotropic upper bound from Theorem 4.1.

The Coulomb potential $V_U$ is in the so–called Kato–class, for a definition see [4, 11, 39], for any coupling $U\in {\mathbb {R}}$. Moreover, $V_U\ge V_0$ for all U≥0, so [39, Theorem B.1.1] and monotonicity of the Schrödinger semigroup in the potential gives

$$\begin{aligned} \Vert \psi _U\Vert _p = \Vert \textrm{e}^{tE_U} e^{-tH_U}\psi _U\Vert _p \le e^{tE_U}\Vert e^{-tH_U}\Vert _{2\rightarrow p }\Vert \psi _U\Vert \le e^{tE_U}\Vert e^{-tH_0}\Vert _{2\rightarrow p }\Vert \psi _U\Vert \, . \end{aligned}$$

So for fixed t>0 and with $C_p= e^{tE_U}\Vert e^{-tH_0}\Vert _{2\rightarrow p }$ we have $\Vert \psi _U\Vert _p\le C_p\Vert \psi _U\Vert$ for all $2\le p\le \infty$. The Riesz–Thorin interpolation theorem also shows $C_p\le C_2^{\Theta } C_\infty ^{1-\Theta }$ for any $1/p = \Theta /2 + (1-\Theta )/\infty = \Theta /2$ and $0\le \Theta \le 1$. Hence $C_p\le \max (C_2,C_\infty ){=}{:}C$ and we arrive at

D.7 $$\begin{aligned} \sup _{0\le U<U_c}\Vert \psi _U\Vert _p\le C \end{aligned}$$

when $$\Vert \psi _U\Vert =1$$ for $$0\le U <U_c$$.

Take a sequence $1<U_n<U_c$ which converges to $U_c$ and consider the corresponding sequence of normalized bound states $\psi _n$ of $H_n=H_{U_n}$ with energies $E_n<-1/4$. The Coulomb potential $V_U$ is relatively form bounded with respect to the Laplacian -Δ in ${\mathbb {R}}^6$ with relative form bound zero, and thus

D.8 $$\begin{aligned} |\langle \psi , V_U\psi \rangle |\le \delta \Vert \nabla \psi \Vert ^2 + b_\delta \Vert \psi \Vert ^2, \end{aligned}$$

and, in particular,

D.9 $$\begin{aligned} |\langle \psi , \frac{1}{|x_1-x_2|}\psi \rangle |\le \delta \Vert \nabla \psi \Vert ^2 + b_\delta \Vert \psi \Vert ^2 \end{aligned}$$

for all 0<δ<1 and some $b_\delta >0$ uniformly in $0\le U\le U_c$ for all $\psi \in H^1({\mathbb {R}}^6)$. Thus, for $\psi _n$ we get

$$\begin{aligned} E_n= \langle \psi _n, H_n \psi _n\rangle = \langle \nabla \psi _n,\nabla \psi _n\rangle + \langle \psi _n, V_U\psi _n \rangle \ge (1-\delta )\Vert \nabla \psi _n\Vert ^2 -b_\delta \Vert \psi _n\Vert ^2 \end{aligned}$$

which implies that

$$\begin{aligned} \Vert \nabla \psi _n\Vert ^2 \le \frac{b_\delta }{1-\delta } \Vert \psi _n\Vert ^2, \end{aligned}$$

since the energy $E_n$ of the minimizing sequence is bounded. Therefore,

$$\begin{aligned} \Vert \psi _n\Vert _{H^1({\mathbb {R}}^6)}^2 = \Vert \psi _n\Vert ^2 + \Vert \nabla \psi _n\Vert ^2 \le \frac{b_\delta }{1-\delta } \Vert \psi _n\Vert ^2 +\Vert \psi _n\Vert ^2 = \frac{b_\delta }{1-\delta }+1<\infty \end{aligned}$$

uniformly in $n\in {\mathbb {N}}$, since $\psi _n$ is normalized. Thus the sequence $\psi _n$ is bounded in $H^1$, hence there exists a subsequence, which we also denote by $\psi _n$, which converges weakly to some $\psi _c\in H^1({\mathbb {R}}^6)$. We claim that $\psi _c$ is a normalized ground state of $H_{U_c}$, i.e., $\Vert \psi _c\Vert =1$, $\psi _c\in H^1({\mathbb {R}}^6)$, and $\langle \varphi , H_{U_c}\psi _c\rangle = -1/4\langle \varphi ,\psi _c\rangle$ for all $\varphi \in H^1({\mathbb {R}}^6)$.

Since $\psi _n$ is a bounded sequence in $H^1({\mathbb {R}}^6)$ it is tight in momentum space,

D.10 $$\begin{aligned} \lim _{L\rightarrow \infty }\sup _{n\in {\mathbb {N}}}\int _{|\eta |\ge L} |\widehat{\psi }_n|^2\textrm{d}\eta =0\, . \end{aligned}$$

Moreover, the upper bound from Theorem 4.1, which only needs the bound (D.7) for $\psi _n=\psi _{U_n}$ with subcritical $U_n<U_c$, easily implies also tightness of the sequence $\psi _n$ in position space,

D.11 $$\begin{aligned} \lim _{R\rightarrow \infty }\sup _{n\in {\mathbb {N}}}\int _{|\eta |\ge L} |\psi _n|^2\textrm{d}\eta =0\,, \end{aligned}$$

and standard compactness results, for example Theorem C.3, show that $\psi _n$ converges to $\psi _c$ strongly in $L^2({\mathbb {R}}^6)$, so $\Vert \psi _c\Vert =\lim _{n\rightarrow \infty }\Vert \psi _n\Vert =1$.

We will show in a moment that $\psi _n$ also converges strongly in $H^1({\mathbb {R}}^6)$, the form domain of all operators $H_U$. Once this is the case a bound of the form (D.8) for some δ>0 implies $\langle \psi _c, V_{U_c}\psi _c\rangle =\lim _{n\rightarrow \infty } \langle \psi _n V_{U_n}\psi _n\rangle$. Once we have this, we also get that

D.12 $$\begin{aligned} -\frac{1}{4} \le \langle \psi _c, H_{U_c}\psi _c \rangle = \liminf _{n\rightarrow \infty } \langle \psi _n, H_{U_n}\psi _n \rangle = -\frac{1}{4} \, , \end{aligned}$$

because of the strong convergence of $\psi _n$ in $H^1({\mathbb {R}}^6)$ and $\langle \psi _n, H_{U_n}\psi _n \rangle = E_n\rightarrow -1/4$ in the limit $n\rightarrow \infty$, where we also used that $\psi _c$ is normalized and $H_{U_c}\ge -\tfrac{1}{4}$, as quadratic forms. Thus $\psi _c$ is a normalized minimizer of the energy. By the variational principle, $\psi _c$ is a weak eigenfunction of $H_{U_c}$: $\langle \varphi , H_{U_c}\psi _c\rangle = -\tfrac{1}{4}\langle \varphi ,\psi _c\rangle$ for all $\varphi \in H^1({\mathbb {R}}^6)$. The uniqueness, up to a global phase, follows from the usual arguments, [36, Theorem XII44], see also [17], since the semigroup $e^{-tH_U}$ is positivity improving, i.e. $e^{-tH_U}f>0$ if f≥0 and f≠0. This proves claim D.1., once we established the strong convergence of $\psi _n$ in $H^1({\mathbb {R}}^6)$.

To prove the strong convergence in $H^1({\mathbb {R}}^6)$ we note that

$$\begin{aligned} H_n = H_{U_c} + \frac{U_n-U_c}{|x_1-x_2|} {=}{:}H_{U_c} +W_{U_n} \end{aligned}$$

so for all $\varphi \in H^1({\mathbb {R}}^d)$ we have

$$\begin{aligned} \begin{aligned} \langle \varphi , H_{U_c} (\psi _n-\psi _m)\rangle&= \langle \varphi , H_{n} \psi _n\rangle - \langle \varphi , H_{m} \psi _m\rangle \\&\quad - \langle \varphi , W_{U_n} (\psi _n-\psi _m)\rangle + \langle \varphi , W_{U_m} (\psi _n-\psi _m)\rangle \\&= E_n\langle \varphi ,\psi _n\rangle - E_m\langle \varphi , \psi _m\rangle \\&\quad - \langle \varphi , W_{U_n} (\psi _n-\psi _m)\rangle + \langle \varphi , W_{U_m} (\psi _n-\psi _m)\rangle \\&\le \Vert \varphi \Vert \big ( |E_n|\Vert \psi _n\Vert + |E_m|\Vert \psi _m\Vert \big ) \\&\quad - \langle \varphi , W_{U_n} (\psi _n-\psi _m)\rangle + \langle \varphi , W_{U_m} (\psi _n-\psi _m)\rangle \, . \end{aligned} \end{aligned}$$

Choosing $\varphi =\psi _n-\psi _m$, and also using (D.9) to bound the terms $\langle \psi _n-\psi _m, W_U(\psi _n-\psi _m)\rangle$, and the normalization $\Vert \psi _n\Vert =1,$ we get that

D.13 $$\begin{aligned}  &   \langle \psi _n-\psi _m, H_{U_c} (\psi _n-\psi _m)\rangle \nonumber \\  &   \quad \le \big (|E_n| + |E_m|\big ) \Vert \psi _n-\psi _m\Vert \\  &   \qquad + (2U_c-U_n-U_m) \big ( \delta \Vert \nabla (\psi _n-\psi _m)\Vert ^2 + b_\delta \Vert \psi _n-\psi _m\Vert ^2 \big ) \, .\nonumber \end{aligned}$$

On the other hand, due to (D.8) we also have the lower bound

$$\begin{aligned} \begin{aligned} \langle \psi _n-\psi _m, H_{U_c} (\psi _n-\psi _m)\rangle \ge (1-\delta ) \Vert \nabla (\psi _n-\psi _m)\Vert ^2 - b_\delta \Vert \psi _n-\psi _m\Vert ^2, \end{aligned} \end{aligned}$$

and using this in (D.13) and rearranging terms one gets

$$\begin{aligned} \big (1-\delta (1+(&2U_c-U_n-U_m)\big ) \Vert \nabla (\psi _n-\psi _m)\Vert ^2 \\&\le (|E_n| + |E_m|) \Vert \psi _n-\psi _m\Vert + b_\delta (1+2U_c-U_n-U_m) \Vert \psi _n-\psi _m\Vert ^2 \, . \end{aligned}$$

Since δ<1 and $U_n,U_m\rightarrow U_c$, this shows that

D.14 $$\begin{aligned} \limsup _{n,m\rightarrow \infty } \Vert \nabla (\psi _n-\psi _m)\Vert ^2 \lesssim \limsup _{n,m\rightarrow \infty }\Vert \psi _n-\psi _m\Vert =0, \end{aligned}$$

since $\psi _n$ converges to $\psi _c$ strongly in $L^2$. Thus $\psi _n$ is also Cauchy in $H^1({\mathbb {R}}^6)$, hence it converge strongly in $H^1({\mathbb {R}}^6)$. This finishes the proof of D.1..

Having the existence of $\psi _U$ at $U=U_c$, the same argument which proved (D.7) can be used to prove (D.4). Continuity of $\psi _U$ in $U<U_c$ with respect to the $L^2$ norm follows from perturbation theory. We will give an alternative proof, which also yields continuity in $H^1$ in the whole interval of parameters *U* for which a ground state exits.

Take an arbitrary $U_0\in [0,U_c]$ and any sequence $U_n\in [0,U_c]$ converging to $U_0$. Let $U^1_j= U_{n_j}$ be an arbitrary subsequence. Then the above argument shows that this sequence is tight. Since $\Vert \psi _{U^1_j}\Vert =1$ there exists a further subsequence $U^2_k= U^1_{j_k}$ such that $\psi _{U^2_k}$ converges weakly in $L^2$ to $\psi _{U_0}$, which is the unique ground state of $H_{U_0}$. By tightness, $\psi _{U^2_k}$ converges strongly in $L^2$ and the above argument then shows that it also converges strongly in $H^1$.

Thus any subsequence of $\psi _{U_n}$ with $U_n\rightarrow U_0\in [0,U_c]$ has a further subsequence which converges to the same limit $\psi _{U_0}$, i.e., $\psi _{U_n}$ converges in $H^1$ to $\psi _{U_0}$ for any sequence $U_n\rightarrow U_0$. Hence $[0,U_c]\ni U\mapsto \psi _U$ is continuous in $H^1$.

The Coulomb potential $V_U$ is in the Kato–class and it is continuous in *U* in the Kato–norm (for definitions, see [39, Section A.2]). Thus [39, Theorem B.10.1] shows that as operators from $L^2$ to $L^q$

$$\begin{aligned} \Vert e^{-tH_U}- e^{-tH_{U_0}}\Vert _{2\rightarrow q}\rightarrow 0 \end{aligned}$$

as $$U\rightarrow U_0$$ for any fixed t>0 and $$2\le q\le \infty $$. Define $$\widetilde{H}_U= H_U-E_U$$. Then continuity of $$E_U$$ in *U* shows that also $$\Vert e^{-t\widetilde{H}_{U'}}- e^{-t\widetilde{H}_{U}}\Vert _{2\rightarrow q}\rightarrow 0$$ as $U^{\prime }\to U$ for any fixed t>0 and $$2\le q\le \infty $$. Using $$\widetilde{H}_U\psi _U=0$$, we have $$\psi _U= e^{-t\widetilde{H}_U}\psi _U$$ for all $$U\le U_c$$. Hence

$$\begin{aligned} \psi _{U'}-\psi _{U}&= e^{-t\widetilde{H}_{U'}}\psi _{U'} - e^{-t\widetilde{H}_U}\psi _U = \Big ( e^{-t\widetilde{H}_{U'}} - e^{-t\widetilde{H}_U}\Big )\psi _{U'} + e^{-t\widetilde{H}_U}\big ( \psi _{U'}-\psi _U\big ) \end{aligned}$$

and with the $L^2$ normalization $\Vert \psi _U\Vert =1$ and the $L^2$ continuity of $\psi _U$ in $U\in [0,U_c]$ we have

$$\begin{aligned} \Vert \psi _{U'}-\psi _{U}\Vert _q&\le \Vert e^{-t\widetilde{H}_{U'}} - e^{-t\widetilde{H}_U}\Vert _{2\rightarrow q} + \Vert e^{-t\widetilde{H}_U}\Vert _{2\rightarrow q} \Vert \psi _{U'}-\psi _U\Vert \rightarrow 0 \end{aligned}$$

as $U^{\prime }\to U$ for any fixed t>0 and $$2\le q\le \infty $$. This finishes the proof of claim D.1..

Let $\psi _U$ be the strictly positive ground state which, as we just proved, exists for any $U\le U_c$. Since $V_U$ is in the Kato–class, any eigenfunction of $H_U$ is continuous [39, Theorem B.3.1]. So for any compact set $K\subset {\mathbb {R}}^6$ and each $0\le U\le U_c$

D.15 $$\begin{aligned} c(K,U){:}{=}\inf _{x\in K} \psi _U(x)>0 \, . \end{aligned}$$

By continuity of the $L^q$ norms in *U*, there exists an open set $O_U\subset {\mathbb {R}}$ such that

D.16 $$\begin{aligned} \Vert \psi _{U'}-\psi _U\Vert _\infty <\frac{1}{2} c(K,U) \quad \text {for all } U'\in [0,U_c]\cap O_U\, . \end{aligned}$$

Thus

$$\begin{aligned} \inf _{x\in K} \psi _{U'}(x) \ge \inf _{x\in K} \psi _U(x) - \Vert \psi _{U'}-\psi _U\Vert _\infty \ge \frac{1}{2} c(K,U) \end{aligned}$$

for all $U'\in [0,U_c]\cap O_U$. Clearly, since $U\in O_U$, the open sets $O_U$ cover $[0,U_c]$, i.e., $[0,U_c]\subset \cup _{U\in [0,U_c]}O_U$. Since $[0,U_c]$ is compact, there exist finitely many $O_j= O_{U_j}$, j=1,…,N such that $[0,U_c]\subset \cup _{j=1}^N O_j$. So with $c_j(K)= c(K,U_j)$, we get

$$\begin{aligned} \inf _{0\le U\le U_c}\inf _{x\in K} \psi _{U}(x) \ge \frac{1}{2} \min _{j=1,\ldots , N} c_j(K){=}{:}C_K >0\, , \end{aligned}$$

which proves the last claim. □

## Footnotes

1 A nice textbook treatment of the WKB method is given in Chapter 9 of [16]. The WKB method also determines a polynomial prefactor in the asymptotic of eigenfunctions, which gives a logarithmic correction to the exponential weight *F*. The lower order correction terms we use in order to control errors are much bigger, so we ignore logarithmic corrections for this discussion and concentrate on the leading order term.

## References

[1] Agmon, S.: Lectures on exponential decay of solutions of second-order elliptic equations: bounds on eigenfunctions of -body Schrödinger operators, Mathematical Notes, vol. 29. Princeton University Press, Princeton (1982)

[2] Agmon, S.: Bounds on exponential decay of eigenfunctions of Schrödinger operators. In: Schrödinger operators (Como, 1984), Lecture Notes in Math., vol. 1159, pp. 1–38. Springer, Berlin (1985). 10.1007/BFb0080331.

[3] Ahlrichs, R.: Asymptotic behavior of atomic bound state wave functions. *J. Mathematical Phys.* **14**, 1860–1863, 1973. 10.1063/1.1666258

[4] Aizenman, M., Simon, B.: Brownian motion and Harnack inequality for Schrödinger operators. *Comm. Pure Appl. Math.* **35**(2), 209–273, 1982. 10.1002/cpa.3160350206

[5] Alt, H.W.: Linear functional analysis. 6th edition (2012).

[6] Baker, J.D., Freund, D.E., Hill, R.N., Morgan, J.D.: Radius of convergence and analytic behavior of the expansion. *Phys. Rev. A* **41**, 1247–1273, 1990. 10.1103/PhysRevA.41.1247 9903218 10.1103/physreva.41.1247

[7] Baker, J.D., Freund, D.E., Hill, R.N., Morgan, J.D., III.: Radius of convergence and analytic behavior of the 1/z expansion. *Phys. Rev. A* **41**(3), 1247, 1990 9903218 10.1103/physreva.41.1247

[8] Barth, S., Bitter, A., Vugalter, S.: Decay properties of zero-energy resonances of multi-particle Schrödinger operators and why the Efimov effect does not exist for systems of particles. Preprint arXiv:1910.04139 p. 36 (2019).

[9] Bethe, H.A.: Berechnung der Elektronenaffinität des Wasserstoffs. *Zeitschrift für Physik* **57**(11–12), 815–821, 1929

[10] Combes, J.M., Thomas, L.: Asymptotic behaviour of eigenfunctions for multiparticle Schrödinger operators. *Comm. Math. Phys*. **34**, 251–270 (1973). http://projecteuclid.org/euclid.cmp/1103859473.

[11] Cycon, H.L., Froese, R.G., Kirsch, W., Simon, B.: Schrödinger operators with application to quantum mechanics and global geometry. Texts and Monographs in Physics, study. Springer-Verlag, Berlin (1987)

[12] Deift, P., Hunziker, W., Simon, B., Vock, E.: Pointwise bounds on eigenfunctions and wave packets in -body quantum systems. *IV. Comm. Math. Phys*. **64**(1), 1–34 (1978/79). http://projecteuclid.org/euclid.cmp/1103904619.

[13] Estienne, C.S., Busuttil, M., Moini, A., Drake, G.W.F.: Critical nuclear charge for two-electron atoms. *Phys. Rev. Lett.* **112**, 173001, 2014. 10.1103/PhysRevLett.112.173001 24836241 10.1103/PhysRevLett.112.173001

[14] Evans, L.C., Gariepy, R.F.: Measure theory and fine properties of functions. Studies in Advanced Mathematics. CRC Press, Boca Raton, FL (1992)

[15] Frank, R.L., Lieb, E.H., Seiringer, R.: Binding of polarons and atoms at threshold. *Comm. Math. Phys.* **313**(2), 405–424, 2012. 10.1007/s00220-012-1436-9

[16] Galindo, A., Pascual, P.: Quantum mechanics. II. Texts and Monographs in Physics. Springer-Verlag, Berlin (1991). 10.1007/978-3-642-84129-3. Translated from the second Spanish edition by L. Alvarez-Gaumé.

[17] Goelden, H.W.: On non-degeneracy of the ground state of Schrödinger operators. *Math. Z.* **155**(3), 239–247, 1977. 10.1007/BF02028443

[18] Grabowski, P.E., Burke, K.: Quantum critical benchmark for electronic structure theory. *Phys. Rev. A* **91**, 032501, 2015. 10.1103/PhysRevA.91.032501

[19] Gridnev, D.K.: Bound states at threshold resulting from Coulomb repulsion. *J. Math. Phys.* **53**(10), 102108, 16 (2012). 10.1063/1.4758076.

[20] Griesemer, M.: Exponential decay and ionization thresholds in non-relativistic quantum electrodynamics. *J. Funct. Anal.* **210**(2), 321–340, 2004. 10.1016/j.jfa.2003.06.001

[21] Hill, R.N.: Proof that the ion has only one bound state. *Phys. Rev. Lett.* **38**, 643–646, 1977. 10.1103/PhysRevLett.38.643

[22] Hill, R.N.: Proof that the ion has only one bound state details and extension to finite nuclear mass. *J. Math. Phys.* **18**(12), 2316–2330, 1977. 10.1063/1.523241

[23] Hoffmann-Ostenhof, M., Hoffmann-Ostenhof, T., Simon, B.: A multiparticle Coulomb system with bound state at threshold. *J. Phys. A* **16**(6), 1125–1131 (1983). http://stacks.iop.org/0305-4470/16/1125.

[24] Hoffmann-Ostenhof, T.: A lower bound to the decay of ground states of two electron atoms. *Phys. Lett.* **77A**, 140–142, 1980 http://www.sciencedirect.com/science/article/pii/0375960180901735

[25] Hoffmann-Ostenhof, T., Hoffmann-Ostenhof, M., Ahlrichs, R.: “Schrödinger inequalities’’ and asymptotic behavior of many-electron densities. *Phys. Rev. A* **18**, 328–334, 1978. 10.1103/PhysRevA.18.328

[26] Hundertmark, D., Jex, M., Lange, M.: Quantum systems at the brink: existence of bound states, critical potentials, and dimensionality. *Forum Math. Sigma ***11**, (2023). 10.1017/fms.2023.39.

[27] Hundertmark, D., Lee, Y.R.: On non-local variational problems with lack of compactness related to non-linear optics. *J. Nonlinear Sci.* **22**(1), 1–38, 2012. 10.1007/s00332-011-9106-1

[28] Hylleraas, E.A.: Neue Berechnung der Energie des Heliums im Grundzustande, sowie des tiefsten Terms von Ortho-Helium. *Zeitschrift für Physik* **54**(5–6), 347–366, 1929

[29] Ivanov, I.A.: Radius of convergence of the 1/z expansion for the ground state of a two-electron atom. *Phys. Rev. A* **51**, 1080–1084, 1995. 10.1103/PhysRevA.51.1080 9911688 10.1103/physreva.51.1080

[30] King, A.W., Rhodes, L.C., Readman, C.A., Cox, H.: Effect of nuclear motion on the critical nuclear charge for two-electron atoms. *Phys. Rev. A* **91**, 042512, 2015. 10.1103/PhysRevA.91.042512

[31] Klaus, M., Simon, B.: Coupling constant thresholds in nonrelativistic quantum mechanics. I. Short-range two-body case. *Ann. Phys.* **130**(2), 251–281, 1980. 10.1016/0003-4916(80)90338-3

[32] Klaus, M., Simon, B.: Coupling constant thresholds in nonrelativistic quantum mechanics. II. Two-cluster thresholds in -body systems. *Comm. Math. Phys*. **78**(2), 153–168 (1980). http://projecteuclid.org/euclid.cmp/1103908588.

[33] Lieb, E.H.: Bound on the maximum negative ionization of atoms and molecules. *Phys. Rev. A* **29**, 3018–3028, 1984. 10.1103/PhysRevA.29.3018

[34] Lundholm, D.: Some spectral bounds for Schrödinger operators with Hardy-type potentials. Preprint arXiv:0911.3386 p. 11 (2009). https://arxiv.org/abs/0911.3386.

[35] O’Connor, A.J.: Exponential decay of bound state wave functions. *Comm. Math. Phys. ***32**, 319–340 (1973). http://projecteuclid.org/euclid.cmp/1103859181.

[36] Reed, M., Simon, B.: Methods of Modern Mathematical Physics IV. Analysis of Operators. Academic Press, New York-London (1978)

[37] Sergeev, A.V., Kais, S.: Variational principle for critical parameters of quantum systems. *J. Phys. A Math. General* **32**(39), 6891–6896, 1999. 10.1088/0305-4470/32/39/312

[38] Simon, B.: On the absorption of eigenvalues by continuous spectrum in regular perturbation problems. *J. Funct. Anal.* **25**(4), 338–344, 1977. 10.1016/0022-1236(77)90042-8

[39] Simon, B.: Schrödinger semigroups. *Bull. (New Series) Am. Math. Soc.* **7**(3), 447–526 (1982).

[40] Simon, B.: Trace ideals and their applications, Mathematical Surveys and Monographs, vol. 120, second edn. American Mathematical Society, Providence, RI (2005).

[41] Slaggie, E.L., Wichmann, E.H.: Asymptotic properties of the wave function for a bound nonrelativistic three-body system. *J. Math. Phys.* **3**(5), 946–968, 1962. 10.1063/1.1724311

[42] Stillinger, F.H., Stillinger, D.K.: Nonlinear variational study of perturbation theory for atoms and ions. *Phys. Rev. A* **10**, 1109–1121, 1974. 10.1103/PhysRevA.10.1109

[43] Stillinger, F.H., Jr.: Ground-state energy of two-electron atoms. *J. Chem. Phys.* **45**(10), 3623–3631, 1966

[44] Thirring, W.: Quantum mathematical physics, second edn. Springer-Verlag, Berlin (2002). 10.1007/978-3-662-05008-8. Atoms, molecules and large systems, Translated from the 1979 and 1980 German originals by Evans M. Harrell II.

[45] Trudinger, N.S.: Linear elliptic operators with measurable coefficients. *Annali della Scuola Normale Superiore di Pisa - Classe di Scienze Ser. 3*, **27**(2), 265–308 (1973). http://www.numdam.org/item/ASNSP_1973_3_27_2_265_0/.

[46] Zhislin, G.M.: Discussion of the spectrum of schrödinger operators for systems of many particles. *Trudy Moskovskogo Matematiceskogo Obscestva* **9**, 81–120, 1960

